# Higher dose corticosteroids in hospitalised COVID-19 patients requiring ventilatory support (RECOVERY): a randomised, controlled, open-label, platform trial

**DOI:** 10.1016/j.eclinm.2025.103080

**Published:** 2025-02-12

**Authors:** RECOVERY Collaborative Group, O. Abani, A. Abbas, F. Abbas, J. Abbas, K. Abbas, M. Abbas, S. Abbasi, H. Abbass, A. Abbott, N. Abdallah, A. Abdelaziz, M. Abdelfattah, B. Abdelqader, A. Abdul, B. Abdul, S. Abdul, A. Abdul Rasheed, A. Abdulakeem, R. Abdul-Kadir, A. Abdullah, A. Abdulmumeen, R. Abdul-Raheem, N. Abdulshukkoor, K. Abdusamad, Y. Abed El Khaleq, M. Abedalla, A. Abeer Ul Amna, L. Abel, K. Abernethy, M. Abeywickrema, C. Abhinaya, A. Abidin, A. Aboaba, A. Aboagye-Odei, C. Aboah, H. Aboelela, H. Abo-Leyah, K. Abouelela, A. Abou-Haggar, M. Abouibrahim, A. Abousamra, M. Abouzaid, M. Abraham, T. Abraham, A. Abraheem, J. Abrams, R. Abrams, H.J. Abu, A. Abu-Arafeh, S.M. Abubacker, A. Abung, Y. Abusamra, Y. Aceampong, A. Achara, D. Acharya, F. Acheampong, P. Acheampong, S. Acheampong, J. Acheson, S. Achieng, A. Acosta, R. Acquah, C. Acton, J. Adabie-Ankrah, P. Adair, A.S. Adam, F. Adam, M. Adam, H. Adamali, M. Adamczyk, C. Adams, D. Adams, K. Adams, L. Adams, N. Adams, R. Adams, T. Adams, L. Adamu-Ikeme, K. Adatia, K. Adcock, L. Addai-Boampong, A. Addo, O. Adeagbo, A. Adebiyi, O. Adedeji, Y. Adegeye, K. Adegoke, V. Adell, S. Adenwalla, F.W. Adeoye, O.A. Adesemoye, E.O. Adewunmi, A. Adeyanju, J. Adeyemi, T. Adeyemo, B. Adhikari, S.A. Adhikari, R. Adhikary, A. Aditya, P. Adjepong, G. Adkins, A. Adnan, M. Adriaanse, J. Aeron-Thomas, D. Affleck, C. Afnan, M. Afridi, P. Afrim, F.A. Afriyie, Z.A. Aftab, A. Afum-Adjei Awuah, M. Agarwal, P.N. Agasiya, R. Agbeko, C. Agbo, S. Aggarwal, A. Aghababaie, L. Aguilar Jimenez, J.A. Agyekum, K. Agyen, E.K. Ahadome, S. Ahamed Sadiq, M.H. Ahammed Nazeer, M. Ahmad, S. Ahmad, A. Ahmed, B.A.R. Ahmed, B. Ahmed, F. Ahmed, H. Ahmed, I. Ahmed, K. Ahmed, L. Ahmed, M. Ahmed, M.C. Ahmed, M.S. Ahmed, N. Ahmed, N. Ahmed, O. Ahmed, R.A. Ahmed, R. Ahmed, R. Ahmed, S. Ahmed, S.G. Ahmed, S.H. Ahmed, R. Ahmed Ali, B. Ahmed Mohamud, S. Ahmed, S. Ahmer, A. Ahonia, C. Aidoo, C. Aiken, D. Ail, M. Ainsworth, M. Aissa, L. Aitken, B. Ajay, A. Ajibode, A. Ajmi, N. Akhtar, S. Akili, B. Akinbiyi, O. Akindolie, Y. Akinfenwa, O. Akinkugbe, I. Akinpelu, M. Akram, O. Aktinade, U. Akudi, A.S.A.R. Al Aaraj, A. Al Balushi, M. Al Dakhola, A. Al Swaifi, E. Al-Abadi, A. Alabi, N. Aladangady, M. Alafifi, A. Alam, S. Alam, A. Al-Asadi, K. Alatzoglou, P. Albert, A. Albertus, L. Albon, A. Alcala, G. Alcorn, S. Alcorn, A. Aldana, D. Alderdice, A. Aldesouki, R. Aldouri, J. Aldridge, N. Aldridge, R. Ale, R.M. Ale, A. Alegria, A. Alexander, C. Alexander, J. Alexander, P.D.G. Alexander, J. Al-fori, L. Alghazawi, O. Alhabsha, B. Al-Hakim, R. Alhameed, M. Al-Hayali, S. Al-Hity, A. Ali, F.R. Ali, J. Ali, M. Ali, M.S. Ali, N. Ali, O. Ali, R. Ali, S. Ali, E. Aliberti, J. Alin, A. Alina, A. Alipustain, B. Alisjahbana, F. Aliyuda, K. Alizadeh, M. Al-Jibury, S. Al-Juboori, M. Al-Khalil, A. Alkhudhayri, M. Alkhusheh, F. Allan, N. Allan, A. Allanson, R. Allcock, E. Allen, J. Allen, K. Allen, L. Allen, P. Allen, R. Allen, S. Allen, T. Allen, A. Alli, K. Allison, B. Allman, H.K. Allsop, L. Allsop, D. Allsup, A.F.T. Almahroos, H. Al-Moasseb, M. Al-Obaidi, L. Alomari, A. Al-Rabahi, B. Al-Ramadhani, Z. Al-Saadi, R. Al-Sammarraie, I. Alshaer, R. Al-Shahi Salman, W. Al-Shamkhani, F. Alsheikh, B. Al-Sheklly, S. Altaf, A. Alty, M. Alvarez, M. Alvarez Corral, E. Alveyn, M. Alzetani, S. Amamou, N. Amar, S. Ambalavanan, R. Ambrogetti, C. Ambrose, A. Ameen, A. Amelia Ganefianty, K. Ames, M.R. Amezaga, A. Amin, A. Amin, K. Amin, S. Amin, T. Amin, B. Amit, A. Amjad, N. Amjad, J. Amoah-Dankwa, A. Amoako-Adusei, V. Amosun, M. Amsal, K. Amsha, J. Amuasi, N. Amutio Martin, P. Amy, A. Anada, A. Anand, S. Anandappa, S.D. Anantapatnaikuni, N.K.N. Andari, E. Anderson, J. Anderson, L. Anderson, M. Anderson, N. Anderson, R. Anderson, S. Anderson, W. Anderson, P. Andreou, A. Andrews, J. Andrews, K. Aneke, A. Ang, W.W. Ang, T. Angel, A. Angela, P. Angelini, L. Anguvaa, O. Anichtchik, M. Anim-Somuah, K. Aniruddhan, J. Annett, L. Anning, M. Ansah, P.J. Anstey, R. Anstey, A. Anthony, A. Anthony-Pillai, P. Antill, Z. Antonina, V. Anu, M. Anwar, S. Anwar, E. Apetri, A. Apostolopoulos, S. Appleby, D. Appleyard, M.F. Aquino, B. Araba, S. Aransiola, M. Araujo, A. Archer, D. Archer, S. Archer, D. Arcoria, C. Ardley, G. Arhin-Sam, A.-M. Arias, O. Aribike, R. Arimoto, N.L.P.E. Arisanti, C. Arkley, C. Armah, I. Armata, J. Armistead, A. Armitage, C. Armstrong, M. Armstrong, S. Armstrong, W. Armstrong, P. Armtrong, H. Arndt, C. Arnison-Newgass, D. Arnold, R. Arnold, A. Arnott, D. Arora, K. Arora, P. Arora, R. Arora, A. Arter, A. Arthur, N.M. Artini, A. Arumaithurai, A. Arya, R. Arya, D. Aryal, D. Asandei, G.A. Asare, A. Asghar, M. Asghar, A. Ashab, C. Ashbrook-Raby, H. Ashby, J. Ashcroft, S. Ashcroft, G. Asher, Z. Ashfak, A. Ashfaq, H.A. Asiamah, A. Ashish, D. Ashley, S. Ashman-Flavell, S. Ashok, A.-E.-A. Ashour, M.Z. Ashraf, S. Ashraf, M.B. Ashraq, D. Ashton, S. Ashton, A. Ashworth, F.J. Ashworth, R. Ashworth, A. Aslam, I. Aslam, S. Aslam, L. Aslett, H. Asogan, A. Asrar, O. Assaf, R. Astin-Chamberlain, Y.E. Atabudzi, P. Athavale, D. Athorne, B. Atkins, C. Atkins, S. Atkins, J. Atkinson, V. Atkinson, A. Atomode, B. Atraskiewicz, A.A. Attia, E. Attubato, M. Attwood, P. Aubrey, Z. Auer, A. Aujayeb, A.T. Aung, H. Aung, H.W.W. Aung, K.K. Aung, K.T. Aung, N. Aung, Y. Aung, Z.M. Aung, E. Austin, K. Austin, A. Auwal, M. Avari, M. Avery, N. Aveyard, J. Avis, G. Aviss, C. Avram, P. Avram, A. Awadelkareem, G. Awadzi, M. Awaly, A. Awan, S. Awisi, A. Aya, E. Ayaz, J.M. Ayerh, A. Ayers, J. Azam, A. Azeem, M. Azharuddin, A. Aziz, G. Aziz, I. Aziz, N. Aziz, A. Azkoul, A. Azman Shah, G. Azzopardi, H. Azzoug, F. Babatunde, M. Babi, B. Babiker, G. Babington, M. Babirecki, M. Babores, A.O. Babs-Osibodu, T. Bac, S. Bacciarelli, R. Bachar, M.-E. Bachour, A. Bachti, G. Bacon, J. Bacon, B. Badal, A. Badat, M. Bader, G.R. Badhan, S. Badhrinarayanan, J.P. Bae, A. Baggaley, A. Baggott, G. Bagley, D. Bagmane, L. Bagshaw, K. Bahadori, Y. Bahurupi, A. Bailey, J. Bailey, K. Bailey, L. Bailey, M.A. Bailey, M. Bailey, P. Bailey, S. Bailey, H. Baillie, J.K. Baillie, J. Bain, V. Bains, D. Baird, E. Baird, K. Baird, S. Baird, T. Baird, Y. Baird, A. Bajandouh, M. Bajracharya, D.C. Baker, E. Baker, J. Baker, K. Baker, M. Baker, R. Baker, T.-A. Baker, V. Baker, H. Bakere, N. Bakerly, M. Baker-Moffatt, A. Bakhai, N. Bakhtiar, P. Bakoulas, D. Bakthavatsalam, N. Balachandran, A. Balan, P. Balasingam, T. Balaskas, M. Balasubramaniam, N. Balatoni, A. Balcombe, A. Baldwin, A. Baldwin, C. Baldwin, D. Baldwin, F. Baldwin, R. Baldwin-Jones, N. Bale, J. Balfour, M. Ball, Ro Ball, K. Ballard, I. Balluz, C. Balmforth, E. Balogh, A. Baltmr, A. Baluwala, G. Bambridge, A. Bamford, P. Bamford, A. Bamgboye, E. Bancroft, H. Bancroft, J. Banda, K. Bandaru, S. Bandi, N. Bandla, S. Bandyopadhyam, A. Banerjee, R. Banerjee, P. Bang, S. Baniya, O. Bani-Saad, H. Banks, L. Banks, P. Banks, C. Bann, H. Bannister, O. Bannister, L. Banton, D.G. Bao, T. Bao, M. Baptist, T. Baqai, A.M. Baral, S.C. Baral, D. Baramova, R. Barber, E. Barbon, M. Barbosa, J. Barbour, A. Barclay, C. Barclay, G. Bardsley, S. Bareford, S. Bari, M. Barimbing, A. Barker, D. Barker, E. Barker, H. Barker, J. Barker, L. Barker, O. Barker, K. Barker-Williams, S. Barkha, J. Barla, G. Barlow, R. Barlow, V. Barlow, J. Barnacle, A. Barnard, D. Barnes, N. Barnes, R. Barnes, T. Barnes, C. Barnetson, A. Barnett, A. Barnett-Vanes, P.G. Barning, W. Barnsley, A. Barr, D. Barr, J. Barr, C. Barr, N. Barratt, S. Barratt, M. Barrera, A. Barrett, Fi Barrett, J. Barrett, S. Barrett, E. Barrow, J. Bartholomew, M.S. Barthwal, C. Bartlett, G. Bartlett, J. Bartlett, L. Bartlett, S. Bartley, S. Bartolmeu-Pires, A. Barton, G. Barton, J. Barton, L. Barton, R. Barton, R. Baruah, S. Baryschpolec, H. Bashir, A. Bashyal, B. Basker, S. Basnet, B. Basnyat, A. Basoglu, A. Basran, J. Bassett, G. Bassett, C. Bassford, B. Bassoy, V. Bastion, A. Bastola, A. Basumatary, P. Basvi, J.A. Batac, V.R. Bataduwaarachchi, T. Bate, H.J. Bateman, K. Bateman, V. Bateman, E. Bates, H. Bates, M. Bates, S. Bates, S. Batham, A. Batista, A. Batla, D. Batra, H. Batty, T. Batty, A. Batty, M. Baum, R. Baumber, C. Bautista, F. Bawa, T. Bawa, F.S. Bawani, S. Bax, M. Baxter, N. Baxter, Z. Baxter, H. Bayes, L.-A. Bayo, F. Bazari, R. Bazaz, A. Bazli, L. Beacham, W. Beadles, K. Beadon, P. Beak, A. Beale, K. Beard, J. Bearpark, A. Beasley, S. Beattie, K. Beaumont, D. Beaumont-Jewell, T. Beaver, S. Beavis, C. Beazley, S. Beck, V. Beckett, R. Beckitt, S. Beckley, H. Beddall, S. Beddows, D. Beeby, S. Beeby, G. Beech, M. Beecroft, N. Beer, Sa Beer, J. Beety, G. Bega, A. Begg, S. Begg, S. Beghini, A. Begum, S. Begum, S. Begum, T. Behan, R. Behrouzi, J. Beishon, C. Beith, J. Belcher, H. Belfield, K. Belfield, A. Belgaumkar, D. Bell, G. Bell, J. Bell, L. Bell, N. Bell, P. Bell, S. Bell, J. Bellamu, M. Bellamy, T. Bellamy, A. Bellini, A. Bellis, F. Bellis, L. Bendall, N. Benesh, N. Benetti, S.A. Bengu, L. Benham, G. Benison-Horner, S. Benkenstein, T. Benn, A. Bennett, C. Bennett, D. Bennett, G. Bennett, K. Bennett, K. Bennett, L. Bennett, M.R. Bennett, S. Bennett, K. Bennion, G. Benoy, V. Benson, A. Bentley, J. Bentley, I. Benton, E. Beranova, M. Beresford, C. Bergin, M. Bergstrom, J. Bernatoniene, T. Berriman, Z. Berry, F. Best, K. Best, A.-M. Bester, Y. Beuvink, E. Bevan, S. Bevins, T. Bewick, A. Bexley, S. Beyatli, F. Beynon, A. Bhadi, S. Bhagani, S. Bhakta, R. Bhalla, K. Bhandal, K. Bhandal, A. Bhandari, L. Bhandari, L.N. Bhandari, S. Bhandari, J. Bhanich Supapol, A. Bhanot, R. Bhanot, S. Bhasin, A. Bhat, P. Bhat, R. Bhatnagar, K. Bhatt, J. Bhayani, D. Bhojwani, P. Bhuie, M.S. Bhuiyan, S. Bhuiyan, A. Bibby, F. Bibi, N. Bibi, S. Bibi, T. Bicanic, S. Bidgood, J. Bigg, S. Biggs, A. Biju, A. Bikov, S. Billingham, J. Billings, P. Binh, A. Binns, M. BinRofaie, O. Bintcliffe, C. Birch, J. Birch, K. Birchall, S. Bird, M. Birt, C. Bishop, K. Bishop, L. Bishop, K. Bisnauthsing, N. Biswas, M. Bittaye, S. Biuk, K. Blachford, E. Black, H. Black, K. Black, M. Black, P. Black, V. Black, H. Blackgrove, B. Blackledge, J. Blackler, S. Blackley, H. Blackman, C. Blackstock, C. Blair, F. Blakemore, H. Blamey, A. Bland, S. Blane, S. Blankley, P. Blaxill, K. Blaylock, J. Blazeby, N. Blencowe, B. Bloom, J. Bloomfield, A. Bloss, A. Blowers, S. Blows, H. Bloxham, S. Blrd, L. Blundell, A. Blunsum, M. Blunt, T. Blunt, I. Blyth, K. Blyth, A. Blythe, K. Blythe, K.A. Boahen, M. Boampoaa, S. Board, E. Boatemah, B. Bobie, K. Bobruk, P.N. Bodalia, N. Bodasing, M. Boden, T. Bodenham, G. Boehmer, M. Boffito, K. Bohanan, K. Bohmova, N. Bohnacker, S. Bokhandi, M. Bokhar, S. Bokhari, S.O. Bokhari, I. Bokobza, A. Boles, C. Bolger, C. Bonaconsa, C. Bond, H. Bond, S. Bond, T. Bond, A. Bone, G. Boniface, J. Bonney, L. Bonney, L. Booker, S. Boot, M. Boothroyd, J. Borbone, N. Borman, S. Bosence, K. Bostock, N. Botting, F. Bottrill, H. Bouattia, L. Bough, H. Boughton, Z. Boult, T. Boumrah, M. Bourke, S. Bourke, M. Bourne, R. Bousfield, L. Boustred, A. Bowes, P. Bowker, T. Bowker, H. Bowler, L. Bowman, S. Bowman, R. Bowmer, A. Bowring, H. Bowyer, A. Boyd, J. Boyd, L. Boyd, N. Boyer, N. Boyle, P. Boyle, R. Boyle, L. Boyles, L. Brace, A. Bracken, J. Bradder, C.J. Bradley, P. Bradley, J. Bradley-Potts, L. Bradshaw, Z. Bradshaw, C. Brady, R. Brady, S. Brady, P. Braga Sardo, D. Braganza, M. Braithwaite, S. Brammer, M. Branch, T. Brankin-Frisby, J. Brannigan, S. Brattan, F. Bray, N. Bray, M. Brazil, L. Brear, Tr Brear, S. Brearey, L. Bremner, M. Brend, C. Bresges, C. Bressington, G. Bretland, C. Brewer, M. Bridgett, G. Bridgwood, S. Brigham, J. Bright, C. Brightling, T. Brigstock, L. Brimfield, P. Brinksman, E. Brinkworth, R. Brittain-Long, V. Britten, H. Britton, L. Broad, S. Broadhead, R. Broadhurst, A. Broadley, M. Broadway, C. Brockelsby, M. Brocken, T. Brockley, M. Brodsky, F. Brogan, L. Brohan, F. Brokke, J. Brolly, D. Bromley, H. Brooke-Ball, V. Brooker, M. Brookes, D. Brooking, A. Brooks, D. Brooks, J. Brooks, K. Brooks, N. Brooks, P. Brooks, R. Brooks, S. Brooks, M. Broom, N. Broomhead, C. Broughton, N. Broughton, M. Brouns, A. Brown, C. Brown, E. Brown, H. Brown, J. Brown, L. Brown, N. Brown, P. Brown, R. Brown, S. Brown, T. Brown, B. Browne, C. Browne, D. Browne, M. Browne, S. Brownlee, A. Brraka, J. Bruce, M. Bruce, W. Brudlo, A. Brunchi, N. Brunskill, A. Brunton, M. Brunton, M. Bryant, E. Bryden, H. Brzezicki, A. Buazon, M.H. Buch, R. Buchan, R. Buchanan, D. Buche, A. Buck, L. Buck, M. Buckland, C. Buckley, L. Buckley, P. Buckley, S. Buckley, C. Buckman, A. Budds, G. Bugg, R. Bujazia, M. Bukhari, S. Bukhari, R. Bulbulia, A. Bull, D. Bull, K. Bull, R. Bull, Th Bull, E. Bullock, S. Bullock, N. Bulteel, K. Bumunarachchi, R. Bungue-Tuble, O. Burbidge, C. Burchett, D. Burda, C. Burden, T.G. Burden, Mi Burgess, R. Burgess, S. Burgess, E. Burhan, H. Burhan, H. Burke, K. Burke, A. Burman, S. Burnard, C. Burnett, S. Burnett, A. Burns, C. Burns, J. Burns, K. Burns, D. Burrage, K. Burrows, C. Burston, A. Burton, B. Burton, F. Burton, H. Burton, M. Burton, M. Butar butar, D. Butcher, A. Butler, E. Butler, J. Butler, P. Butler, S. Butler, J. Butler, A.-T. Butt, M. Butt, M.M. Butt, C. Butterworth, N. Butterworth-Cowin, R. Buttery, T. Buttle, H. Button, D. Buttress, H. Bye, J. Byrne, W. Byrne, V. Byrne-Watts, N.K. C, A. Cabandugama, L. Cabrero, S. Caddy, R. Cade, A. Cadwgan, Z. Cahilog, A. Cahyareny, D. Cairney, J. Calderwood, D. Caldow, E. Cale, G. Calisti, D. Callaghan, J. Callaghan, C. Callens, D. Callum, C. Calver, M. Cambell-Kelly, T. Camburn, D.R. Cameron, E. Cameron, F. Cameron, S. Cameron, C. Camm, F.D. Cammack, A. Campbell, B. Campbell, D. Campbell, H. Campbell, J. Campbell, K. Campbell, M. Campbell, R. Campbell, W. Campbell, Q. Campbell Hewson, J. Camsooksai, L. Canclini, S.M. Candido, J. Candlish, C. Caneja, A. Cann, J. Cann, R. Cannan, A. Cannon, E. Cannon, M. Cannon, P. Cannon, V. Cannons, E. Canonizado, J. Cantliff, N. Cap, N.T. Cap, B. Caplin, S. Capocci, N. Caponi, A. Capp, R. Capstick, T. Capstick, C. Caraenache, A. Card, M. Cardwell, C. Carey, R. Carey, S. Carley, F. Carlin, T. Carlin, S. Carmichael, M. Carmody, M. Carnahan, C. Caroline, J. Carpenter, S. Carr, A. Carrasco, Z. Carrington, A. Carroll, P. Carroll, R. Carson, C. Cart, E. Carter, J. Carter, M. Carter, N. Carter, P. Carter, D. Cartwright, J.-A. Cartwright, C. Carty, L. Carty, J. Carungcong, C. Carver, E. Carver, R. Carver, S. Casey, A. Cassells, T. Castiello, G. Castle, B. Castles, M. Caswell, A.M. Catana, H. Cate, A. Catelan Zborowski, S. Cathcart, K. Cathie, D. Catibog, C. Catley, L. Catlow, M. Caudwell, A. Cavazza, A. Cave, L. Cave, S. Cavinato, F. Cawa, K. Cawley, C. Caws, K. Cawthorne, H. Cendl, H. Century, J. Cernova, M. Cesay, E. Cetti, S. Chabane, M. Chablani, C. Chabo, J. Chacko, D. Chadwick, J. Chadwick, R. Chadwick, E. Chakkarapani, A. Chakraborty, M. Chakraborty, M. Chakravorty, P. Chalakova, B. Chalise, B.S. Chalise, J. Chalmers, R. Chalmers, G. Chamberlain, S. Chamberlain, E. Chambers, J. Chambers, L. Chambers, N. Chambers, A. Chan, C. Chan, E. Chan, M. Chan, K. Chan, P. Chan, R. (P-C). Chan, X.H.S. Chan, C. Chandler, H. Chandler, K.J. Chandler, S. Chandler, Z. Chandler, S. Chandra, N. Chandran, B. Chandrasekaran, Y. Chang, H. Chanh, H.Q. Chanh, G. Chaplin, J. Chaplin, G. Chapman, J. Chapman, K. Chapman, L. Chapman, M. Chapman, P. Chapman, T. Chapman, L. Chappell, A. Charalambou, B. Charles, D. Charlton, S. Charlton, K. Chatar, C. Chatha, D. Chatterton, N. Chau, R. Chaube, A. Chaudhary, M.Y.N. Chaudhary, B. Chaudhary, I. Chaudhry, Z. Chaudhry, K. Chaudhuri, N. Chaudhuri, M. Chaudhury, A. Chauhan, R.S. Chauhan, L. Chaulagain, A. Chavasse, N. Chavasse, V. Chawla, L. Cheater, J. Cheaveau, C. Cheeld, M. Cheeseman, F. Chen, H.M. Chen, T. Chen, F. Cheng, L.Y. Cheng, Z. Cheng, H. Chenoweth, C.H. Cheong, J.J. Cherian, S. Cherian, M. Cherrie, H. Cheshire, C.K. Cheung, E. Cheung, K. Cheung, M. Cheung, C. Cheyne, S. Chhabra, W.L. Chia, E. Chiang, A. Chiapparino, R. Chicano, G. Chikara, M. Chikungwa, Z.A. Chikwanha, G. Chilcott, S. Chilcott, A. Chilvers, P. Chimbo, K.W. Chin, W.J. Chin, R. Chineka, A. Chingale, E. Chinonso, C. Chin-Saad, M. Chirgwin, H. Chisem, C. Chisenga, C. Chisholm, B. Chisnall, C. Chiswick, S. Chita, N. Chitalia, M. Chiu, L. Chiverton, B. Chivima, C. Chmiel, S. Choi, W. Choon Kon Yune, M. Chopra, V. Choudhary, O. Choudhury, S. Choudhury, B.-L. Chow, M. Chowdhury, S. Chowdhury, A. Chrisopoulou, V. Christenssen, P. Christian, A. Christides, F. Christie, D. Christmas, G. Christoforou, T. Christopherson, A. Christou, M. Christy, P. Chrysostomou, Y. Chua, D. Chudgar, R. Chudleigh, S. Chukkambotla, M.E. Chukwu, I. Chukwulobelu, C.Y. Chung, E. Church, S.R. Church, D. Churchill, N. Cianci, P. Cicconi, P. Cinardo, Z. Cipinova, B. Cipriano, S. Clamp, B. Clancy, M. Clapham, E. Clare, S. Clare, A. Clark, C. Clark, D. Clark, E. Clark, F. Clark, G. Clark, J. Clark, K. Clark, L. Clark, M. Clark, N. Clark, P. Clark, R. Clark, T. Clark, Z. Clark, A. Clarke, J. Clarke, P. Clarke, R. Clarke, S. Clarke, A. Claxton, L. Claxton, K. Clay, C. Clayton, E. Clayton, O. Clayton, J. Clayton-Smith, B. Clearyb, C. Cleaver, R. Cleeton, I. Clement, C. Clemente de la Torre, J. Clements, S. Clements, S. Clenton, S. Cliff, R. Clifford, S. Clifford, A. Clive, J. Clouston, V. Clubb, S. Clueit, L. Clutterbuck, A. Clyne, M. Coakley, P.G.L. Coakley, K. Cobain, A. Cochrane, P. Cochrane, L. Cockayne, M. Cockerell, H. Cockerill, S. Cocks, R. Codling, A. Coe, S. Coetzee, D. Coey, D. Cohen, J. Cohen, O. Cohen, M. Cohn, L. Coke, O. Coker, N. Colbeck, R. Colbert, E. Cole, J. Cole, G. Coleman, M. Coleman, N. Coleman, H. Coles, M. Colin, A. Colino-Acevedo, J. Colley, K. Collie, A. Collier, D. Collier, H. Collier, T. Collingwood, P. Collini, E. Collins, J. Collins, K. Collins, M. Collins, N. Collins, S. Collins, V. Collins, A. Collinson, B. Collinson, J. Collinson, M. Collis, M. Colmar, H.E. Colton, J. Colton, K. Colville, C. Colvin, E. Combes, D. Comer, A. Comerford, D. Concannon, A. Condliffe, R. Condliffe, E. Connell, L. Connell, N. Connell, K. Connelly, G. Connolly, E. Connor, A. Conroy, K. Conroy, V. Conteh, R. Convery, F. Conway, G. Conway, R. Conway, J.-A. Conyngham, A. Cook, C. Cook, E. Cook, G. Cook, H. Cook, J. Cook, M. Cook, S. Cook, D. Cooke, G. Cooke, H. Cooke, J. Cooke, K. Cooke, T. Cooke, V. Cooke, A. Cooper, C. Cooper, D. Cooper, H. Cooper, J. Cooper, L. Cooper, N. Cooper, R. Cooper, S. Cooray, T. Cope, S. Corbet, C. Corbett, A. Corbishley, J. Corcoran, C. Cordell, J. Cordle, A. Corfield, J. Corless, A. Corlett, J. Cornwell, M. Cornwell, D. Corogeanu, A. Corr, M. Corredera, R. Corrigan, P. Corry, R. Corser, J. Cort, D. Cosgrove, T. Cosier, P. Costa, T. Costa, C. Coston, S. Cotgrove, Z. Coton, L.-J. Cottam, R. Cotter, D. Cotterill, C. Cotton, G. Couch, M. Coulding, A. Coull, D. Counsell, D. Counter, C. Coupland, E. Courtney, J. Courtney, R. Cousins, A.J. Coutts, A. Cowan, E. Cowan, R. Cowan, R. Cowell, L. Cowen, S. Cowman, A. Cowton, E. Cox, G. Cox, H. Cox, K. Cox, M. Cox, K. Coy, A. Cradduck-Bamford, H. Craig, J. Craig, V. Craig, F. Craighead, M. Cramp, H. Cranston, S.S. Crasta, J. Crause, A. Crawford, E. Crawford, I. Crawford, S. Crawshaw, B. Creagh-Brown, A. Creamer, A. Creaser-Myers, J. Cremona, S. Cremona, A. Crepet, J. Cresswell, M. Cribb, C. Crichton, D. Crilly, L. Crisp, N. Crisp, D. Crocombe, M. Croft, J. Crooks, H. Crosby, E. Cross, T. Cross, A. Crothers, S. Crotty, S. Crouch, M. Crow, A. Crowder, K. Crowley, T. Crowley, R. Croysdill, C. Cruickshank, I. Cruickshank, J. Cruise, C. Cruz, T. Cruz Cervera, D. Cryans, G. Cui, H. Cui, L. Cullen, G. Cummings-Fosong, V. Cunliffe, N. Cunningham, J. Cupitt, H. Curgenven, G. Curnow, D. Curran, S. Curran, C. Currie, J. Currie, S. Currie, J. Curtis, K. Curtis, M. Curtis, O. Curtis, T. Curtis, R. Cuthbertson, J. Cuthill, S. Cutler, S. Cutts, M. Czekaj, P. Czylok, S. D’Souza, J. da Rocha, G.S. Dadzie, M. Dafalla, A. Dagens, H. Daggett, J. Daglish, S. Dahiya, A. Dale, K. Dale, M. Dale, S. Dale, J. Dales, U. D'Alessandro, H. Dalgleish, H. Dallow, C. D'aloia, D. Dalton, M. Dalton, Z. Daly, M. Damani, E. Damm, L. Dan, A. Danga, J. Dangerfield, A. Daniel, P. Daniel, A. Daniels, A. Dann, K.G. Danso, S. Danso-Bamfo, Q.T. Dao, S. Darby, A. Darbyshire, J. Darbyshire, P. Dargan, P. Dark, K. Darlington, S. Darnell, T. Darton, G. Darylile, A. Das, M. Das, S. Das, M. Daschel, J. Dasgin, D. Datta, A. Daunt, V. Dave, E. Davenport, M. Davey, M. David, A. Davidson, L. Davidson, N. Davidson, R. Davidson, A. Davies, B. Davies, C. Davies, D. Davies, E. Davies, F. Davies, G. Davies, H. Davies, J. Davies, K. Davies, L. Davies, M. Davies, N. Davies, O. Davies, P. Davies, R. Davies, S. Davies, A. Davis, J.-A. Davis, K. Davis, P. Davis, A. Davis-Cook, A. Davison, C. Dawe, H. Dawe, M. Dawkins, A. Dawson, D. Dawson, E. Dawson, J. Dawson, L. Dawson, M. Dawson, S. Dawson, T. Dawson, I. Dawson, A. Daxter, A. Day, J. Day, J. D'Costa, P. De, D. de Fonseka, T. de Freitas, P. De Los Santos Dominguez, R. De Pretto, F. De Santana Miranda, E. de Sausmarez, S. de Silva, T. de Silva, J. De Sousa, P. De Sousa, J. de Souza, P. De Souza, A. De Soyza, N. de Vere, J. de Vos, B. Deacon, S. Dealing, A. Dean, J. Dean, K. Dean, S. Dean, T. Dean, J. Deane, J. Dear, E. Dearden, C. Deas, S. Debbie, G. Debreceni, V. Deelchand, M. Deeley, J. Deery, E. Defever, M. Del Forno, A. Dela Rosa, G. De-La-Cedra, A. Dell, C. Demetriou, D. DeMets, J. Democratis, J. Denham, E. Denis, L. Denley, C. Denmade, A. Dent, K. Dent, M. Dent, E. Denton, T. Denwood, N. Deole, D. Depala, M. Depante, S. Dermody, A. Desai, P. Desai, S. Deshpande, V. Deshpande, S. Devkota, U. Devkota, D. Devonport, M. Devonport, P. Dey, V. Dey, R. Deylami, K. Dhaliwal, P. Dhangar, S. Dhani, A. Dhanoa, M. Dhar, A. Dhariwal, D. Dharmasena, D. Dhasmana, E. Dhillon, R. Dhillon, S. Dhillon, M. Dhimal, D. Dhiru, T. Dhorajiwala, P. Dias, S. Diaz, K. Diaz-Pratt, M. Dibas, D. Dickerson, P. Dicks, M. Dickson, S. Dickson, J. Digby, R. Digpal, S. Dillane, S. Diment, P. Dimitri, G. Dimitriadis, S. Din, T.H. Dinh, T.T.T. Dinh, C. Dipheko, A. Dipper, S. Dipro, L. Dirmantaite, L. Dismore, L. Ditchfield, S. Diver, L. Diwakar, P. Diwan, C. Dixon, G. Dixon, K. Dixon, B. Djeugam, S. Dlamini, P. Dlouhy, A. D'Mello, P. Dmitri, T. Do, T.T. Do, L. Dobbie, M. Dobranszky Oroian, C. Dobson, L. Dobson, M. Docherty, D. Dockrell, J. Dodd, J. Dodds, R. Dodds, S. Dodds, R. Dogra, C. Doherty, E. Doherty, W. Doherty, Y. Doi, I. Doig, E. Doke, D. Dolan, M. Dolman, R. Dolman, L. Donald, K. Donald, C. Donaldson, D. Donaldson, G. Donaldson, K. Donaldson, P. Dong, P.K. Dong, M. Donkor, S. Donlon, J. Donnachie, E. Donnelly, R. Donnelly, P. Donnison, A. Donohoe, G. Donohoe, B. Donohue, E. Dooks, R. Doonan, R. Doorn, G. Doran, R. Dore, K. Dorey, S. Dorgan, K. Dos Santos, M. Dosani, D. Dosanjh, P. Dospinescu, I. Doss, T. Doudouliaki, A. Dougherty, J. Doughty, K. Douglas, J. Douse, A. Dow, L. Dowden, M. Dower, S. Dowling, N. Downer, C. Downes, R. Downes, T. Downes, D. Downey, R. Downey, C. Downing, L. Downs, S. Dowson, C. Dragan, C. Dragos, M. Drain, C. Drake, V. Drew, O. Drewett, A. Drexel, C. Driscoll, H. Drogan, O. Drosos, G. Drummond, K. Drury, K. Druryk, R. Druyeh, J. Dryburgh-Jones, S. Drysdale, P. Dsouza, A. Du Thinh, I.K. Duah, H. Dube, J. Dube, S. Duberley, P. Duckenfield, H. Duckles-Leech, N. Duff, E. Duffield, H. Duffy, K. Duffy, L. Dufour, A. Duggan, P. Dugh, R. Duhoky, J. Duignan, J. Dulay, S. Dummer, A. Duncan, C. Duncan, F. Duncan, G. Duncan, H. Duncan, R. Duncan, S. Dundas, D.V. Dung, N.T.P. Dung, A. Dunleavy, J. Dunleavy, A. Dunn, C. Dunn, D. Dunn, L. Dunn, P. Dunn, C. Dunne, K. Dunne, F. Dunning, A. Dunphy, T. Duong, T.T.H. Duong, V. Duraiswamy, B. Duran, I. DuRand, L. Durdle, N. Duric, A. Durie, E. Durie, S. Durogbola, C. Durojaiye, L. Durrans, K. Durrant, H. Durrington, I. Duru, H. Duvnjak, A. Dwarakanath, L. Dwarakanath, D. Dwomoh Nkrumah, E. Dwyer, Z. Dyar, C. Dyball, K. Dyer, H. Dymond, T. Dymond, E.D. Dzidzomu, C. Eades, L. Eadie, R. Eadie, L. Eagles, B. Eapen, N. Earl, J. Early, M. Earwaker, N. Easom, C. East, A. Easthope, F. Easton, J. Easton, P. Easton, R. Eatough, O. Ebigbola, M. Ebon, A. Eccles, S. Eccles, C. Eddings, M. Eddleston, M. Edgar, K. Edgerley, N. Edmond, M. Edmondson, T. Edmunds, A. Edwards, C. Edwards, J. Edwards, K. Edwards, M. Edwards, S. Edwards, J. Eedle, A. Eggink, S. Eggleston, L. Ehiorobo, S. Eisen, M. Ekoi, A. Ekunola, N. Elashbar, L. Elawamy, D. Eleanor, S. El Behery, M. Elbeshy, K. El-Bouzidi, M. El-Din, E. Eldridge, U. Elenwa, I. Eletu, E. Elfar, M. Elgamal, A. Elgohary, N. Elkaram, R. Elmahdi, S. Eliammmknjhhhh, J. Elias, T. Elias, A. Elkins, J. Ellam, L. Ellerton, L. Elliot, A. Elliott, F. Elliott, K. Elliott, S. Elliott, A. Ellis, C. Ellis, K. Ellis, L. Ellis, R. Ellis, T. Ellis, T.-Y. Ellis, Y. Ellis, A. Ellwood, R. Elmahdi, E. Elmahi, H.-M. Elmasry, A. Emery, M. El-Naggar, N. Elndari, O. Elneima, M. Elokl, A. Elradi, M. Elsaadany, M.A.S.A. Elsayed, S. El-Sayeh, H. El-Sbahi, M. Elsebaei, T. Elsefi, K. El-Shakankery, A. Elsheikh, H. El-Taweel, S. Elyoussfi, J. Emberey, J.R. Emberson, J. Emberton, A. Emery, J. Emmanuel, I. Emmerson, M. Emms, F. Emond, M. Emonts, N. Enachi, D. Enenche, A. Engden, K. English, C. Enimpah, E. Entwistle, H. Enyi, M. Erotocritou, P. Eskander, H. Esmail, F. Essa, B. Evans, C. Evans, D. Evans, E. Evans, G. Evans, I. Evans, J. Evans, L. Evans, M. Evans, R. Evans, S. Evans, T. Evans, C. Everden, S. Everden, L. Every, H. Evison, L. Evison, C. Ezenduka, J. Faccenda, L. Fahel, Y. Fahmay, I. Fairbairn, S. Fairbairn, T. Fairbairn, A. Fairclough, L. Fairlie, M. Fairweather, A. Fajardo, N. Falcone, E. Falconer, J. Fallon, A. Fallow, D. Faluyi, V. Fancois, A. Farah, M. Farah, Q. Farah, N.Z. Fard, L. Fares, A. Farg, A. Farmer, K. Farmer, T. Farmery, S. Farnworth, F. Farook, H. Farooq, S. Farooq, F. Farquhar, H. Farr, A. Farrell, B. Farrell, F. Farrukh, J. Farthing, S. Farzana, R. Fasina, A. Fatemi, M. Fatemi, S. Fathima, N. Fatimah, M. Faulkner, S. Faust, C. Favager, A. Fawad, J. Fawke, S. Fawohunre, A. Fazal, A. Fazleen, S. Fearby, C. Fearnley, A. Feben, F. Fedel, D. Fedorova, C. Fegan, M. Felongo, L. Felton, T. Felton, K. Fenlon, A. Fenn, R. Fennelly, I. Fenner, C. Fenton, M. Fenton, G. Ferenando, C. Ferguson, J. Ferguson, K. Ferguson, S. Ferguson, V. Ferguson, D. Fernandes, C. Fernandez, E. Fernandez, M. Fernandez, S. Fernandez Lopez, J. Fernandez Roman, C.J. Fernando, J. Fernando, A. Feroz, P. Ferranti, T. Ferrari, E. Ferrelly, A. Ferrera, E. Ferriman, S. Ferron, N. Fethers, B. Field, J. Field, R. Field, K. Fielder, L. Fieldhouse, A. Fielding, J. Fielding, S. Fielding, A. Fikree, S. Filipa, S. Filson, S. Finan, S. Finbow, D.J. Finch, J. Finch, L. Finch, S. Finch, N. Fineman, J. Finlayson, L. Finlayson, A. Finn, J. Finn, D. Finnerty, C. Finney, D. Finucane, S. Fiouni, J. Fiquet, P. Firi, J. Fisher, N. Fisher, D. Fishman, K. Fishwick, C. Fitton, F. Fitzgerald, K. Fitzjohn, J. Flaherty, M. Flanagan, C. Flanders, N. Flaris, G. Fleming, J. Fleming, L. Fleming, P. Fleming, W. Flesher, A. Fletcher, J. Fletcher, L. Fletcher, S. Fletcher, F. Flett, K. Flewitt, S. Flockhart, C. Flood, I. Floodgate, J. Flor, V. Florence, M. Flowerdew, S. Floyd, M.J. Flynn, R. Flynn, C. Foden, A. Fofana, G. Fogarty, P. Foley, L. Folkes, T. Fong, D.M. Font, A. Foo, J. Foo, A. Foot, H.R. Foot, J. Foot, J. Forbes, A. Ford, J. Ford, I. Fordham, J. Foreman, M. Forester, M. Forkan, C. Fornolles, A. Forrest, E. Forsey, M. Forsey, T. Forshall, E. Forster, A. Forsyth, J. Forton, C. Foster, E. Foster, J. Foster, R.A. Foster, T. Foster, A. Foulds, I. Foulds, F. Fowe, N. Fowkes, E. Fowler, R. Fowler, S. Fowler, A. Fox, C. Fox, H. Fox, J. Fox, L. Fox, L. Fox, N. Fox, O. Fox, S. Fox, S.-J. Foxton, E. Fraile, R. Frake, A. Francioni, O. Francis, R. Francis, S. Francis, T. Francis-Bacon, H. Frankland, J. Franklin, S. Franklin, C. Fraser, A. Fratila, S. Frayling, M. Fredlund, A. Freeman, C. Freeman, E. Freeman, H. Freeman, N. Freeman, C. Freer, E. French, T. French, K. Freshwater, M. Frise, R. Fromson, A. Frosh, J. Frost, V. Frost, O. Froud, R. Frowd, A. Fryatt, A. Frygier, B. Fuller, L. Fuller, T. Fuller, D. Fullerton, C. Fung, G. Fung, S. Funnell, J. Furness, A. Fyfe, N. G, E. Gabbitas, C. Gabriel, Z. Gabriel, H. Gachi, S. Gaffarena, S. Gage, J. Gahir, S. Gajebasia, K. Gajewska-Knapik, B. Gajmer, Z. Galani, C. Gale, H. Gale, L. Gale, R. Gale, S. Gali, B. Gallagher, J. Gallagher, R. Gallagher, W. Gallagher, F. Gallam, J. Galliford, C. Galloway, E. Galloway, J. Galloway, A. Galvin, V. Galvis, G. Gamble, L. Gamble, B. Gammon, C.N. Gan, M.B. Ganaie, J. Ganapathi, R. Ganapathy, K. Gandhi, S. Gandhi, U. Ganesh, T. Ganeshanathan, S. Ganguly, A. Gani, P. Ganley, U. Garcia, E.-J. Garden, A.D. Gardener, E. Gardiner, M. Gardiner, P. Gardiner, S. Gardiner, C. Gardiner-Hill, J. Gardner, L. Gardner, M. Garfield, A. Garg, I. Garg, N. Garlick, D. Garner, J. Garner, L. Garner, Z. Garner, R. Garr, K.A. Garrero, M. Gartaula, F. Garty, R. Gascoyne, H. Gashau, A. Gatenby, E. Gaughan, A. Gaurav, M. Gavrila, J. Gaylard, S. Gayle, C. Geddie, I. Gedge, S. Gee, F. Geele, K. Geerthan, M. Gellamucho, K. Gelly, L. Gelmon, S. Gelves-Zapata, G. Genato, N. Gent, S. Gent, N. Geoghegan, A. George, B. George, S. George, T. George, V.P. George, S. Georges, D. Georgiou, P. Gerard, L. Gerdes, L. Germain, H. Gerrish, A. Getachew, L. Gethin, S. Gettings, H. Ghanayem, B. Ghavami Kia, S. Ghazal, A. Gherman, A. Ghosh, D. Ghosh, J. Ghosh, S. Ghosh, T. Giang, T.V. Giang, S. Giannopoulou, M. Gibani, C. Gibb, B. Gibbison, K. Gibbons, A. Gibson, B. Gibson, J. Gibson, K. Gibson, S. Gibson, M. Gigi, C. Gilbert, J. Gilbert, K. Gilbert, B. Giles, J. Gilham, M. Gill, L. Gill, P. Gillen, A. Gillesen, K. Gillespie, E. Gillham, A. Gillian, D. Gilliland, R. Gillott, D. Gilmour, K. Gilmour, L. Gilmour, L. Ginn, F. Ginting, T. Giokanini-Royal, A. Gipson, B. Giri, J. Girling, R. Gisby, A. Gkioni, A. Gkoritsa, E. Gkrania-Klotsas, A. Gladwell, J. Glanville, J. Glasgow, S. Glasgow, J. Glass, L. Glass, S. Glaysher, L. Gledhill, E. Glenday, A. Glennon, J. Glossop, J. Glover, K. Glover, M. Glover, J. Glover Bengtsson, D. Glowski, S. Glynn, C. Gnanalingam, J. Goddard, W. Goddard, E. Godden, J. Godden, S. Godlee, E. Godson, G. Godwin, S. Gogoi, A. Goh, M. Gohel, R. Goiriz, S. Gokaraju, R. Goldacre, A. Goldsmith, P. Goldsmith, D. Gomersall, L. Gomez, R. Gomez-Marcos, A. Gondal, C. Gonzalez, J. Goodall, V. Goodall, B. Goodenough, A. Goodfellow, L. Goodfellow, J. Goodlife, C. Goodwin, E. Goodwin, J. Goodwin, P. Goodyear, R. Gooentilleke, M. Goonasekara, S. Gooseman, S. Gopal, C. Gordon, S. Gordon, R. Gore, H. Gorick, C. Gorman, S. Gormely, M. Gorniok, D. Gorog, M. Gorst, T. Gorsuch, J. Gosai, R. Gosling, S. Gosling, G. Gosney, V. Goss, D. Gotham, N. Gott, E. Goudie, N. Gould, S. Gould, C. Goumalatsou, L. Gourbault, A. Govind, R. Govindan, S. Gowans, G. Gowda, R. Gowda, H. Gower, P. Goyal, S. Goyal, C. Graham, J. Graham, L. Graham, R. Graham, S. Graham, M. Graham-Brown, J. Grahamslaw, G. Grana, T. Grandison, L. Grandjean, A. Grant, D. Grant, K. Grant, M. Grant, P. Grant, R. Gravell, J. Graves, A. Gray, C. Gray, G. Gray, J. Gray, K. Gray, N. Gray, R. Gray, S. Gray, A. Grayson, F. Greaves, P. Greaves, A. Green, A.S. Green, C. Green, C.A. Green, D. Green, F. Green, J. Green, M. Green, N. Green, S. Green, D. Greene, P. Greenfield, A. Greenhalgh, D. Greenwood, S. Greer, J. Gregory, K. Gregory, T. Gregory, J. Greig, R. Grenfell, T. Grenier, J. Grenville, J. Gresty, S. Grevatt, G. Grey, S. Gribben, A. Gribbin, A. Gribble, N. Grieg, D. Grieve, B. Griffin, D. Griffin, M. Griffin, S. Griffith, A. Griffiths, D. Griffiths, I. Griffiths, M. Griffiths, N. Griffiths, O. Griffiths, S. Griffiths, Y. Griffiths, S. Grigoriadou, S. Grigsby, P. Grist, E. Grobovaite, D. Grogono, C. Grondin, R. Groome, P. Grose, L. Grosu, J. Grounds, M. Grout, H. Grover, J. Groves, N. Grubb, J. Grundy, F. Guarino, S. Gudur, J. Guerin, S. Guettari, S. Gulati, V. Gulia, H. Gunasekara, P. Gunasekera, M. Gunawardena, K. Gunganah, J. Gunn, E. Gunter, A. Gupta, A.K. Gupta, A. Gupta, R. Gupta, T. Gupta, V. Gupta, A. Gupta-Wright, V. Guratsky, A. Gureviciute, S. Gurram, A. Gurung, B. Gurung, L. Gurung, S. Gurung, S. Gurung Rai, H. Guth, N. Guthrine, S. Gyambrah, P. Gyanwali, S. Gyawali, N. Ha, N.T. Ha, N.X. Ha, R. Habibi, B. Hack, J. Hackett, P. Hackney, C. Hacon, A. Haddad, D. Hadfield, N. Hadfield, S. Hadfield, M. Hadjiandreou, N. Hadjisavvas, A. Haestier, N. Hafiz, R. Hafiz-Ur-Rehman, J. Hafsa, S. Hagan, J.W. Hague, R. Hague, N. Haider, K. Haigh, V. Haile, J. Hailstone, C. Haines, S. Hainey, M. Hair, B. Hairsine, J. Hajnik, D. Hake, L. Hakeem, A. Haldeos, W. Halder, E. Hale, J. Hale, C. Halevy, P. Halford, W. Halford, A. Halim, A. Hall, C. Hall, E. Hall, F. Hall, H. Hall, J. Hall, K. Hall, L. Hall, J. Hallas, K. Hallas, C. Hallett, J. Halliday, A. Hallman, H. Halls, M. Hamdollah-Zadeh, I.A. Hamed-Adekale, B. Hameed, M. Hameed, R. Hamers, I. Hamid, M. Hamie, R. Hamill, B. Hamilton, F. Hamilton, G. Hamilton, L. Hamilton, M. Hamilton, N. Hamilton, S. Hamilton, R. Hamlin, E. Hamlyn, B. Hammans, S. Hammersley, K. Hammerton, B. Hammond, E. Hammond, L. Hammond, S. Hammond, F. Hammonds, I. Hamoodi, K. Hampshire, J.A. Hampson, J. Hampson, L. Hampson, L. Hamzah, J. Han, O. Hanci, S. Hand, L. Handayani, J. Handford, S. Handrean, N.K. Handzewniak, S. Haney, D.T.T. Hang, V. Hang, V.T.K. Hang, D. Hanh, S. Hanif, E. Hanison, J. Hannah, A. Hannington, M. Hannun, A. Hanrath, H. Hanratty, D. Hansen, A. Hanson, H. Hanson, J. Hanson, K. Hanson, S. Hanson, N. Hao, A. Haqiqi, M. Haque, H. Harcourt, L. Harden, Z. Harding, S. Hardman, M. Hardwick, G. Hardy, J. Hardy, Y. Hardy, K. Haresh, R. Harford, B. Hargadon, J. Hargraves, C. Hargreaves, A. Harin, M. Haris, E. Harlock, P. Harman, T. Harman, M. Harmer, M.A. Haroon, C. Harper, H. Harper, J. Harper, P. Harper, R. Harper, S. Harrhy, K. Harrington, S. Harrington, Y. Harrington-Davies, J. Harris, L. Harris, M.-C. Harris, N. Harris, S. Harris, D. Harrison, L. Harrison, M. Harrison, O.A. Harrison, R. Harrison, S. Harrison, T. Harrison, W. Harrison, E. Harrod, C. Hart, D. Hart, J. Hartley, L. Hartley, R. Hartley, T. Hartley, W. Hartrey, P. Hartridge, S. Hartshorn, A. Harvey, M. Harvey, C. Harwood, H. Harwood, Z. Harzeli, B. Haselden, H. Hasford, K. Hashem, M. Hashimm, T. Hashimoto, I. Hashmi, J. Haslam, Z. Haslam, G. Hasnip, A. Hassan, Z. Hassan, S. Hassasing, J. Hassell, P. Hassell, A. Hastings, B. Hastings, J. Hastings, S. Hathaway-Lees, J. Hatton, J. Hau, M. Havinden-Williams, S. Havlik, D.B. Hawcutt, K. Hawes, L. Hawes, N. Hawes, L. Hawker, A. Hawkins, J. Hawkins, N. Hawkins, W. Hawkins, D. Hawley, E. Hawley-Jones, E. Haworth, A.W. Hay, C. Hay, A. Hayat, J. Hayat, M.-R. Hayathu, A. Hayes, J. Hayes, K. Hayes, M. Hayes, F. Hayes, P. Hayle, C. Haylett, A. Hayman, M. Hayman, M. Haynes, R. Haynes, R. Hayre, C. Hays, S. Haysom, J. Hayward, P. Haywood, H. Haywood Hasford, T. Hazelton, P. Hazenberg, Z. He, E. Headon, C. Heal, B. Healy, J.L. Healy, A. Hearn, D. Heasman, A. Heath, D. Heath, R. Heath, D. Heaton, A. Heavens, K. Hebbron, C. Heckman, G. Hector, S. Heddon, A. Hedges, K. Hedges, C. Heeley, E. Heeney, R. Heinink, R. Heire, I. Helgesen, J. Hemingway, U. Hemmila, B. Hemmings, S. Hemphill, D. Hemsley, A. Henderson, E. Henderson, J. Henderson, S. Henderson, J. Henry, K. Henry, L. Henry, M. Henry, N. Henry, D. Henshall, G. Herdman, R. Herdman-Grant, M. Herkes, L.E. Hermans, F. Hernandez, E. Heron, L. Heron, W. Herrington, E. Heselden, P. Heslop, T. Heslop, S. Hester, E. Hetherington, J. Hetherington, C. Hettiarachchi, P. Hettiarachchi, H. Hewer, J. Hewertson, A. Hewetson, S. Hewins, N. Hewitson, C. Hewitt, D. Hewitt, R. Hewitt, S. Hey, R.S. Heyderman, M. Heydtmann, J. Heys, J. Heywood, M. Hibbert, J. Hickey, N. Hickey, P. Hickey, N. Hickman, A. Hicks, J. Hicks, S. Hicks, P. Hien, T. Hien, T.T. Hien, D. Higbee, L. Higgins, A. Higham, M. Highcock, J. Highgate, M. Hikmat, A. Hill, H. Hill, J. Hill, L. Hill, P. Hill, U. Hill, A. Hilldrith, C. Hillman-Cooper, J. Hilton, Z. Hilton, S. Hinch, A. Hindle, E. Hindley, A. Hindmarsh, P. Hine, K. Hinshaw, C. Hird, C. Hirst, L. Hirst, J. Hives, H.M. Hlaing, B. Ho, D.K.K. Ho, R. Ho, L. Hoa, L.N.M. Hoa, M. Hoare, D. Hobden, G. Hobden, M. Hobrok, S. Hobson, C. Hodge, S. Hodge, L. Hodgen, G. Hodgetts, H. Hodgkins, S. Hodgkinson, D. Hodgson, H. Hodgson, L. Hodgson, S. Hodgson, G. Hodkinson, K. Hodson, M. Hodson, A. Hogan, M. Hogben, L. Hogg, L. Hoggett, A. Holborow, C. Holbrook, R. Holbrook, C. Holden, J. Holden, M. Holden, S. Holden, T. Holder, N. Holdhof, H. Holdsworth, L. Holland, M. Holland, N. Holland, P. Holland, S. Holland, M.L. Hollands, E. Holliday, K. Holliday, M. Holliday, N. Holling, L. Hollos, N. Hollos, L. Holloway, S. Holloway, M. Hollowday, M. Hollyer, A. Holman, A. Holmes, M. Holmes, R. Holmes, K. Holroyd, B. Holroyd-Hind, L. Holt, S. Holt, A. Holyome, M. Home, R. Homewood, K. Hong, L. Hoole, C. Hooper, S. Hope, B. Hopkins, P.W. Horby, S. Horler, A. Hormis, D. Hornan, N. Hornby, T. Horne, Z. Horne, R. Horner, C. Horrobin, L. Horsford, M. Horsford, V. Horsham, A. Horsley, E. Horsley, S. Horton, J. Hosea, T. Hoskins, M.S. Hossain, R. Hossain, M. Hough, S. Hough, C. Houghton, K. Houghton, R. Houlihan, K. Housely, H. Houston, R. Hovvels, L. How, L. Howaniec, J. Howard, K. Howard, L. Howard, M. Howard, S. Howard, R. Howard-Griffin, L. Howard-Sandy, S. Howe, A. Howell, M. Howells, L. Howie, K. Howlett, S. Howlett, R. Howman, J. Hrycaiczuk, H. Htet, N.Z. Htoon, S. Htwe, Y. Hu, C.O.H. Huah, S. Huang, K. Hubbard, A. Huckle, S. Huda, A. Hudak, L. Hudig, H. Hudson, S. Hudson, O. Hudson, A. Hufton, C. Huggins, A. Hughes, C. Hughes, E. Hughes, G. Hughes, H. Hughes, L. Hughes, M. Hughes, R. Hughes, S. Hughes, V. Hughes, W. Hughes, L. Huhn, C. Hui, R. Hulbert, D. Hull, G. Hull, R. Hull, A. Hulme, P. Hulme, W. Hulse, G. Hulston, R. Hum, M. Hume, C. Humphrey, A. Humphries, J. Humphries, T. Hung, C. Hunt, F. Hunt, J. Hunt, K. Hunt, L. Hunt, M. Hunt, S. Hunt, A. Hunter, C. Hunter, E. Hunter, K. Hunter, N. Hunter, R. Hunter, S. Hunter, G. Huntington, F. Huq, E. Hurditch, J. Hurdman, C. Hurley, K. Hurley, M.A. Husain, S. Husaini, C. Huson, A. Hussain, C. Hussain, I. Hussain, M. Hussain, R. Hussain, S. Hussain, Y. Hussain, M. Hussam El-Din, S.F.E.M. Hussein, Z. Hussein, R. Hussey, A.H. Hussien, A. Hutchinson, C. Hutchinson, D. Hutchinson, E. Hutchinson, J. Hutchinson, C. Hutsby, P. Hutton, N. Huy, N.Q. Huy, N. Huyen, N.T.T. Huyen, T. Huyen, T.B. Huyen, N.T. Huyen Thuong, H. Huynh, D. Hydes, J. Hyde-Wyatt, N. Hynes, M. Hyslop, A. Iakovou, K. Ibison, M. Ibraheim, A. Ibrahim, J. Ibrahim, M. Ibrahim, W. Ibrahim, B. Icke, A.I. Idowu, M. Idrees, N. Idrees, H. Iftikhar, M. Iftikhar, C. Igwe, O. Igwe, M. Ijaz, A. Ikomi, C. Iles, S. Iliodromiti, M. Ilsley, L. Ilves, F.M. Ilyas, L. Imam-Gutierrez, M. Iman, C. Imray, H. Imtiaz, J. Ingham, R. Ingham, T. Ingle, J. Inglis, S. Inglis-Hawkes, A. Ingram, L. Ingram, T. Ingram, N. Innes, P. Inns, V. Inpadhas, K. Inweregbu, A.A. Ionescu, A. Ionita, I. Iordanov, A. Ipe, J. Iqbal, M. Iqbal, F. Iqbal Sait, I. Irabor, J. Irisari, R. Irons, M. Irshad, M.S. Irshad, J. Irvine, V. Irvine, R. Irving, M. Ishak, E. Isherwood, G. Isitt, A. Islam, M.D.T. Islam, S. Islam, A. Ismail, O. Ismail, C. Ison, M. Israa, S. Isralls, H. Istiqomah, M. Ivan, C. Ivenso, N. Ivin, A. Ivy, S. Iwanikiw, K. Ixer, M. Iyer, A. Iakovou, K. Ibison, M. Ibraheim, A. Ibrahim, J. Ibrahim, M. Ibrahim, W. Ibrahim, B. Icke, A.I. Idowu, M. Idrees, N. Idrees, H. Iftikhar, M. Iftikhar, C. Igwe, O. Igwe, M. Ijaz, A. Ikomi, C. Iles, S. Iliodromiti, M. Ilsley, L. Ilves, F.M. Ilyas, L. Imam-Gutierrez, M. Iman, C. Imray, H. Imtiaz, J. Ingham, R. Ingham, T. Ingle, J. Inglis, S. Inglis-Hawkes, A. Ingram, L. Ingram, T. Ingram, N. Innes, P. Inns, V. Inpadhas, K. Inweregbu, A.A. Ionescu, A. Ionita, I. Iordanov, A. Ipe, J. Iqbal, M. Iqbal, F. Iqbal Sait, I. Irabor, J. Irisari, R. Irons, M. Irshad, M.S. Irshad, J. Irvine, V. Irvine, R. Irving, M. Ishak, E. Isherwood, G. Isitt, A. Islam, M.D.T. Islam, S. Islam, A. Ismail, O. Ismail, C. Ison, M. Israa, S. Isralls, H. Istiqomah, M. Ivan, C. Ivenso, N. Ivin, A. Ivy, S. Iwanikiw, K. Ixer, M. Iyer, K. Jabbar, C. Jack, J. Jackman, S. Jackman, A. Jackson, B. Jackson, E. Jackson, H. Jackson, L. Jackson, M. Jackson, N. Jackson, S. Jackson, Y. Jackson, J. Jacob, P. Jacob, R. Jacob, N. Jacques, H. Jadhav, A. Jafar, D. Jafferji, A. Jaffery, C. Jagadish, V. Jagannathan, A. Jagne, M. Jagpal, N. Jain, S. Jain, S. Jaiswal, D. Jajbhay, T. Jaki, P. Jali, B. Jallow, Y. Jaly, R. Jama, A. Jamal, S. Jamal, Z. Jamal, Y. Jameel, A. James, C. James, K. James, L. James, M. James, N. James, O. James, P. James, R. James, S. James, T. James, J. Jameson, L. Jamieson, A. Jamison, P. Jane, K. Janes, A. Janmohamed, D. Japp, P. Jaques, V. Jardim, C. Jardine, C. Jarman, E. Jarnell, E. Jarvie, C. Jarvis, R. Jarvis, P. Jastrzebska, H. Javed, A. Javier, M. Jawad, L. Jawaheer, A. Jayachandran, D. Jayachandran, A. Jayadev, A. Jayakumar, D. Jayaram, R. Jayaram, G. Jayasekera, T. Jayatilleke, A. Jayebalan, J. Jeater, S. Jeddi, V. Jeebun, M.S. Jeelani, K. Jeffery, H. Jeffrey, R. Jeffrey, N. Jeffreys, B. Jeffs, C. Jeffs, J.P. Jeganathan Ponraj, D. Jegede, T. Jemima, I. Jenkin, A. Jenkins, C. Jenkins, D. Jenkins, E. Jenkins, I. Jenkins, P. Jenkins, S. Jenkins, F. Jennings, J. Jennings, L. Jennings, V. Jennings, E. Jerome, D. Jerry, G. Jervis, E. Jessup-Dunton, J. Jesus Silva, C. Jetha, K. Jethwa, R. Jha, S. Jhanji, K. Jian, Z. Jiao, L. Jimenez, A. Jimenez Gil, J. Jith, T. Joefield, N. Johal, S. Johal, K. Johannessen, A. Johari, A. John, M. John, N. John, E. Johns, M. Johns, A. Johnson, E. Johnson, G. Johnson, K. Johnson, L. Johnson, M. Johnson, N. Johnson, O. Johnson, R. Johnson, B. Johnston, C. Johnston, J. Johnston, S. Johnston, V. Johnston, D. Johnstone, E. Johnstone, J. Johnstone, M. Joishy, A. Jones, B. Jones, C. Jones, C.E. Jones, D. Jones, E. Jones, G. Jones, J. Jones, K. Jones, K.E. Jones, L. Jones, L.M. Jones, M. Jones, N. Jones, O. Jones, P. Jones, P.H. Jones, R. Jones, R.E. Jones, S. Jones, T. Jones, R. Jonnalagadda, R. Jordache, M. Jordan, S. Jordan, A. Jose, L. Jose, S. Jose, A. Joseph, G. Joseph, P.A. Joseph, R. Joseph, S. Joseph, D. Joshi, M. Joshi, P. Joshi, T. Joshi, B. Josiah, D.K. Joy, L. Joy, T. Joyce, H. Ju, A. Ju Wen Kwek, A. Judd, E. Jude, P. Judge, J. Juhl, S. Jujjavarapu, M. Juniper, E. Juszczak, D. Jyothish, K. Jabbar, C. Jack, J. Jackman, S. Jackman, A. Jackson, B. Jackson, E. Jackson, H. Jackson, L. Jackson, M. Jackson, N. Jackson, S. Jackson, Y. Jackson, J. Jacob, P. Jacob, R. Jacob, N. Jacques, A. Jafar, D. Jafferji, A. Jaffery, C. Jagadish, V. Jagannathan, A. Jagne, M. Jagpal, N. Jain, S. Jain, R. Jaiswal, S. Jaiswal, D. Jajbhay, T. Jaki, B. Jallow, Y. Jaly, R. Jama, A. Jamal, S. Jamal, Z. Jamal, Y. Jameel, A. James, C. James, K. James, L. James, M. James, N. James, O. James, P. James, R. James, S. James, T. James, J. Jameson, L. Jamieson, A. Jamison, P. Jane, K. Janes, A. Janmohamed, D. Japp, P. Jaques, V. Jardim, C. Jardine, C. Jarman, E. Jarnell, E. Jarvie, C. Jarvis, R. Jarvis, P. Jastrzebska, H. Javed, A. Javier, M. Jawad, L. Jawaheer, A. Jayachandran, D. Jayachandran, A. Jayadev, A. Jayakumar, D. Jayaram, R. Jayaram, G. Jayasekera, T. Jayatilleke, A. Jayebalan, J. Jeater, S. Jeddi, V. Jeebun, M.S. Jeelani, K. Jeffery, H. Jeffrey, R. Jeffrey, N. Jeffreys, B. Jeffs, C. Jeffs, J.P. Jeganathan Ponraj, D. Jegede, T. Jemima, I. Jenkin, A. Jenkins, C. Jenkins, D. Jenkins, E. Jenkins, I. Jenkins, P. Jenkins, S. Jenkins, F. Jennings, J. Jennings, L. Jennings, V. Jennings, E. Jerome, D. Jerry, G. Jervis, E. Jessup-Dunton, J. Jesus Silva, C. Jetha, K. Jethwa, R. Jha, R.K. Jha, S. Jhanji, K. Jian, Z. Jiao, L. Jimenez, A. Jimenez Gil, J. Jith, T. Joefield, N. Johal, S. Johal, K. Johannessen, A. Johari, A. John, M. John, N. John, E. Johns, M. Johns, A. Johnson, E. Johnson, G. Johnson, K. Johnson, L. Johnson, M. Johnson, N. Johnson, O. Johnson, R. Johnson, B. Johnston, C. Johnston, J. Johnston, S. Johnston, V. Johnston, D. Johnstone, E. Johnstone, J. Johnstone, M. Joishy, A. Jones, B. Jones, C. Jones, C.E. Jones, D. Jones, E. Jones, G. Jones, J. Jones, K. Jones, K.E. Jones, L. Jones, L.M. Jones, M. Jones, N. Jones, O. Jones, P. Jones, P.H. Jones, R. Jones, R.E. Jones, S. Jones, T. Jones, R. Jonnalagadda, R. Jordache, M. Jordan, S. Jordan, A. Jose, L. Jose, S. Jose, A. Joseph, G. Joseph, P.A. Joseph, R. Joseph, S. Joseph, D. Joshi, M. Joshi, P. Joshi, T. Joshi, B. Josiah, L. Joy, T. Joyce, H. Ju, A. Ju Wen Kwek, A. Judd, E. Jude, P. Judge, J. Juhl, S. Jujjavarapu, M. Juniper, E. Juszczak, D. Jyothish, K. Kabiru Dawa, M. Kacar, D. Kadad, N. Kadam, N. Kader, A. Kailey, M. Kain, G. Kakoullis, A. Kakrani, A. Kala Bhushan, R.J.K. Kalayi, R. Kaliannan Periyasami, D. Kalita, I. Kalla, E. Kallistrou, T. Kalmus Eliasz, S. Kalsoom, E. Kam, J. Kamara, A. Kamath, P. Kamath, R. Kamath, S.A. Kamerkar, N. Kametas, M. Kamfose, C. Kamundi, D. Kanabar, L. Kane, S. Kanitkar, O. Kankam, T. Kannan, A. Kant, V. Kapil, R. Kapoor, S. Kapoor, S. Kaprapina, S. Kar, J. Kara, E. Karbasi, S. Karelia, R. Kark, A. Karkey, A. Karki, S. Karki, S. Karmali, V. Karunanithi, N. Karunaratne, N. Kasianczuk, A. Kasiappan Balasubramanian, V. Kasipandian, R. Kassam, J. Kathirgamachelvam, M. Kati, V. Katsande, K. Kaul, D. Kaur, D. Kaur, J. Kaur, S. Kaur, Z. Kausar, L. Kavanagh, S. Kavanagh, M.A.A. Kawser, A. Kay, J. Kay, R. Kay, S. Kay, J.N. Kayappurathu, S. Kayastha, C. Kaye, A. Kazeem, P. KC, M. Ke, T. Keady, R. Kearns, N. Kearsley, J. Keating, L. Keating, E. Keddie-Gray, B. Keegan, R. Keen, N. Keenan, J. Kefas, S. Kegg, L. Keith, U. Keke, J. Kellett, J. Kelliher, A. Kelly, D. Kelly, E. Kelly, H. Kelly, L. Kelly, M. Kelly, R. Kelly, S. Kelly, M. Kelly-Baxter, O. Kelsall, M. Keltos, T. Kemp, E. Kendall, A. Kendall-Smith, S. Kennard, A. Kennedy, J. Kennedy, M. Kennedy, S. Kennedy-Hay, J. Kenny, M. Kent, L. Keogan, A. Keough, D. Kernaghan, A. Kerr, C. Kerrison, A. Kerry, H. Kerslake, I. Kerslake, H. Kerss, J. Keshet-Price, E. Kestelyn, G. Keyte, A. Khadar, D. Khadka, N.V. Khai, P. Khairunnisa, A. Khalid, H. Khalid, M. Khalid, M.U. Khalid, S. Khalid, T. Khalifa, A. Khalil, S. Khalil, A. Khan, B. Khan, F. Khan, M. Khan, K. Khan, M. Khan, M.A. Khan, N. Khan, O. Khan, R. Khan, S. Khan, T. Khan, W. Khan, Z. Khan, M.S. Khan Tharin, N.H. Khanh, U. Khatana, J. Khatri, H. Khatun, T. Khatun, M. Kheia, J. Khera, D. Khiem, D.P. Khiem, H.H.E. Khin, T.D. Khoa, N. Khoja, K. Khokhar, M.Q. Khong, J. Khoo, V.T. Khuong, C. Khurana, J. Kibaru, F. Kibutu, A. Kidd, M. Kidd, J. Kidney, S. Kidney, W. Kieffer, T. Kien, T.V. Kien, J. Kilbane, C. Kilby, E. Killen, B. Kilner, S. Kilroy, B. Kim, J.W. Kim, M. Kim, A. Kimber, S. Kimber, A. King, B. King, H. King, J. King, K. King, M. King, R. King, S. King, V. King, E. King-Oakley, L. Kingsmore, D.J. Kinnear, F. Kinney, S. Kiran, A. Kirby, A. Kirk, J. Kirk, A. Kirkby, E. Kirkham, G. Kirkman, L. Kirkpatrick, U. Kirwan, T. Kitching, L. Kitto, L. Kittridge, T. Kjoa, S. Klaczek, F. Kleemann, S. Kmachia, V. KN, C.P. Knapp, L. Knibbs, A. Knight, F. Knight, M. Knight, S. Knight, T. Knight, E. Knights, J. Knights, M. Knolle, P. Knopp, C. Knowles, K. Knowles, L. Knowles, E. Knox, L. Knox, O. Koch, M. Kocsor, R. Kodituwakku, G. Koduri, Y.J. Koe, J. Koirala, A. Koirata, E. Kolakaluri, M. Kolodziej, E. Kolokouri, S. Kon, N. Konar, M. Kononen, A. Konstantinidis, R. Kontogonis, H. Koo, I. Koopmans, E. Kopyj, L. Korcierz, J. Korolewicz, G. Koshy, C. Kosmidis, C. Kosztolanyi, J. Kotecha, E. Kothandaraman, R. Kothavale, K. Koukou, A. Kountourgioti, K. Kouranloo, R. Kousar, M. Kousteni, A. Koutalopoula, M. Kovac, A. Kozak Eskenazia, K. Krasauskas, R. Krishnamurthy, V. Krishnamurthy, M. Krishnan, H. Krishnan, N. Krishnapalli, S. Krizak, S. Krueper, S. Krupej, J. Krzowski, R. Kubaisi, S. Kubheka, A. Kubisz-Pudelko, S. Kuckreja, S. Kudsk-Iversen, A. Kudzinskas, C. Kukadiya, N. Kulkarni, S. Kumala Dewi, M. Kuma-Mintah, A. Kumar, G. Kumar, M. Kumar, R. Kumar, S. Kumar, V. Kumar, P. Kumar Panda, A. Kundu, H. Kunst, S.S. Kunwar, K. Kupiec, A. Kurani, M. Kurdy, K. Kuriakose, R. Kurian, V. Kurmars, C. Kuronen-Stewart, R.S. Kusangaya, V. Kushakovsky, A. Kutera, A. Kuverji, A. Kyei-Mensah, H. Kyepa, T. Kyere-Diabour, M. Kyi, N.M. Kyi, L. Kyle, K.-T. Kyriaki, J. Labao, L. Labuschagne, L. Lacey, N. Lack, M. Lacson, Z. Ladan, E. Ladlow, H. Lafferty, A. Lagnado, S. Laha, S. Lahane, C. Lai, J. Lai, P. Laidler, R. Laing, I. Laing-Faiers, E. Laity, K. Lake, N. Lakeman, D. Lalloo, F. Lalloo, A. Lam, C. Lam, F. Lamb, L. Lamb, T. Lamb, O. Lambert, P. Lambert, C. Lameirinhas, M.K.G. Lami, H. Lamont, M. Lamparski, D. Lamrani, C. Lanaghan, I. Lancona-Malcolm, G. Landers, M.J. Landray, M. Lane, N. Lane, A. Lang, S. Lang, D. Langer, M. Langley, C. Langoya, E. Langridge, E. Langthorne, H. Langton, B. Lara, T. Large, L.N. Lartey, S. Lassa, A. Last, S. Latham, V. Latham, A. Latheef, L. Latif, N. Latt, C. Lau, D. Lau, E. Lau, G.G. Laura, M. Laurenson, E. Lavington, H. Law, J. Law, K.Y. Law, P. Law, R. Law, L. Lawless, C. Lawrence, E. Lawrence, G. Lawrence, H.M. Lawrence, N. Lawrence, R. Lawrie, L. Lawson, N. Lawson, R. Lawson, M. Lay, S. Laybourne, C. Laycock, R. Layug, M. Lazo, D.H. Le, T.T. Le, V. Le, A. Lea, W. Lea, L.E. Leach, I. Leadbitter, T. Leahy, R. Lean, L. Leandro, D. Leaning, R. Leary, S. Leason, M.A. Ledingham, C. Lee, E. Lee, G. Lee, H. Lee, I. Lee, J. Lee, S. Lee, S.H. Lee, T. Lee, X. Lee, R. Lee, D. Lees, J. Lees, H. Legge, J. Leggett, K. Leigh-Ellis, D. Leitch, N. Leitch, E. Lekoudis, P. Lemessy, N. Lemoine, R. Lenagh, K. Leng, K. Lennon, L. Lennon, B. Leonard, K. Leonard, W. Leong, N. Leopold, O. Lepiarczyk, I. Leslie, E.N. Lestari, E. Lester, E. Levell, C. Levett, A. Levynska, A. Lewin, A. Lewis, C. Lewis, D. Lewis, H. Lewis, J. Lewis, K. Lewis, L. Lewis, M. Lewis, N. Lewis, R. Lewis, C. Lewis-Clarke, A. Lewszuk, P. Lewthwaite, S. Ley, A. Liao, V. Licence, D. Lieberman, S. Liebeschuetz, T. Light, N. Lightfoot, P. Lillie, A. Lillis, B. Lim, C. Lim, E.T. Lim, I. Lim, T. Lim, W. Lim, W.S. Lim, J. Limb, D. Limbu, U. Limbu, C. Linares, D. Linden, G. Lindergard, K. Lindley, C. Lindsay, E. Lindsay, M. Lindsay, H. Lindsay-Clarke, M. Ling, C. Lingam, N.V.H. Linh, V.D. Linh, L. Linkson, T. Linn, M. Linney, C. Lippold, G. Lipscomb, K. Lipscomb, L. Lipskis, A. Lisboa, E. Lister, J. Little, S. Little, L. Littlejohn, S. Liu, X. Liu, D.K. Llanera, R. Llewellyn, M. Llewelyn, A. Lloyd, O. Lloyd, R. Lloyd, S. Lo, D. Loader, C. Loan, L. Lobosco, L. Lock, S. Lock, A. Locke, J. Locke, T. Locke, T. Lockett, J. Lodge, K. Lodhia, M. Lofthouse, H. Loftus, M. Logan, C. Logue, S.Y. Loh, S. Lokanathan, K. Lomme, E. London, G. Long, N. Long, K. Longbottom, B. Longhurst, M. Longshaw, S. Longstaffe, J. Lonnen, C. Lonsdale, L. Looby, R. Loosley, L. Lopes, P. Lopez, P. Lopez, R.W. Lord, S. Lord, C. Lorimer, F. Loro, R. Lorusso, C. Loughlin, W. Lovegrove, R. Loveless, M. Lovell, A. Loverdou, A. Low, J. Low, S. Low, A. Lowe, C. Lowe, E. Lowe, F. Lowe, L. Lowe, M. Lowe, R. Lowsby, V. Lowthorpe, G. Lubimbi, A. Lubina Solomon, G. Lucas, J. Lucas, A. Lucey, O. Lucey, S. Luck, L.H. Lui, A. Luintel, H. Luke, J. Luke, N. Lungu, A. Lunia, M. Lunn, J. Luo, M. Luscombe, J. Luveta, C.N. Luximon, K. Lwin, M. Lwin, A. Lye, B. Lyell, E. Lyka, A. Lynas, C. Lynch, D. Lynch, S. Lynch, R.-G. Maamari, H. Mabb, L. Mabelin, G. Mabeza, J. Macaro, K. Macconaill, C. Macdonald, A. Macduff, C. Macfadyen, J.G. Macfarlane, J. Macfarlane, L. Macfarlane, I. Macharia, L. MacInnes, I. MacIntyre, J. MacIntyre, K. Mack, C. Mackay, E. Mackay, L. Mackay, A. Mackenzie, M. Mackenzie, R. MacKenzie Ross, A. Mackey, F. Mackie, J. Mackie, R. Mackie, C. Mackinlay, C. Mackintosh, K. Mackintosh, M.J. MacLeod, S. Macleod, M. Macmahon, A. MacNair, C. Macphee, I. Macpherson, C. Macrae, A. MacRaild, Y. Madani, A. Madden, M. Madden, C. Madden-McKee, S. Maddison, N. Madeja, P. Madhivathanan, M. Madhusudhana, A. Madu, L. Madziva, M. Mafham, S. Magar, N. Magee, F. Magezi, N. Maghsoodi, C. Magier, L.M. Magnaye, M. Magriplis, M. Magtalas, N.P. Magula, N. Mahabir, S. Mahadevan-Bava, S. Mahajan, A. Maharajh, K. Maharjan, M. Maharjan, A. Mahaveer, B. Mahay, K. Mahay, A. Mahdi, H. Mahdi, N. Mahdi, T. Mahendiran, S. Mahendran, S. Maher, A. Maheswaran, S. Maheswaran, T. Maheswaran, P. Mahjoob-Afag, A. Mahmood, F. Mahmood, H. Mahmood, W. Mahmood, Z. Mahmood, H. Mahmoud, M. Mahmud, E. Mahony, T. Mahungu, O. Maiga, L. Mair, T. Majekdunmi, K. Majid, A. Major, R. Major, J. Majumdar, M.K.H. Majumder, T.L.A. Mak, A. Makan, E. Makanju, S. Makin, W.-O. Makinde, Y. Makkeyah, O.N. Makoetlane, M. Malanca, H. Malcolm, F. Malein, N. Malhan, A. Malicka, A. Malik, G. Malik, M. Maljk, P. Mallett, P. Mallinder, G. Mallison, L. Mallon, E. Malone, G. Maloney, M. Mamman, I. Man, K. Man, R. Mancinelli, M. Mancuso-Marcello, S. Mandal, S.K. Mandal, T. Manders, L. Manderson, J. Mandeville, T. Mane, R. Manhas, C. Maniero, R. Manikonda, I. Manjra, R. Mankiewitz, B. Mann, J. Manning, S. Manning, P. Mannion, K. Mansi, K. Manso, D. Mansour, M. Mansour, R. Mansour, I.T. Mapfunde, P. Mappa, A. Maqsood, H. Maraj, C. Marchand, N. Marcus, A. Marcyniuk, M. Marecka, D. Maren, G. Margabanthu, J. Margalef, L. Margarit, G. Margaritopoulos, M. Margarson, F. Maria del Rocio, T. Maria Pfyl, V. Mariano, A. Maric, G. Markham, B. Marks, M. Marks, P. Marks, E. Marler, E. Marouzet, A. Marriott, C. Marriott, N. Marriott, C. Marsden, K. Marsden, P. Marsden, S. Marsden, T. Marsden, C. Marsh, G. Marsh, R. Marsh, A. Marshall, A. Marshall, G. Marshall, H. Marshall, J. Marshall, J. Marshall, N. Marshall, R. Marshall, S. Marshall, J. Marshall, E. Martin, G. Martin, H. Martin, J. Martin, K. Martin, L. Martin, M. Martin, N. Martin, T. Martin, W. Martin, S. Martin, T. Martindale, M. Martineau, L. Martinez, J.C. Martinez Garrido, J. Martin-Lazaro, V.K. Maruthamuthu, B. Marwan, G. Maryan, R. Mary-Genetu, S. Maryosh, V. Masani, A. Mascagni, D. Maseda, Z. Maseko, S. Mashate, Y. Mashhoudi, A. Mashta, I. Masih, S. Masih, N. Maskell, P. Maskell, P. Maskey, M. Masoli, J. Mason, R. Mason, C. Mason, M. Masood, M.T. Masood, S.S.M.E. Masood, T. Massa, I. Massey, J. Masters, A. Masud, L. Matapure, C. Matei, R. Matewe, E. Matey, M. Matharu, S. Mathen, A. Mather, N. Mather, J. Mathers, J. Matheson, A. Mathew, A. Mathew, M. Mathew, V. Mathew, J. Mathews, K. Mathias, A. Mathioudakis, S. Matibela, D. Matila, W. Matimba-Mupaya, N. Matin, E. Matisa, E. Matkins, M. Matonhodze, E. Matovu, J. Mattappillil, A.J. Matthews, C. Matthews, H. Matthews, L. Mattocks, C. Maughan, T.T. Maulidya, E. Mawson, F. Maxton, A. Maxwell, V. Maxwell, E. May, J. May, P. May, I. Mayanagao, M. Maycock, J. Mayer, G. Mayers, V.A. Maynard, K. Mayne, T. Mayo, L. Mayola, S. Mayor, I. Mazen, T. Mazhani, A. Mazzella, N. Mburu, A. Mbuyisha, C. Mc Cague, E. McAleese, P. McAlinden, L. McAllister, A. McAlpine, G. McAlpine, J. McAndrew, H. McAuley, S. McAuliffe, C. McBrearty, E. McBride, M. McBuigan, J. McBurney, L. McCabe, G.L. McCafferty, L. McCafferty, A. McCairn, J. McCammon, N. McCammon, C. McCann, E. McCann, A. McCarrick, B. McCarron, E. McCarthy, M. McCarthy, N. McCarthy, S. McCaughey, T. McClay, B. McClelland, D. McClintock, M. McCloskey, K. McCollum, A. McCorkindale, P. McCormack, J. McCormick, W. McCormick, P. McCourt, J. McCrae, S. McCready, G. McCreath, H. McCreedy, C. McCue, I.J. McCullagh, L. McCullagh, M. McCullagh, C. McCullough, K. McCullough, N. McCullough, S. McCullough, F. McCurrach, J. McDermott, P. McDermott, R. McDermott, K. McDevitt, H. McDill, B. McDonald, C. McDonald, D. McDonald, R. McDonald, S. McDonald, D. McDonald, N. McDonnell, C. McDougall, L. McDougall, R. McDougall, I. McEleavy, F. McElwaine, J. McEntee, E. McEvoy, C. McEwan, R. McEwen, M. McFadden, D. McFarland, M. McFarland, R. McFarland, J. McFlynn, E. McGarry, L. McGarvey, A. McGeachan, F. McGee, L. McGenily, C. McGettigan, M. McGettrick, C. McGhee, F. McGill, S. McGinnity, N. McGlinchey, P. McGlone, D. McGlynn, C. McGoldrick, E. McGough, C. McGovern, R. McGovern, A. McGowan, A. McGown, B. McGrath, A. McGregor, M.P. McGuigan, H. McGuinness, S. McGuire, T. McHugh, C. McInnes, N. McInnes, J. McIntosh, K. McIntyre, M. McIntyre, L. McKay, C.P. McKeag, J. McKeane, M. McKee, J. McKeever, J. McKenna, S. McKenna, M. McKenzie, D. McKeogh, C. McKerr, A.M. McKie, H. Mckie, L. Mckie, G. McKnight, H. McLachlan, A. McLaren, B. McLaren, N. McLarty, D. Mclaughlan, M. McLaughlin, J. McLay, M. McLeish, T. McLennan, S. McLure, A.M. McMahon, G. McMahon, M. McMahon, S. McMahon, T. McManus, M. McMaster, P. McMaster, P. McMaster, F. Mcmeeken, S. McMeekin, N. McMillan, K. McMillen, J. McMinn, L. McMorrow, H. McMullen, C. McMurran, H. McNally, F. McNeela, L. McNeil, C. McNeill, J. McNeill, S. McNeill, U. McNelis, M. McNulty, R. McNulty, C. McParland, M. McPhail, A. McQueen, A. McSkeane, D. McSorland, T. McSorley, G. McTaggart, J. McTaggart, J. Mead, P. Mead, E. Meadows, O. Meakin, B. Mearns, C. Mearns, K. Mears, W. Mears, M. Meda, A. Mediana, R. Medine, T. Medveczky, S. Meehan, E. Meeks, A. Megan, N. Meghani, S. Meghjee, S. Megson, A. Mehar, M.N. Mehmood, R. Mehra, R. Mehta, G. Meintjes, J. Meirill, J. Meiring, R. Mejri, E. Mekonnen, S. Melander, A.-S. Melinte, J. Mellersh, L. Melling, C. Mellish, F. Mellor, J. Mellor, S. Mellor, Z. Mellor, K. Mellows, V. Melnic, A. Melville, D. Melville, J. Melville, H. Membrey, M. Mencias, A. Mendelski, M. Mendelson, C. Mendonca, C. Meney, C. Menezes, W. Mensah, J.E. Mensshan, A. Mentzer, D. Menzies, S. Menzies, S. Mepham, O. Mercer, P. Mercer, A. Merchant, F. Merchant, M. Mercioniu, M. Meredith, M. Merida Morillas, B. Merrick, J. Merritt, S. Merritt, P. Merron, E. Merwaha, S. Message, J. Messenger, G. Metcalf-Cuenca, A. Metcalfe, B. Metcalfe, K. Metcalfe, S. Metherell, A. Metryka, L. Mew, S. Meyrick, N. Mguni, J. Mhlongo, A. Miah, J. Miah, N. Miah, A. Mian, G. Mic, L. Micah-Amuah, D. Micallef, A. Michael, S. Michael, N. Michalak, L. Michalca-Mason, O. Michalec, J. Middle, H. Middleton, J. Middleton, M. Middleton, S. Middleton, S. Mieres, L. Mihalca-Mason, T. Mikolasch, S. Milgate, C. Millar, J. Millar, J. Millard, D. Miller, J. Miller, L. Miller, R. Miller, N. Miller-Biot, A. Miller-Fik, L. Millett, B. Milligan, H. Milligan, I. Milligan, C. Milliken, K. Millington, R. Millington, S. Millington, H. Mills, J. Mills, R. Mills, H. Millward, R. Miln, A. Milne, C. Milne, L. Milne, J. Milner, L. Milner, Z. Min, S. Mindel, N. Minh, P.A. Minkah, C. Minnis, P. Minnis, K. Minou, N. Minskip, J. Minton, F. Miranda, M. Mirela, T. Mirza, A. Misbahuddin, A. Mishra, B. Mishra, E. Mishra, R. Mishra, S. Misra, D. Mistry, H. Mistry, D. Mital, S. Mitchard, B. Mitchell, C. Mitchell, L.J. Mitchell, P. Mitchell, P. Mitchelmore, A. Mitra, A. Mitra, S. Mitra, N. Mlambo, E. Moakes, K. Moar, E. Moatt, D. Mock Font, G. Modgil, A. Mohamed, A. Mohamed, O. Mohamed, A. Mohammad, W. Mohammad, A. Mohammed, O. Mohammed, Y.N.S. Mohammed, B. Mohamud, A. Moharram, H.-P. Mok, J. Mok, L. Mokogwu, M. Molina, C. Moller-Christensen, M. Mollet, M. Molloholli, A. Molloy, L. Molloy, A. Molyneux, R. Molyneux, T. Momoniat, H. Monaghan, K. Monaghan, S. Mongolu, T. Monika, K. Monsell, M. Montasser, A. Montgomery, H. Montgomery, P. Moodley, M. Moody, N. Moody, A. Moon, J. Moon, J.-H. Moon, M. Moon, M. Moonan, P. Moondi, S. Moorby, J. Moorcroft, A. Moore, C. Moore, D.A.J. Moore, F. Moore, J. Moore, L. Moore, N. Moore, S. Moore, V. Moore, R. Moores, E. Morab, J. Morales, N. Moramorell, L. Moran, G. Moray, J. Moreno-Cuesta, A. Morgan, C. Morgan, H. Morgan, K. Morgan, L. Morgan, M. Morgan, P. Morgan, K. Morgan-Jones, E. Morgan-Smith, J. Morilla, A. Morley, T. Morley, W. Morley, A. Morris, D. Morris, F. Morris, H. Morris, J. Morris, K. Morris, L. Morris, M.-A. Morris, N. Morris, P. Morris, S. Morris, D. Morrison, M. Morrison, S. Morrison, M. Morrissey, A.C. Morrow, A. Morrow, F. Morselli, G. Mortem, V. Mortland, C. Morton, G. Morton, P. Morzaria, D. Mosby, L. Moseley, K. Moshal, B. Moshy, A. Moss, C. Moss, J. Moss, S. Moss, O. Mostafa, G. Moth, N. Motherwell, S. Mottershaw, H. Moudgil, J. Mouland, C. Moulds, H. Moulton, G. Mounce, E. Mousley, C. Mowatt, K. Moxham, B. Moya, Q. Moyo, E. Mshengu, S. Mtuwa, A. Muazzam, I.A. Muazzam, N. Muchenje, D. Mudawi, G. Muddegowda, R. Mufti, I. Mugal, A. Mughal, J. Muglu, F. Muhammad, J. Muhammad, C. Muir, A. Mukherjee, D. Mukherjee, J. Mukhtar, S.A.A. Mukhtar, D. Mukimbiri, J. Mulcahy, M. Mulcahy, P. Mulgrew, B. Mulhearn, A. Mulla, D. Mullan, D. Mullasseril Kutten, N. Mullen, R. Mullett, C. Mulligan, S. Mulligan, L. Mumelj, A. Mumford, M. Munavvar, H. Munby, H. Munday, A. Munro, S. Munt, M. Mupudzi, A. Murad, O.H. Muraina, K. Muralidhara, M. Murdoch, J. Murira, A. Murphy, B. Murphy, C. Murphy, E. Murphy, G. Murphy, H. Murphy, P. Murphy, R. Murphy, S. Murphy, C. Murray, D. Murray, E. Murray, K. Murray, L. Murray, T. Murray, E. Murtagh, M. Murthy, C. Murton, R. Murton, N. Muru, R. Musanhu, M. Mushabe, O. Mushtaq, S. Musini, A.M.M. Mustafa, E. Mustafa, M. Mustafa, I. Mustapha, N. Mustfa, Z. Mustufvi, C. Mutch, R. Mutch, E. Mutema, B. Muthukrishnan, S. Mutton, N. Muzengi, M. Mwadeyi, B. Mwale, E. Mwaura, R. Myagerimath, A. Myers, S. Myers, J.S. Myerson, K. Myint, Y. Myint, G. Mynott, L. Myslivecek, P. Nabayego, E. Nadar, I. Nadeem, M. Nadheem, B. Nadjm, A. Naeem, H. Naeem, S. Naeem, S. Nafees, M. Nafei, W. Naftalia, T. Nagarajan, L. Naglik, I. Nagra, D. Nagra, M. Naguib, K. Naguleswaran, K.S. Nagumantry, K. Naicker, S. Naidoo, V. Naidoo, G. Naik, R. Naik, S. Naik, D.S. Nair, R. Nair, T. Nair, J. Naisbitt, K. Naismith, D. Nakiboneka-Ssenabulya, S. Nallapareddy, S. Nallapeta, A. Nallasivan, H. Nam, U. Nanda, A. Nandani, T. Nandwani, A.R. Naqvi, A. Naqvi, S. Naqvi, S. Nasa, D. Nash, N. Nasheed, A. Nasimudeen, U. Nasir, N. Nasronudin, T. Nasser, A. Natarajan, G. Natarajan, N. Natarajan, R. Natarajan, P. Nath, N. Nathaniel, M. Nathvani, P. Nathwani, G. Nava, N. Navaneetham, J. Navaratnam, H. Navarra, S. Naveed, J. Navin, K. Nawaz, S. Nawaz, B. Nayar, S. Naylor, M. Nayyar, F. Naz, M. Naz, B. Nazari, A. Nazir, S. Nazir, D. Ncomanzi, O. Ndefo, N.B. Ndoumbe, A. Neal, E. Neary, M. Negmeldin, J. Neil, P. Neill, H.E. Neils, A. Nejad, J. Nel, L. Nel, A. Nelson, B. Nelson, L. Nelson, M. Nelson, R. Nelson, S. Nelson, E. Nelwan, E.J. Nelwan, R. Nemane, S. Nepal, D. Nethercott, K. Netherton, K. Nettleton, J. Neupane, K. Neupane, A. Newby, D. Newby, T. Newcombe, H. Newell, C. Newman, D. Newman, H. Newman, J. Newman, O. Newman, T. Newman, R. Newport, M. Newton, A.Y.K.C. Ng, H.E.J. Ng, K.W. Ng, M. Ng, S. Ng, W.J. Ng, Y.W.M. Ng, T. Ngan, T.H. Ngo, G.C.E. Ngui, A. Ngumo, H.K. Nguyen, M.T. Nguyen, N. Nguyen, N.T. Nguyen, N.T.T. Nguyen, Q. Nguyen, T.H. Nguyen, T.H.T. Nguyen, T.T. Nguyen, T.T.N. Nguyen, T.T. Nguyen, T.T.P. Nguyen, K. Ngwenya, N.Y. Nhi, C. Nic Fhogartaigh, N. Nicholas, P. Nicholas, R. Nicholas, D. Nicholls, L. Nicholls, S. Nicholls, A. Nicholson, I. Nickson, E. Nicol, R. Nicol, P. Nicola, A. Nicoll, T. Nightingale, F. Nikita, P. Nikolaos, G. Nikonovich, A. Nilsson, K. Nimako, L. Nimako, C. Nimmo, P. Ninan, T. Ninh, M. Nirmalan, R. Niroula, A. Nisar, M. Nisar, T. Nisar, T. Nisbett, A. Nisha James, S. Nishat, T. Nishiyama, S. Nix, J. Nixon, M. Nixon, K. Nizam Ud Din, M. Nizami, S. Nizamis, R. Njafuh, I. Noakes, L. Noba, J. Noble, H. Noble, H.M. Noe, J. Nolan, J. Nolasco, Z. Noor, Z. Noori, J. Norcliffe, L. Norman, R. Norman, E. Norris, K. Norris, L. Norris, S.A. Nortcliffe, F. North, J. North, T. North, J. Northfield, S. Northover, J. Nortje, D. Norton, R. Norton, H. Notman, K. Nourein, T. Novak, N. Novas Duarte, C. Novis, J.A. Nowak, K.P. Nu, M. Nugdallah, A. Nugent, J. Nugent, C. Nugroho, N. Numbere, K. Nundlall, A. Nune, K. Nunn, M. Nunn, J. Nunnick, Y. Nupa, F. Nur, Z. Nurgat, R. Nurpeni, A. Nuttall, L. Nwafor, P. Nwajiugo, G. Nyamugunduru, L. Nyanor, M. Nyirenda, K. Nyland, D.O. Rinn, D.O. Shea, M. O Toole, M. O’Hara, C. O’Hara, L. O’Keefe, K. O’Reilly, W. O’Rourke, C. Oakley, N. Oakley, S. Oakley, H.T.K. Oanh, B. Obale, C. Oboh, C. O'Brien, J. O'Brien, K. O'Brien, L. O'Brien, N. O'Brien, R. O'Brien, T. O'Brien, E. O'Bryan, R. Obukofe, C. O'Callaghan, L. O'Connell, T. OConnor, C. O'Connor, G. O'Connor, M. Odam, S. Oddie, S. Oddy, Y. Odedina, K. Odedra, S. Odelberg, N. Odell, O. Oderinde, J. Odone, L. O'Donohoe, C. O'Donovan, I. Odysseos-Beaumont, S. O'Farrell, P. Offord, M. O'Flaherty, E. Ofori, T. Ogbara, C. Ogilvie, C. O'Gorman, I. Ogunjembola, O. Ogunkeye, U. Ohia, S. Ojha, S. Ojha, O. Ojo, F. O'kane, M. O'Kane, T. Okeke, E. OKell, A. Okines, I. Okpala, E. Okpo, F. Okpoko, M. Okubanjo, C. Oladipo, L. Olaivar, R. Olaiya, J. Olatujoye, T. Old, G. Oleszkiewicz, A. Oliver, C. Oliver, J. Oliver, L. Oliver, M. Oliver, Z. Oliver, J. Oliver-Commey, N.O. Olokoto, F. Olonipile, O. Olufuwa, O. Olukoya, A. Oluwole-Ojo, L. O'Malley, I.V. Omale, P.K. Omane-Donkor, M. Omar, Z. Omar, N. Omer, E. Omoregie, C. O'Neill, L. O'Neill, C. Ong, O. Onuoha, C. Onyeagor, C.N. Oo, Z. Oo, H.C. Ooi, S.H. Ooi, A. Oomatia, A. Opata, M. Opena, R. Oram, C. Ord, J. Ord, C. Oreilly, L. Orekoya, D. O'Riordan, S. O'Riordan, I. Orlikowska, A. Orme, H. Orme, L. O'Rourke, C. Orr, S. Orr, C. Orton, A. Osadcow, R. Osagie, R. Osanlou, L. Osborne, N. Osborne, R. Osborne, W. Osborne, W. Osborne, C. Osbourne, J. Osei-Bobie, J. Osman, W. Osman, B. Osman, G. Osoata, M. Ostermann, E. O'Sullivan, S. O'Sullivan, M.A. Oteng, N. Otey, O.K. Otite, M. O'Toole, J. Ouyang, R. Owen, S. Owen, E. Owens, C. Owoo, Y. Owoseni, M. Owston, R. Oxlade, F. Ozdes, J. Pack, A. Packham, S. Packham, P. Paczko, G. Padden, A. Padmakumar, C. Page, I. Page, J. Page, S. Page, V. Page, J. Paget, K. Pagett, V. Pai, L. Paisley, S. Pajak, G. Pakou, A. Pakozdi, S. Pal, S. Pal, A. Palacios, V.B. Palagiri Sai, V. Palaniappan, P. Palanivelu, A. Palfreeman, H. Palfrey, V. Palissery, D. Palit, S. Pallipparambil Antony, J. Palman, A. Palmer, H. Palmer, J. Palmer, L. Palmer, R. Palmer, A. Pambouka, I. Pamphlett, D. Pan, A. Pandey, N. Pandian, K. Pandya, T. Pandya, H.R. Paneru, A. Panes, J. Pang, Y.W. Pang, R. Pangeni, L. Pannell, K. Pannu, S. Pant, S. Panthakalam, C.T. Pantin, N. Pao, H. Papaconstantinou, N.S. Papavarnavas, P. Papineni, K. Paques, A.W. Paracha, K. Paradowski, V. Parambil, S. Paranamana, S.R. Parashar, I. Parberry, A. Parekh, D. Parekh, L. Parfitt, H. Parfrey, O. Parikh, G. Parish, J. Park, V. Parkash, A. Parker, B. Parker, E. Parker, H. Parker, J. Parker, L. Parker, N. Parker, S. Parker, K. Parkin, A. Parkinson, M. Parkinson, V. Parkinson, C. Parmar, V. Parmar, V. Parris, C. Parrish, B. Parry, H.C. Parry, S. Parslow-Williams, M. Parsonage, G. Parsons, J. Parsons, P. Parsons, R. Partridge, Z. Parvez, K. Parvin, L. Passby, S. Passey, H. Passmore, J. Pastrana, J. Patachako, M. Patal, S. Patch, A. Patel, A. Patel, B. Patel, D. Patel, H. Patel, J. Patel, K. Patel, M. Patel, N. Patel, P. Patel, S. Patel, T. Patel, Z. Patel, V. Patel, K. Paterson, S. Pathak, N. Pathan, A. Patience, D. Patience, B. Patil, R. Patmore, S. Patole, L. Paton, A. Patrick, G. Patrick, J. Patrick, S. Patten, B. Pattenden, C. Patterson, J. Patterson, L. Patterson, M. Patterson, R. Patterson, M. Pattrick, D. Paudel, K. Paudel, M. Paudel, S. Paudel, M. Paul, S. Paul, L. Pauls, S. Paulus, A. Pavely, M.J. Pavitt, S. Pavord, B. Payne, E. Payne, M. Payne, R. Payne, L. Peacock, S. Peacock, H. Peake, J. Pearce, R. Pearse, A. Pearson, D. Pearson, H. Pearson, K. Pearson, S. Pearson, S.A. Pearson, A. Peasley, H. Peddie, S. Peebles, R. Peek, A. Peer, S. Peerbhoy, C. Pegg, E. Peggie, H. Peggie, S. Peglar, B.H. Peirce, M. Peirse, C. Pelham, A. Pemberton, M. Penacerrada, A. Pender, C. Pendlebury, J. Pendlebury, R. Penfold, C. Penman, J. Penman, R. Penman, J. Penner, K. Penney, A. Pennington, J. Penny, J. Pepperell, R. Percival, A. Pereira, R. Pereira, C. Pereira Dias Alves, I. Perera, M. Perera, E. Perez, J. Perez, T. Perinpanathan, L. Periyasamy, E. Perkins, I. Pernicova, E. Perritt, A. Perry, E. Perry, M. Perry, T.M. Perumpral, G. Pessoa-Amorim, R. Petch, L. Peter, C. Peters, L. Peters, M. Peters, S. Peters, T. Peters, A. Peterson, R. Petersen, L. Peto, I. Petras, B. Petrova, M. Petrova, E. Petrovics, T. Pettigrew, M. Pezard-Snell, P. Pfeffer, G. Phalod, N.T. Pham, V.P. Pham, T.T.H. Phan, M. Phanish, P. Phelan, C. Philbey, J. Philbin, A. Phillips, B. Phillips, D. Phillips, N. Phillips, P. Phillips, R. Phillips, T. Phillips, M. Phipps, M. Phipps, N. Phong, N.T. Phong, V. Phongsathorn, P. Phuc, P.V. Phuc, M. Phull, H. Phung, H.T.K. Phung, H.M. Phuong, N. Phuong, A. Phuyal, A.K. Phyo, M.T.T. PI, S. Pick, J. Pickard, C. Pickering, F. Pickering, G. Pickering, T. Pickett, J. Pickles, S. Pickstock, B. Pickwell-Smith, N. Pieniazek, C. Piercy, A. Pieris, S. Pilgrim, P.A. Pillai, S. Pillay, L. Pilling, Z. Pilsworth, H. Pinches, S. Pinches, K. Pine, M.T. Pinjala, S. Pintus, G. Piper, T. Pirani, M. Pitchford, M. Pittman, S. Pitts, N. Plaatjies, N. Platt, R. Pleass, M. Plowright, L. Plummer, C. Plumptre, J. Pobjoy, T. Pogreban, C. Poku, S. Poku, P. Polgarova, R. Pollard, L. Pollock, O. Poluyi, G.J. Polwarth, F. Pomery, I.M.F. Ponce, P. Ponnusamy, S. Ponnusamy, A. Ponnuswamy, I. Ponte Bettencourt dos Reis, S. Pooboni, A. Poole, L. Poole, M. Poole, S. Poon, T. Poonian, J. Porteous, M. Porteous, D. Porter, J. Porter, L. Porter, R. Porter, A. Posada, K. Postlethwaite, M. Potdar, C. Pothecary, N. Pothina, P. Potla, D. Potoczna, J. Pott, A. Potter, J. Potter, S. Potter, T. Potter, E. Potton, J.B. Potts, J. Potts, K. Potts, K. Poudel, B. Poudyal, U. Poultney, K. Poulton, V. Poustie, J. Powell, N. Powell, S. Powell, D. Power, N. Power, S. Power, J. Poxon, E. Poyner, R. Poyner, A. Prabhu, S. Prabowo, V. Pradhan, G. Pradip, H. Prady, R. Prananingtias, A. Prasad, K. Prasad, U. Prasad, F. Prasanth Raj, S. Prasath, Prathima, N. Pratiwi, A. Pratley, S. Pratt, C.B. Prayuda, D. Preiss, C. Prendergast, L. Prentice, P. Prentice, V. Prescott, L. Presland, C. Prest, S. Preston, M. Pretorius, N. Prevatt, S. Prew, A. Price, C. Price, D. Price, E. Price, K. Price, L.J. Price, N. Price, V. Price, R. Price-Eland, A. Priest, J. Prieto, L. Primrose, C. Prince, J. Prince, L. Prince, S. Pringle, M. Prior-Ong, V. Pristopan, K. Pritchard, L. Pritchard, S. Pritchard, V. Priyash, A. Procter, C. Proctor, M. Protopapas, R. Proudfoot, B. Prudon, D. Pryor, S. Pudi, A. Puffett, J. Pugh, L. Pugh, M.T. Pugh, N. Pugh, R. Pugh, V. Puisa, E. Puji Lestari, S. Puliyakkadi, J. Pullen, K. Punia, S. Punnilath Abdulsamad, L. Purandare, D. Purchase, C. Purdue, R. Purdy, B. Purewal, R. Purnell, M. Pursell, G. Purssord, R. Purves, S. Purvis, K. Puspatriani, D. Putensen, S.I. Putu, B. Puvaneswaran, A. Puxty, K. Puxty, Z. Puyrigaud, E. Pyart, E. Pye, M. Pynn, T. Qadeer, M. Qayum, C. Quah, S. Quaid, N. Quail, C. Quamina, K. Quang, N.N. Quang, L. Quarm, G. Quartermaine, R. Quartey, T. Quasim, S. Quaye, A. Quayle, E. Quek, S. Quenby, P. Qui, X. Qui, V. Quick, J. Quigley, J.-C. Quijano-Campos, J. Quindoyos, A. Quinn, J. Quinn, T. Quinn, L.J. Quist, Q. Quratulain, D. Qureshi, E. Qureshi, H. Qureshi, I. Qureshi, K. Qureshi, N. Qureshi, Q. Qurratulain, S. Qutab, D.T.H. Quyen, D.T.N. Quyen, N.T.H. Quyen, M.S. Rabbani, S. Rabinowicz, M. Raceala, A. Rachid, B. Rachman, R. Rachman, L. Rad, J. Radford, L. Radford, J. Radhakrishnan, H. Rafferty, M.Y. Rafiq, S. Rafiq, C. Rafique, J. Rafique, M. Rafique, R. Ragatha, A. Raghunathan, A. Raguro, S.D. Raha, S. Rahama, M. Rahardjani, K. Rahilly, F. Rahim, A.H. Rahimi, H.R. Rahimi, M. Rahman, S.U. Rahman, S. Rahmany, P. Rai, S. Rai, L. Raisova, A. Raithatha, A. Raj, A. Rajagopal, P. Rajagopalan, N. Rajaiah, K. Rajalingam, A. Rajasekaran, A. Rajasri, B. Rajbhandari, S. Rajbhandari, T. Rajeswaran, J. Rajeswary, J. Rajkanna, I. Rajkumar, G. Rajmohan, R. Rallan, K. Ralston, M. Ralston, M. Ram, B. Ramabhadran, F. Ramali, M. Ramali, A. Ramanan, S. Ramanna, M. Ramasamy, I. Rambe, A. Ramchandani, D. Ramdin, J. Ramirez, M. Ramirez, G. Ramnarain, A. Ramnarine, L. Ramos, T. Rampling, S. Ramraj, J. Ramsay, A. Ramshaw, A. Rana, G.F. Rana, N. Rana, R. Rana, A. Rand, J. Rand, H. Randheva, P. Ranga, M. Rangar, H. Rangarajan, S. Ranjan, H. Rank, P. Ranka, R. Rankhelawon, A. Rankin, A. Rao, S. Rao, D. Rao, A.A. Rasheed, K. Rashid, M. Rason, V. Raspa, S. Rastogi, F. Rasul, S. Ratcliff, S. Ratcliffe, P. Rath, S. Rath, M.I. Rather, K. Rathod, S. Rathore, A. Ratnakumar, J. Ratoff, D. Rattehalli, D. Ravaccia, M. Raval, P. Ravencroft, J. Raw, R. Raw, M. Rawal, S.A. Rawashdeh, H. Rawlins, G. Ray, A. Raymond-White, D. Raynard, H. Rayner, N. Rayner, A. Raynsford, S. Razvi, Z. Razvi, K. Read, S. Read, M. Reay, A. Reddington, A. Reddy, H. Reddy, H. Redfearn, A. Redfern-Walsh, I. Redknap, N. Redman, A. Redome, J. Redome, A. Reed, J. Reed, A. Rees, C. Rees, H. Rees, J. Rees, M. Rees, S. Rees, T. Rees, E. Rees-Jones, F. Regan, K. Regan, M. Regan, S. Regan, K. Rege, A. Regmi, A. Rehan, A. Rehman, H. Rehman, S. Rehman, Z. Rehman, A. Reid, J. Reid, S. Reid, M. Reilly, S. Reilly, C. Reith, A. Reka, A. Remegoso, D. Rengan, L. Renouf, S. Renshaw, R. Renu Vattekkat, H. Reschreiter, M. Revels, A. Revill, G. Rewitzky, S. Rey, C. Reynard, D. Reynish, H. Reynolds, P. Reynolds, J. Rhodes, N. Riaz, P. Ribeiro, E. Rice, M. Rice, N. Rice, M. Rich, A. Richards, L. Richards, S. Richards, C. Richardson, E. Richardson, F. Richardson, J. Richardson, M. Richardson, N. Richardson, J. Riches, K. Riches, L. Richmond, R. Richmond, W. Ricketts, H. Rickman, A. Riddell, S. Ridgway, M. Ridha, C. Ridley, P. Ridley, G. Rieck, L. Rigby, M. Rigby, D. Rigler, S. Rijal, N. Rika, H. Riley, M. Riley, P. Riley, A. Rimainar, Z.V.P. Rimba, D. Rimmer, W. Rina, R. Rintoul, A. Riordan, D. Ripley, N. Rippon, C. Rishton, M. Riste, D. Ritchie, J. Ritchie, A. Ritchings, P. Rivera Ortega, V. Rivers, B. Rizvi, S.A.S. Rizvi, S.H.M. Rizvi, J. Robb, E. Robbins, C. Roberts, G. Roberts, I. Roberts, J. Roberts, K. Roberts, M. Roberts, N. Roberts, P. Roberts, R. Roberts, V. Roberts, C. Robertson, J. Robertson, J. Robertson, K. Robertson, N. Robertson, S. Robertson, M. Robertson, N. Robin, C. Robinson, E. Robinson, G. Robinson, H. Robinson, J. Robinson, K. Robinson, L. Robinson, M. Robinson, N. Robinson, R. Robinson, S. Robinson, A. Robinson, S. Robson, A. Rocca, L. Roche, S. Roche, N. Rodden, A. Roddick, E. Roddy, J. Roddy, M. Roderick, A. Rodger, F. Rodger, M. Rodger, M. Rodger, A. Rodgers, D. Rodgers, N. Rodgers, P. Rodgers, R. Rodriguez-Belmonte, N. Roe, C. Roehr, G. Rogers, J. Rogers, L. Rogers, M. Rogers, P. Rogers, S. Rogers, T. Rogers, J. Rojkova, K.K. Roka, S. Rokadiya, L. Rollins, J. Rollo, C. Rolls, A. Rond-Alliston, C. Rook, K. Rooney, L. Rooney, L.P. Rosaroso, E.J. Rosby, A. Rose, S. Rose, Z. Rose, J. Rosier, A. Roskilly, G.A. Ross, I. Ross, J. Ross, J. Rossdale, A. Ross-Parker, A. Rostron, A. Rosyid, A. Rothman, J. Rothwell, L. Roughley, C.A. Rourke, K. Rowan, N. Rowan, S. Rowan, A. Rowe, N. Rowe, L. Rowe-Leete, B. Rowlands, E. Rowlands, M. Rowley, S. Roy, M. Roycroft, A. Roynon-Reed, A.R. Royson, S. Rozewicz, A. Rudenko, S. Rudrakumar, B. Rudran, S. Ruff, P. Rughani, R. Rule, S. Rundell, E. Rushforth, J. Rushmer, D. Rusk, P. Russell, R. Russell, C. Russo, M. Rutgers, K. Rutkowski, A. Ryan, B. Ryan, K. Ryan, L. Ryan, M. Ryan, P. Ryan, D. Ryan-Wakeling, E. Rybka, M. Ryder, S. Ryder, M. Saad, G. Saalmink, J. Sabale, S. Sabaretnam, N. Sadiq, E. Sadler, A. Saffy, B. Sage, H. Sagoo, S. Sagrir, R. Saha, S. Saha, N. Sahdev, S. Sahedra, J. Sahota, N. Said, S. Saini, V. Saini, B. Saint, N. Sairam, A. Sajid, S. Sakthi, H. Sakuri, M. Saladi, A. Salam, A. Salberg, E. Salciute, G. Saleeb, M. Saleh, H. Salih, L. Salih, D. Salim, S. Salisbury, S. Saliu, R. Salman, J. Salmon, R. Salmon, D. Salutous, M. Sam, S. Sam, T. Samakomva, R. Saman, S. Samar, S. Saminathan, R. Samlal, E. Sammons, D. Sammut, M. Sammut, S. Sammut, T. Sammut, S. Sampath, C. Sampson, J. Sampson, A. Samson, J. Samuel, M. Samuel, R. Samuel, T.D.L. Samuel, Y. Samuel, K. Samuels, T. Samuels, J. Samways, M. Samyraju, I. Sana, V. Sanchez, A. Sanchez Gonzalez, A. Sanda-Gomez, P. Sandercock, J. Sanders, A. Sanderson, T. Sanderson, K. Sandhu, L. Sandhu, S. Sandow, V. Sandrey, S. Sands, L. Sanga, H. Sangha, J. Sanghera, M. Sangombe, M. Sanju, L. Sankaran, F. Santos, C. Santos Ferreira De Almeida, R. Santosh, J. Sanyal, A.F. Sanz-Cepero, Y. Sapkota, D. Saragih, D. Saralaya, A. Saraswati, A. Saraswatula, P. Saravanamuthu, S. Sarawade, J. Sarella, A. Sarfatti, R. Sargent, B. Sari, D. Sari, D. Sarkar, K. Sarkar, N. Sarkar, R. Sarkar, S. Sarma, P. Sarmiento, Z. Sarwar, T. Sass, K. Satchithananthasivam, S. Sathe, S. Sathianandan, A. Sathyanarayanan, S.J.P. Sathyanarayanan, T. Sathyapalan, P. Satodia, V. Saulite, A. Saunders, R. Saunders, S. Saunders, A. Saunderson, H. Savill, K. Savlani, G. Saxena, M. Saxton, A. Sayan, I. Sayers, D. Scaletta, D. Scanlon, J. Scanlon, L. Scarratt, S. Scattergood, A. Schadenberg, J. Schafers, W. Schneblen, E. Schofield, R. Schofield, S. Schofield, D. Scholes, K. Scholes, A. Schoolmeesters, N. Schumacher, N. Schunke, M. Schuster Bruce, K. Schwarz, A. Scobie, T. Scoones, T. Scorrer, A. Scott, A. Scott, C. Scott, E. Scott, K. Scott, L. Scott, M. Scott, S. Scott, T. Scott, Z. Scott, S. Scourfield, W. Scrase, N.A. Scriven, A. Scullion, T. Scullion, E. Seager, C. Seagrave, R. Seaman, E. Sear, I. Seaton, A. Seatter, A. Seckington, J. Sedano, G. Seddon, G. Sedgwick, Y. See, M.A. Seelarbokus, C. Sefton, M. Segovia, F. Seidu, G. Sekadde, F. Selby, G. Selby, C. Sellar, R. Sellars, K. Sellers, J. Selley, V. Sellick, G. Selvadurai, B. Selvarajah, H. Selvaskandan, S.S. Selvendran, J. Selwyn, A. Semmens, G. Semple, M. Sen, N. Sen, S. Sen, A. Sengupta, N. Sengupta, S. Senra, H. Senya, T. Serafimova, E. Sernicola, D. Sethi, S. Sethi, N. Setty, A. Seward, T. Sewdin, T.-A. Sewell, J. Seymour, K. Seymour, H. Shabbir, F. Shackley, T. Shafi, F. Shafique, A. Shah, A. Shah, B. Shah, H.-A. Shah, M. Shah, N. Shah, P. Shah, Q. Shah, R. Shah, S. Shah, S.H. Shah, W. Shah, S. Shahad, S. Shahi, S. Shahnazari, N. Shahzad, M. Shahzeb, A. Shaibu, Z. Shaida, A.Y. Shaikh, M. Shaikh, R. Shail, M. Shaji, M. Shakeel, R. Shakya, K. Shalan, M. Shameem, N. Shamim, U. Shamji, A. Shams, K. Shams, R. Shamsah, T. Shanahan, H. Sharaf, A. Sharif, A. Sharma, B. Sharma, M. Sharma, O. Sharma, P. Sharma, R. Sharma, S. Sharma, S.D. Sharma, S. Sharma, S. Sharma, A. Sharp, C. Sharp, G. Sharp, K. Sharp, L.M. Sharp, P. Sharratt, K. Sharrocks, S. Shashaa, A. Shaw, C. Shaw, D. Shaw, J. Shaw, L. Shaw, M. Shaw, T.G. Shaw, A. Shawcross, J. Shawcross, J. Shawe, L. Shayler, S. Shedwell, J. Sheffield, Z. Shehata, A. Sheik, A. Sheikh, N. Sheikh, B. Shelley, S. Shelton, A. Shenoy, J. Shenton, S. Shephardson, A. Shepherd, K. Shepherd, L. Shepherd, S. Shepherd, G. Sheppard, R. Sheppeard, H. Sheridan, R. Sheridan, S. Sherridan, L. Sherris, S. Sherwin, S. Shibly, F.F. Shiham, C. Shilladay, B. Shillitoe, D. Shingadia, C. Shioi, A. Shirgaonkar, K. Shirley, H. Shirt, A. Shonubi, J. Shoote, R. Shorrocks, R. Shortman, R. Shotton, S. Shotton, C. Shovelton, E. Shpuza, A. Shrestha, G. Shrestha, N. Shrestha, R. Shrestha, S. Shrestha, K. Shuker, J. Shurlock, J. Shurmer, E.R. Shuvo, S.K. Siabi, G. Siame, L. Siamia, M. Siaw-Frimpong, S. Siddavaram, N. Siddique, S. Siddique, S. Siddique, E. Siddle, E. Sidebotham, J. Sidebottom, R. Sievers, K. Siggens, N. Sikondari, I. Silanas, S.V. Silva, C. Silva Moniz, M. Sim, T. Simangan, V. Simbi, R. Sime, G. Simmons, O. Simmons, R. Simms, L. Simon, M. Simon, N. Simon, S. Simpkins, A. Simpson, A. Simpson, D. Simpson, G. Simpson, J. Simpson, K. Simpson, M. Simpson, P. Simpson, T. Simpson, K. Simpson, S. Sinclair, C. Sing, A. Singh, C. Singh, D. Singh, J. Singh, L. Singh, M. Singh, N. Singh, P. Singh, S. Singh, P. Singhal, B. Singizi, V. Singler, M. Sinha, P. Sinha, S. Sinha, U. Sinha, G. Sisson, S. Sithiravel, K. Sivakumar, S. Sivakumar, D. Sivakumran, S. Sivanadarajah, P.-R. Sivasothy, A. Skaria, N. Skehan, R. Skelly, O. Skelton, I. Skene, D. Skinner, T. Skinner, V. Skinner, A. Skorko, I. Skorupinska, M. Skorupinska, A. Slack, K. Slack, H. Slade, M. Slade, L. Slater, N. Slawson, R. Slingsby, A. Sloan, B. Sloan, D. Sloan, G. Sloane, M. Slowinska, B. Small, E. Small, S. Small, A. Smallridge, D. Smalls, K.D. Smallshaw, A. Smallwood, B. Smart, L. Smart, J. Smeaton, C. Smit, A. Smith, C. Smith, D. Smith, E. Smith, H. Smith, I. Smith, J. Smith, K. Smith, L. Smith, M. Smith, M.A. Smith, N. Smith, O. Smith, P. Smith, R. Smith, S. Smith, T. Smith, V. Smith, S. Smolen, S. Smuts, N. Smyth, A. Snell, D. Snell, L. Snell, A. So, B. So, M. Soan, R.F. Sobama, T. Sobande, S. Sobowiec Kouman, A. Sobrino Diaz, B. Sohail, H. Sohal, R. Soiza, O. Solademi, B. Soleimani, A. Solesbury, M. Soliman, B. Solis, R. Solly, L. Solomon, S. Somalanka, C. Somashekar, S. Sommerfield, G. Soni, R. Sonia, T. Sonoiki, S.-C. Soo, P. Soor, G. Soothill, J. Soren, A. Sothinathan, P. Sothirajah, J. Sousa, N. Soussi, D. Southam, D. Southern, I. Southern, L. Southern, S.M. Southin, J. Southwell, T. Southworth, S. Sowden, J. Sowter, C. Spalding, E. Spata, C. Speare, K. Spears, M. Spears, L. Speirs, S. Speirs, M. Spence, N. Spence, B. Spencer, G. Spencer, R. Spencer, S. Spencer, T. Spencer, H. Spickett, J. Spillane, W. Spiller, K. Spinks, M. Spinks, N. Spittle, S. Spray, J. Spriggs, O. Spring, G. Squires, J. Squires, R. Squires, R. Sreenivasan, S. Sreenivasan, M. Sri, K. Sri Paranthamen, R. Srikantaiah, K. Srinivasan, R. Srinivasan, A. Srirajamadhuveeti, V. Srirathan, S.K. Ssiabi, R. Stacey, S. Stacpoole, L. Stadon, W.J. Stagg, J. Staines, N. Staines, K. Stammers, R. Stanciu, G. Stanczuk, T. Standley, B. Staniforth, A. Stanton, L. Stanton, R. Staples, S. Stapley, N. Staplin, A. Stark, E. Starkey, D.S. Starnes, M. Starr, R. Stead, C. Stebbing, C. Steele, H. Steer, J. Steer, V. Stefania, P. Stefanowska, F. Steffensen, C. Stemp, E. Stenson, A. Stephens, D. Stephensen, E. Stephenson, M. Sterrenburg, J. Stevens, M. Stevens, W. Stevens, A. Stevenson, L. Stevenson, S. Stevenson, M. Steward, C. Stewart, D.A. Stewart, K. Stewart, M. Stewart, R. Stewart, J. Stickley, G. Stiller, S. Stirrup, S. Stock, A. Stockdale, D. Stocker, L. Stockham, P. Stockton, E. Stoddard, K. Stoffberg, C. Stokes, B. Stone, R. Stone, S. Stone, E.-J. Stoner, I. Storey, K. Storton, F. Stourton, A. Strachan, C. Strait, E. Stratton, J. Stratton, S. Straw, D. Streit, E. Stride, S. Stringer, S. Strong-Sheldrake, S. Struik, C. Stuart, A. Stubbs, H. Stubbs, A. Sturdy, S. Sturney, M. Stuttard, C. Suarez, K. Subba, C.P. Subbe, K. Subramaniam, M. Subramanian, V. Subramanian, C. Subudhi, R. Suckling, S. Sudershan, P. Sugden, P.A. Suherman, R. Sukla, A. Sukumaran, E. Suleiman, A. Suliman, F. Suliman, S. Sultan, U. Sumardi, S. Sundar, R. Sundaram, R. Sundhar, E. Sung, N. Sunni, J. Suntharalingam, A. Sur, D. Suresh, N. Suresh, S. Suresh, M. Surtees, C. Susan, D. Suter, R. Suthar, H. Sutherland, R. Sutherland, S. Sutherland, D. Sutinyte, D. Sutton, S. Sutton, M. Sutu, M.-L. Svensson, S. Svirpliene, A. Swain, R. Swain, T. Swaine, C. Swales, C. Swanson-Low, T. Swart, S. Sweetman, E. Swift, P. Swift, R. Swift, R. Swingler, S. Swinhoe, K. Swist-Szulik, L. Swithenbank, O. Syed, C. Sykes, D. Sykes, E. Sykes, L. Sylvester, D. Symington, D. Symon, A. Syndercombe, Z. Syrimi, J. Syson, G. Szabo, D. Szabó, T. Szakmany, N. Szarazova, M. Szekely, A. Szekeres, M. Szeto, K. Szymiczek, M. Tabish, M. Tadros, A. Tageldin, L. Tague, H. Tahir, M. Tahir, M. Tai, J. Tait, A. Takyi, P. Talbot, A. Talbot-Smith, J. Talbot-Ponsonby, R. Tallent, B. Tallon, A. Talukdar, A. Tan, B.T. Tan, H. Tan, J. Tan, J.S. Tan, K. Tan, W.T. Tan, A. Tana, A. Tanner, C. Tanney, T. Tanqueray, E. Tanton, A. Tantri, T. Tanzil-Al-Imran, H. Tarft, P. Taribagil, O. Tarin, S. Tariq, D. Tarpey, E. Tarr, L. Tarrant, A. Tasiou, A. Tate, M. Tate, M.L. Tate, P. Tate, K. Tatham, S.S. Tavares, V. Tavoukjian, S.A.I. Tay, A. Taylor, B. Taylor, C. Taylor, C.A. Taylor, D. Taylor, E. Taylor, H. Taylor, J. Taylor, K. Taylor, L. Taylor, M. Taylor, N. Taylor, R. Taylor, S. Taylor, T. Taylor, V. Taylor, M. Taylor-Siddons, T. Taynton, A. Te, F. Teasdale, J. Teasdale, K. Teasdale, J. Tebbutt, C. Tee, I. Teeluck, B. Tejero Moya, R. Tejwani, A. Telfer, V. Teli, J. Tempany, J. Temple, N. Temple, H. Tench, Y.H. Teoh, R. Tereszkowski-Kaminski, L. Terrett, L. Terry, T.I.M. Tesha, D. Tetla, S. Tewari, D. Tewkesbury, J. Texeira, C. Tey, P.N. Thach, M. Thake, C. Thakker, M. Thakker, J. Thakrar, B.J. Thakuri, B. Thamu, H. Thao, H.P. Thao, N.N. Thao, N. Thao, A. Thapa, H. Thatcher, A. Thayanandan, K. Thazhatheyil, E. Thein, L. Theocharidou, P. Thet, K. Thevarajah, M. Thevendra, V.T.K. Thi, D. Thien, N. Thiri Phoo, Y. Thirlwall, M. Thirumaran, A. Thomas, C. Thomas, E. Thomas, H. Thomas, J. Thomas, J.L. Thomas, K. Thomas, L. Thomas, R. Thomas, S. Thomas, T. Thomas, V. Thomas, K. Thomasson, R. Thomas-Turner, C. Thompson, E. Thompson, F. Thompson, H. Thompson, J. Thompson, K. Thompson, L. Thompson, M. Thompson, O. Thompson, R. Thompson, Y. Thompson, B.G. Thomson, N. Thomson, P. Thorburn, N. Thorn, C. Thorne, N. Thorne, A. Thornton, D. Thornton, J. Thornton, R. Thornton, S. Thornton, T. Thornton, C. Thorpe, N. Thorpe, S. Thorpe, P. Thozthumparambil, L. Thrasyvoulou, H. Thraves, N. Thu, N.M. Thu, G. Thueux, N. Thuong, P. Thu-Ta, D. Thuy, D.T.T. Thuy, V. Thwaiotes, C. Thwaites, C.L. Thwaites, G. Thwaites, S. Tiberi, S. Tieger, C. Tierney, M. Tighe, S. Tilbey, C. Till, A. Tiller, H. Tiller, J. Timerick, E. Timlick, A. Timmins, A. Timmis, H. Timms, A.-M. Timoroksa, S. Tinashe, S. Tingley, N. Tinker, H. Tinkler, M. Tinkler, J. Tipper, A. Tirumalai Adisesh, H. Tivenan, K. Tluchowska, H. T-Michael, A. Todd, J. Todd, S. Todd, O. Toffoletti, M. Tohfa, S. Tohill, M. Tolson, A. Tomas, N. Tomasova, S. Tomlin, S. Tomlins, J. Tomlinson, K. Tomlinson, J. Tonkin, I. Tonna, C. Toohey, K. Topham, M. Topping, A. Torokwa, C. Torrance, O. Touma, L. Tous Sampol, R. Tousis, M. Tout, P. Tovey, G. Towersey, J. Townley, R. Tozer, D.K. Tran, H. Tran, H.B. Tran, M. Tran, N. Tran, V.G. Tran, V.K. Tran, N.T.H. Trang, H. Tranter, J. Travers, C. Travill, S. Traynor, L. Trethowan, E. Treus Gude, M. Trevelyan, N.A. Trewick, A. Tridente, H. Trieu, S. Triggs, F. Trim, A. Trimmings, T. Trinick, S. Tripathy, K. Trivedi, S. Troedson, E. Tropman, A. Trotter, S. Trous, H. Trower, M. Trowsdale Stannard, N. Trudgill, R. Truell, N. Truman, M. Truslove, S. Trussell, T. Trussell, K. Tsakiridou, C. Tsang, P. Tsang, T. Tsawayo, K.K. Tsilimpari, G. Tsinaslanidis, M. Tsitsi, S. Tso, H.T.C. Tu, N. Tucker, S. Tucker, S. Tucker, D.E. Tudor, A. Tufail, J. Tuff, J. Tuffney, R. Tully, T. Tulus Satriasih, G. Tunesi, D. Tung, D.Q. Tung, K. Turbitt, R. Turel, T. Turgut, C. Turley, A. Turnbull, A. Turner, C. Turner, G. Turner, K. Turner, L. Turner, L.C. Turner, M. Turner, P. Turner, S. Turner, V. Turner, I. Turner-bone, S. Turney, J. Turvey, N.T.M. Tuyen, C. Tweed, D. Tweed, R. Twemlow, E. Twohey, B. Tyagi, V. Tyagi, A. Tyer, A. Tyler, J. Tyler, A. Tyzack, P. Tzavaras, I. Tzinieris, A.W. Uddin, M.S. Uddin, R. Uddin, J. Ugoji, E. Ukaegbu, M. Ul Haq, W. Ul Hassan, Z. Ul-Haq, S. Ullah, J. Um, A. Umaipalan, A. Umate, J. Umeadi, A. Umeh, W. Umeojiako, B. Ummat, E. Underhill, C. Underwood, J. Underwood, A. Unsworth, V. Uppal, V.S. Uppal, G. Upson, M. Ur Rasool, A. Uriel, S. Urruela, H. Uru, J. Usher, M. Usher, R. Usher, A. Usher-Rea, A. Ustianowski, E. Usuf, F. Utomo, H. Uzu, L.C. Vaccari, U. Vaghela, A. Vaidya, D. Vail, B. Valecka, J. Valentine, B. Valeria, P. Vallabhaneni, T. Valleri, N. Vallotton, L. Vamplew, E. Vamvakiti, J. Vamvakopoulos, C.T.C. Van, S. Van Blydenstein, L. van Bruggen, M. van de Venne, A. van der Meer, N. van der Stelt, R. Van Doorn, L. van Koutrik, A. Van Loggerenberg, J. Vance-Daniel, R. Vancheeswaran, S.I. Vandeyoon, P. Vankayalapati, P. Vanmali, C. Vansomeren, W. Van't Hoff, S. Vara, S.J. Vardy, A. Varghese, M. Varghese, W. Varney, G. Varnier, A.-N. Varouxaki, R. Varquez, V. Vasadi, O. Vass, K. Vassell, V. Vasu, V. Vasudevan, M. Vatish, S. Vaughan, H. Vayalaman, D. Vayapooree, C. Vaz, N. Veale, S. Veerasamy, S. Velankar, L. Velauthar, N. Veli, N. Vella, A. Velugupati, A. Velusamy, I. Venables, M. Venditti, R. Venkataramakrishnan, R. Venn, M. Venter, L. Ventilacion, J. Vere, M. Veres, S. Vergnano, W. Verling, A. Verma, R. Vernall, B. Vernon, M. Vertue, L. Verueco, J. Verula, A. Veterini, N. Vethanayagam, S. Vettikumaran, L. Veys, C. Vickers, S. Victor, S. Victoria, C. Vidaillic, C.P. Vidaillac, J. Vidler, B. Vijayakumar, V.W. Vijayaraghavan Nalini, B. Vilcinskaite, A. Vileito, N. Vilimiene, L. Vinall, S. Vinay, L. Vinayakarao, O. Vincent, R. Vincent, N.Q. Vinh, P. Virdee, E. Virgilio, A.M. Virk, E. Visentin, M. Vitaglione, K. Vithian, S. Vittoria, S. Vivekananthan, E. Vlad, B. Vlies, L. von Oven, C. Vooght, K.T. Vu Thai, K. Vutipongsatorn, A. Vuylsteke, E. Vyras, R. Wach, B. Wadams, S. Wadd, N. Waddington, P. Wade, J. Wadsley, K. Wadsworth, S.E.I. Wafa, D. Wagstaff, L. Wagstaff, D. Wahab, Z. Wahbi, A. Waheed Adigun, S. Waidyanatha, A. Waite, R. Wake, A. Wakefield, W. Wakeford, F. Wakinshaw, E. Waldeck, A. Walden, L. Walding, A. Waldron, J. Waldron, E. Wales, B. Wali, D. Walker, G. Walker, H. Walker, I. Walker, K. Walker, L. Walker, O. Walker, R. Walker, S. Walker, G. Wallace, R. Wallbutton, J. Wallen, K. Wallendszus, A. Waller, R. Waller, G. Wallis, L. Wallis, M. Wallis, E. Walmsley, D. Walsh, E. Walsh, L. Walsh, D. Walstow, D. Walter, A. Walters, H. Walters, J. Walters, E. Walton, L. Walton, M. Walton, O. Walton, S. Walton, M. Wan, J. Wanda, M. Wands, R. Wane, F. Wang, N. Wang, R. Wang, S. Wang, D. Warbrick, S. Warburton, C. Ward, D. Ward, E. Ward, H. Ward, J. Ward, L. Ward, N. Ward, R. Ward, T. Ward, S.A. Warden, G. Wardere, S. Wardle, H. Wardy, G. Waring, S. Waring, J. Warmington, B. Warner, C. Warner, L. Warnock, S. Warran, J. Warren, L. Warren, R. Warren, Y. Warren, D. Warrender, H. Warren-Miell, A. Warris, G. Warwick, H. Wassall, S. Wasserman, E. Wasson, H.J. Watchorn, H. Waterfall, A. Waters, D. Waters, M. Waterstone, A. Watkin, C. Watkins, E. Watkins, K. Watkins, L. Watkins, A. Watson, A.J.R. Watson, E. Watson, F. Watson, J.G.R. Watson, L. Watson, P. Watson, R. Watson, K. Watson, M. Watters, D. Watterson, K. Wattimena, D. Watts, J. Watts, M. Watts, V. Waugh, E. Wayman, M. Wayman, A. Wazir, M. Weatherhead, N. Weatherly, C. Webb, H. Webb, K. Webb, S. Webb, C. Websdale, D. Webster, I. Webster, J. Webster, T. Webster, J. Wedlin, L. Wee, R. Weerakoon, T. Weerasinghe, J. Weeratunga, M. Weetman, S. Wei, I. Weichert, E. Welch, H. Welch, J. Welch, L. Welch, S. Welch, B. Welham, S. Weller, L. Wellings, B. Wells, S. Wellstead, B. Welsh, R. Welsh, I. Welters, R. Welton, V. Wenn, L. Wentworth, J. Wesonga, K. Wesseldine, J. West, M. West, R. West, S. West, L. Western, R. Westhead, H. Weston, A. Westwood, K. Westwood, S. Westwood, B. Wetherill, S. Wheaver, H. Wheeler, B. Whelan, M. Whelband, A. Whileman, A. Whitcher, A. White, B. White, C. White, D. White, J. White, K. White, M. White, N. White, S. White, T. White, C. Whitehead, K. Whitehorn, A. Whitehouse, C. Whitehouse, T. Whitehouse, J. Whiteley, L. Whiteley, S. Whiteley, R. Whitham, G. Whitlingum, D. Whitmore, E. Whittaker, L. Whittam, A. Whittington, H. Whittle, R. Whittle, E. Wiafe, L. Wiblin, O. Wickens, J. Widdrington, J. Wieboldt, H. Wieringa, C. Wiesender, L. Wiffen, A. Wight, A. Wignall, C. Wignall, A. Wilce, D. Wilcock, E. Wilcock, L. Wilcox, B. Wild, L. Wild, S. Wild, M. Wilde, L. Wilding, P. Wilding, T. Wildsmith, J. Wileman, J. Wiles, K. Wiles, E. Wilhelmsen, T. Wiliams, J. Wilkie, D. Wilkin, H. Wilkins, J. Wilkins, S. Wilkins, I. Wilkinson, L. Wilkinson, N. Wilkinson, S. Wilkinson, T. Wilkinson, S. Willetts, A. Williams, C. Williams, C.V. Williams, D. Williams, E. Williams, G. Williams, H. Williams, J. Williams, K. Williams, M. Williams, P. Williams, R. Williams, S. Williams, T. Williams, S. Williams, A. Williamson, C. Williamson, D. Williamson, J. Williamson, J.D. Williamson, R. Williamson, C. Williamson, H. Williamson, E. Willis, H. Willis, J. Willis, L. Wills, L. Willsher, C. Willshire, F. Willson, J. Willson, A. Wilson, B. Wilson, D. Wilson, I. Wilson, J. Wilson, K. Wilson, K.-A. Wilson, L. Wilson, M. Wilson, S. Wilson, T. Wilson, J. Wilson, K.L.Y. Win, M. Win, T. Win, T.T. Win, W.Y.W. Win, L. Winckworth, L. Winder, P. Winder, S. Winearl, H. Winmill, S. Winn, C. Winpenny, H. Winslow, H. Winter, J. Winter, B. Winter-Goodwin, J. Winterton, H. Winwood, J. Wischhusen, S. Wisdom, M. Wise, M. Wiselka, R. Wiseman, S. Wiseman, S. Wishart, T. WIshlade, E. Witele, N. Withers, J. Wittes, D. Wixted, T. Wodehouse, W. Wolf, N. Wolff, K. Wolffsohn, R. Wolf-Roberts, E. Wolodimeroff, A. Wolstencroft, A. Wong, C. Wong, C.-H. Wong, C.-M. Wong, E. Wong, J.S.Y. Wong, K.Y. Wong, M.Y. Wong, N. Wong, S. Wong, T. Wong, A.A. Wongkyezeng, A. Wood, C. Wood, D. Wood, F. Wood, G. Wood, H. Wood, J. Wood, L. Wood, M. Wood, S. Wood, T. Wood, K. Woodall, R. Woodfield, C. Woodford, E. Woodford, J. Woodford, L. Woodhead, T. Woodhead, P. Woodland, M. Woodman, S. Woodmansey, C. Woods, J. Woods, K. Woods, S. Woods, Z. Woodward, M. Woolcock, G. Wooldridge, R. Woolf, C. Woollard, L. Woollen, E. Woolley, J. Woolley, D. Woosey, D. Wootton, J. Wootton, D. Worley, S. Worton, J. Wraight, M. Wray, K. Wren, L. Wren, C. Wrey Brown, C. Wright, D. Wright, F. Wright, H. Wright, I. Wright, L. Wright, R. Wright, S. Wright, T. Wright, C. Wroe, H. Wroe, H. Wu, P. Wu, J. Wubetu, F. Wulandari, R. Wulandari, S. Wurie, C. Wyatt, F. Wyn-Griffiths, I. Wynter, B. Xavier, A. Xhikola, B.E. Xia, Z. Xia, E. Yacoba, S. Yadav, M. Yakubi, M. Yan, Y. Yanagisawa, F. Yang, Y. Yang, M. Yanney, W.L. Yap, N. Yaqoob, S. Yasmin, B. Yates, D. Yates, E. Yates, H. Yates, T. Yates, M. Yates, J. Ye, C. Yearwood Martin, K. Yein, F. Yelnoorkar, L. Yen, L.M. Yen, A. Yeoh, C.Y. Yeung, P. Yew, D. Yewatkar, L. Ylquimiche Melly, I. Ynter, H. Yong, J. Yorke, J. Youens, A. Younes Ibrahim, E. Young, G. Young, L. Young, A. Yousafzar, S. Youssouf, A. Yousuf, H. Yovita, C. Yu, J.S.J. Yuan, N. Yufaniaputri, B. Yung, D. Yusef, S. Yusef, I. Yusuf, A.-S. Zafar, S. Zagalo, S. Zaher, A. Zahoor, M. Zainab, T. Zak, K. Zaki, N. Zakir, K. Zalewska, A. Zamalloa, M. Zaman, S. Zaman, J. Zamikula, L. Zammit, M. Zammit-Mangion, M. Zawadzka, M. Zayed, E. Zebracki, D. Zehnder, L. Zeidan, D. Zeinali, J. Zhang, X. Zhao, D. Zheng, D. Zhu, M. Zia, O. Zibdeh, R. Zill-E-Huma, E.T. Zin, E. Zincone, G. Zindoga, E. Zinkin, V. Zinyemba, C. Zipitis, L. Zitter, A. Zmierczak, G. Zubikarai, A. Zubir, N. Zuhra, R. Zulaikha, S. Zulfikar, C. Zullo, A. Zuriaga-Alvaro

**Keywords:** COVID-19, Corticosteroid, Dexamethasone, Mortality, Clinical trial

## Abstract

**Background:**

Low dose corticosteroids (e.g., 6 mg dexamethasone) have been shown to reduce mortality for hypoxic COVID-19 patients. We have previously reported that higher dose corticosteroids cause harm in patients with clinical hypoxia but not receiving ventilatory support (the combination of non-invasive mechanical ventilation, including high-flow nasal oxygen, continuous positive airway pressure and bilevel positive airway pressure ventilation, and invasive mechanical ventilation or extra-corporeal membrane oxygenation), but the balance of efficacy and safety in patients receiving ventilatory support is uncertain.

**Methods:**

This randomised, controlled, open-label platform trial (Randomised Evaluation of COVID-19 Therapy [RECOVERY]) assessed multiple possible treatments in patients hospitalised for COVID-19. Eligible and consenting adult patients receiving ventilatory support were randomly allocated (1:1) to either usual care with higher dose corticosteroids (dexamethasone 20 mg once daily for 5 days followed by 10 mg once daily for 5 days or until discharge if sooner) or usual standard of care alone (which includes dexamethasone 6 mg once daily for 10 days or until discharge if sooner). The primary outcome was 28-day mortality; secondary outcomes were duration of hospitalisation and (among participants not on invasive mechanical ventilation at baseline) the composite of invasive mechanical ventilation or death. Recruitment closed on 31 March 2024 when funding for the trial ended. The RECOVERY trial is registered with ISRCTN (50189673) and clinicaltrials.gov (NCT04381936).

**Findings:**

Between 25 May 2021 and 9 January 2024, 477 COVID-19 patients receiving ventilatory support were randomly allocated to receive usual care plus higher dose corticosteroids vs. usual care alone (of whom 99% received corticosteroids during the follow-up period). Of those randomised, 221 (46%) were in Asia, 245 (51%) in the UK and 11 (2%) in Africa. 143 (30%) had diabetes mellitus. Overall, 86 (35%) of 246 patients allocated to higher dose corticosteroids vs. 86 (37%) of 231 patients allocated to usual care died within 28 days (rate ratio [RR] 0.87; 95% CI 0.64–1.18; p = 0.37). There was no significant difference in the proportion of patients discharged from hospital alive within 28 days (128 [52%] in the higher dose corticosteroids group vs. 120 [52%] in the usual care group; RR 1.04, 0.81–1.33]; p = 0.78). Among those not on invasive mechanical ventilation at baseline, there was no clear reduction in the proportion meeting the composite endpoint of invasive mechanical ventilation or death (76 [37%] of 206 vs. 93 [45%] of 205; RR 0.79 [95% CI 0.63–1.00]; p = 0.05).

**Interpretation:**

In patients hospitalised for COVID-19 receiving ventilatory support, we found no evidence that higher dose corticosteroids reduced the risk of death compared to usual care, which included low dose corticosteroids.

**Funding:**

10.13039/100014013UK Research and Innovation (10.13039/501100000265Medical Research Council) and 10.13039/501100000272National Institute for Health Research (Grant ref: MC_PC_19056), and 10.13039/100010269Wellcome Trust (Grant Ref: 222406/Z/20/Z).

**Research in context**

**Evidence before this study**

We searched MEDLINE, Embase, MexRxiv and the WHO International Clinical Trials Registry Platform between Sept 1, 2019, and May 20, 2024 for randomised controlled trials comparing the effect of different doses of systemic corticosteroids in patients hospitalised with COVID-19 receiving ventilatory support at randomisation, using the search terms (Coronavirus infection OR SARS-CoV-2 OR SARS-CoV2 OR SARSCoV2 OR COVID OR COVID-19 OR COVID19 OR 2019-nCoV OR Coronavirus or Coronavirinae) AND (corticosteroid OR dexamethasone OR glucocorticoid OR steroid OR hydrocortisone OR methylprednisolone OR prednisolone OR betamethasone) and using validated filters to select for randomised controlled trials. We identified eleven relevant randomised trials with results available that assessed different doses of corticosteroids in hospitalised COVID-19 patients, at least some of whom were receiving ventilatory support at randomisation: six assessed higher dose dexamethasone (12–24 mg per day) and five assessed methylprednisolone (60–1000 mg per day), all compared to lower dose dexamethasone (6–8 mg per day). Ten of the trials have been fully published of which six were considered to have low risk of bias for the 28-day mortality outcome and four having some concerns (including uncertainty about allocation concealment, lack of information about pre-specified analyses, crossover between randomised groups and post-randomisation exclusion of patients in the analysis population) (Supplementary Appendix pp 33–34). All but one of the trials found no statistically significant difference in mortality between the treatment groups.

**Added value of this study**

The Randomised Evaluation of COVID-19 Therapy (RECOVERY) trial is the largest randomised trial of the effect of different doses of corticosteroids in patients hospitalised with COVID-19 and included patients from 3 continents. We found that among patients receiving ventilatory support, randomisation to higher dose corticosteroids (dexamethasone 20 mg daily for 5 days followed by dexamethasone 10 mg for 5 days, or until discharge if sooner) vs. usual care (which included dexamethasone 6 mg once daily in 87% of participants) did not significantly reduce the risk of all-cause mortality. Overall, 86 (35%) of 246 patients in the higher dose corticosteroid group died vs. 86 (37%) of 231 patients in the usual care group (rate ratio 0.87; 95% CI 0.64–1.33; p = 0.37).

**Implications of all the available evidence**

Among hospitalised patients with COVID-19 who require oxygen or ventilatory support, low dose corticosteroids reduce the risk of death. The currently available randomised data provide no evidence that among patients receiving ventilatory support, higher dose corticosteroids further improve clinical outcomes, although the number of patients included in such trials is relatively small so this conclusion could change should further large-scale randomised data become available. There is therefore currently no clear indication to use higher dose corticosteroids in patients with COVID-19.

## Introduction

The RECOVERY trial has previously shown that the use of corticosteroids (using dexamethasone 6 mg once daily for ten days or until discharge if sooner) reduces the risk of death in patients admitted to hospital with COVID-19 and clinical hypoxia.^1^ Subsequent findings that additional immunosuppression with an interleukin-6 (IL-6) receptor blocker and/or a Janus kinase (JAK) inhibitor further reduces the risk of death in these patients raised the question whether simply increasing the dose of corticosteroid rather than adding other immunomodulators could confer additional benefits at substantially lower cost.^2^^,^^3^

In April 2021, the United Kingdom COVID-19 Therapeutics Advisory Panel recommended that the RECOVERY trial study higher dose corticosteroids.^4^ The RECOVERY trial therefore established a randomised evaluation of the effects of higher dose corticosteroids vs. usual care for adult patients who had been admitted to hospital with COVID-19 and had clinical evidence of hypoxia. Usual care for hypoxic COVID-19 patients includes low dose corticosteroids. On 11 May 2022, the independent Data Monitoring Committee recommended that this comparison be halted for those patients receiving no oxygen or simple oxygen only on the grounds of safety and the results among such patients were published, showing that higher dose corticosteroids were associated with an increased 28-day mortality (123/659 [19%] in those allocated higher dose corticosteroids vs. 75/613 [12%] in those allocated usual care, rate ratio 1.59; 95% confidence interval [CI] 1.20–2.10; p = 0.0012.^5^ Recruitment of patients receiving ventilatory support continued.

Trials of corticosteroids in non-COVID acute respiratory distress syndrome (ARDS) have not directly compared higher doses with the lower dose used in RECOVERY.^6^ Higher doses of corticosteroids have been assessed in other trials among patients with COVID-19 receiving ventilatory support, but none have demonstrated clear benefit or harm.^7^, ^8^, ^9^, ^10^, ^11^ A World Health Organization meta-analysis of randomised controlled trials in critically ill COVID-19 indicated similar mortality benefit with lower and higher dose corticosteroids, but estimates were imprecise.^12^

Here we report the results of the randomised comparison from the RECOVERY trial comparing higher dose corticosteroids with usual care among patients receiving ventilatory support.

## Methods

### Study design and participants

The Randomised Evaluation of COVID-19 therapy (RECOVERY) trial is an investigator-initiated, individually randomised, controlled, open-label, platform trial to evaluate the effects of potential treatments in patients hospitalised with COVID-19. Details of the trial design and results for other possible treatments (dexamethasone, hydroxychloroquine, lopinavir-ritonavir, azithromycin, tocilizumab, convalescent plasma, colchicine, aspirin, casirivimab plus imdevimab, baricitinib, higher dose corticosteroids among patients not receiving ventilatory support, empagliflozin, molnupiravir and nirmatrelvir-ritonavir) have been published previously.^1^, ^2^, ^3^^,^^5^^,^^13^, ^14^, ^15^, ^16^, ^17^, ^18^, ^19^, ^20^, ^21^ The trial was conducted at hospital organisations in the United Kingdom, supported by the National Institute for Health Research Clinical Research Network, as well as in South and Southeast Asia and Africa. Of these, 81 hospitals in the UK, 5 in Nepal, 2 in Indonesia, 2 in Vietnam, 2 in South Africa, and 1 in Ghana enrolled participants in the evaluation of higher dose corticosteroids (Supplementary Appendix pp 5–31). The trial was coordinated by the Nuffield Department of Population Health at the University of Oxford (Oxford, UK), the trial sponsor. The trial was conducted in accordance with the principles of the International Conference on Harmonisation Good Clinical Practice guidelines. The protocol was approved by all relevant regulatory authorities and ethics committees in each participating country (Supplementary Appendix p 32) The protocol and statistical analysis plan are available in the Supplementary Appendix (pp 69–151) with additional information available on the study website www.recoverytrial.net.

Patients aged at least 18 years admitted to hospital were eligible for the study if they had clinically suspected or laboratory confirmed SARS-CoV-2 infection, clinical evidence of hypoxia (i.e., receiving oxygen with or without other forms of respiratory support, or with oxygen saturations <92% on room air) and no medical history that might, in the opinion of the attending clinician, put the patient at significant risk if they were to participate in the trial. Patients were ineligible for the comparison of higher dose corticosteroid vs. usual care if there was a known contra-indication to short-term use of corticosteroids, suspected or confirmed influenza, or current use of nirmatrelvir-ritonavir, ritonavir or other potent CYP3A inhibitors. Ventilatory support includes continuous positive airway pressure, bi-level positive airway pressure, high-flow nasal oxygen (HFNO, together non-invasive ventilation), invasive mechanical ventilation and extracorporeal membrane oxygenation (ECMO). Endemic infections were screened for in accordance with local practice. Other immunomodulatory therapies were not contra-indicated but investigators were advised to consider the total burden of such therapy (e.g., combining an IL-6 receptor antagonist with higher dose corticosteroid). Written informed consent was obtained from all patients, or a legal representative if patients were too unwell or unable to provide consent.

### Randomisation and masking

Baseline data were collected using a web-based case report form that included demographics, level of respiratory support, major comorbidities, suitability of the study treatment for a particular patient, SARS-CoV-2 vaccination status, and treatment availability at the study site (Supplementary Appendix pp 46).

Eligible and consenting patients were assigned in a 1:1 ratio to either usual standard of care plus higher dose corticosteroids or usual standard of care alone (which included low dose corticosteroids, usually dexamethasone 6 mg once daily for 10 days or until discharge if sooner), using web-based simple (unstratified) randomisation with allocation concealed until after randomisation (Supplementary Appendix pp 41–5).^22^^,^^23^ Patients allocated to higher dose corticosteroid were to receive dexamethasone 20 mg daily for 5 days followed by dexamethasone 10 mg for 5 days (or until discharge if sooner). Alternative corticosteroid regimens for pregnant women are described in the protocol (Supplementary Appendix p 69–111).

As a platform trial, and in a factorial design, patients could be simultaneously randomised to other treatment groups: i) empagliflozin vs. usual care, ii) sotrovimab vs. usual care, and iii) molnupiravir vs. usual care. Further details of when these factorial randomisations were open are provided in the Supplementary Appendix (pp 41–43). Participants and local study staff were not masked to the allocated treatment. Other than members of the Data Monitoring Committee, all individuals involved in the trial were masked to aggregated outcome data while recruitment and 28-day follow-up were ongoing.

### Procedures

An online follow-up form was completed by site staff when patients were discharged, had died, or at 28 days after randomisation, whichever occurred first (Supplementary Appendix pp 47–55). Information was recorded on adherence to allocated trial treatment, receipt of other COVID-19 treatments, duration of admission, receipt of respiratory or renal support, new cardiac arrhythmia, thrombosis, clinically significant bleeding, non-COVID infection, metabolic complications (collected from 28 July 2021 onwards), and vital status (including cause of death). In addition, in the UK, routinely collected healthcare and registry data were obtained, including information on vital status at day 28 (with date and cause of death); discharge from hospital; and receipt of respiratory support or renal replacement therapy. For sites outside the UK a further case report form (Supplementary Appendix p 56) collected vital status at day 28 (if not already reported on follow-up form).

### Outcomes

Outcomes were assessed at 28 days after randomisation, with further analyses specified at 6 months. The primary outcome was 28-day all-cause mortality. Secondary outcomes were time to discharge from hospital, and, among patients not on invasive mechanical ventilation at randomisation (which for this report means those who were on non-invasive mechanical ventilation at randomisation), the composite outcome of invasive mechanical ventilation (including extra-corporeal membrane oxygenation) or death. Prespecified subsidiary clinical outcomes were successful cessation of invasive ventilation among those receiving invasive ventilation at randomisation (defined as extubation and survival to day 28 without re-intubation), use of invasive mechanical ventilation among patients on non-invasive ventilation at randomisation, and use of renal dialysis or haemofiltration. Prespecified safety outcomes were cause-specific mortality, major cardiac arrhythmia, thrombotic and major bleeding events, other infections and metabolic complications. Information on suspected serious adverse reactions was collected in an expedited fashion to comply with regulatory requirements. Details of the methods used to ascertain and derive outcomes are provided in the Supplementary Appendix (pp 152).

### Sample size and role of the independent Data Monitoring Committee

As stated in the protocol, appropriate sample sizes could not be estimated when the trial was being planned. However, the intention for this comparison was to continue recruitment until sufficient primary outcomes had accrued to have 90% power to detect a proportional risk reduction of 20% at 2p=0.01. The trial was stopped early due to funding constraints so did not reach this sample size.

The independent Data Monitoring Committee reviewed unblinded analyses of the study data and any other information considered relevant to the trial at intervals of around 2–3 months (depending on speed of enrolment) and was charged with determining if, in their view, the randomised comparisons in the study provided evidence on mortality that was strong enough (with a range of uncertainty around the results that was narrow enough) to affect national and global treatment strategies (Supplementary Appendix p 58).

On 11 May 2022, the Data Monitoring Committee recommended stopping recruitment to the higher dose corticosteroid comparison for patients who required no oxygen or simple oxygen only at randomisation due to safety concerns (Supplementary Appendix p 59). The Data Monitoring Committee encouraged continuing recruitment of all those patients who, at randomisation, required either non-invasive ventilation or invasive mechanical ventilation. Recruitment continued until 31 March 2024 when funding for the trial ended.

### Statistical analysis

All analyses in this report were limited to the subgroup of patients receiving ventilatory support at randomisation. Intention-to-treat analyses compared patients randomised to higher dose corticosteroids with patients randomised to usual care. For the primary outcome of 28-day mortality, the hazard ratio (HR) from a Cox model with adjustment for age in three categories (<70 years, 70–79 years, and 80 years or older) and ventilation status at randomisation in two categories (non-invasive ventilation and invasive mechanical ventilation) was used to estimate the mortality rate ratio (There was no evidence against the proportional hazards assumption.) We constructed Kaplan–Meier survival curves to display cumulative mortality over the 28-day period (starting on the day of randomisation and ending 28 days later). We used the same Cox regression method to analyse time to hospital discharge and successful cessation of invasive mechanical ventilation, with patients who died in hospital right-censored on day 29. Median time to discharge was derived from Kaplan–Meier estimates. For the composite secondary outcome of progression to invasive mechanical ventilation or death within 28 days, and the subsidiary clinical outcomes of receipt of ventilation and use of haemodialysis or haemofiltration, the precise dates were not available and a log-binomial regression model was used to estimate the risk ratio (RR) adjusted for age and ventilation status. Estimates of rate and risk ratios (both denoted RR) are shown with 95% confidence intervals. For safety outcomes, unadjusted absolute risk differences were calculated as the difference in the proportions of patients experiencing outcomes by treatment allocation.

Since the analyses presented here relate only to the subgroup of participants who required ventilatory support at randomisation, any analyses of the primary outcome in further subgroups defined by different baseline characteristics must be considered exploratory in nature. With that caveat, we present analyses of the primary outcome by age, sex, ethnicity, country, days since symptom onset, and respiratory support received with tests of heterogeneity or trend, as appropriate. We have not presented analyses by subgroups for the secondary or other outcomes. Results for the pre-specified other clinical outcomes and safety outcomes are presented. For the primary outcome of 28-day mortality, the results from RECOVERY were subsequently included in a meta-analysis of results from all previous randomised trials of higher vs. lower dose steroids in patients with COVID-19. For each trial, we compared the observed number of deaths among patients allocated higher dose steroids with the expected number if all patients were at equal risk (i.e., we calculated the observed minus expected statistic [o–e], and its variance v). For the previously-reported RECOVERY findings in lower risk (not on ventilation) patients, these were taken as the log-rank observed minus expected statistic and its variance but for other trials, where the exact timing of each death was not available, these were calculated from standard formulae for 2 × 2 contingency tables. We then combined trial results using the log of the mortality rate ratio calculated as the inverse-variance weighted average S/V with variance 1/V (and hence with 95% CI S/V ± 1.96/√V), where S is the sum over all trials of (O–E) and V is the sum over all trials of v. Finally, for the primary outcome of 28-day mortality we report a *post hoc* analysis limited to patients who received treatment exactly as allocated. Such “per protocol” analyses can be seriously biased,^24^ but it has been included at the request of the journal editor and reviewers.

The full database is held by the study team which collected the data from study sites and performed the analyses at the Nuffield Department of Population Health, University of Oxford (Oxford, UK). Analyses were performed using SAS version 9.4 and R version 4.0.3. The trial is registered with ISRCTN (50189673) and clinicaltrials.gov (NCT04381936).

### Role of the funding source

The funders of the study had no role in study design, data collection, data analysis, data interpretation, or writing of the report. The corresponding authors had full access to all the data in the study and had final responsibility for the decision to submit for publication.

## Results

**Fig. 1:**
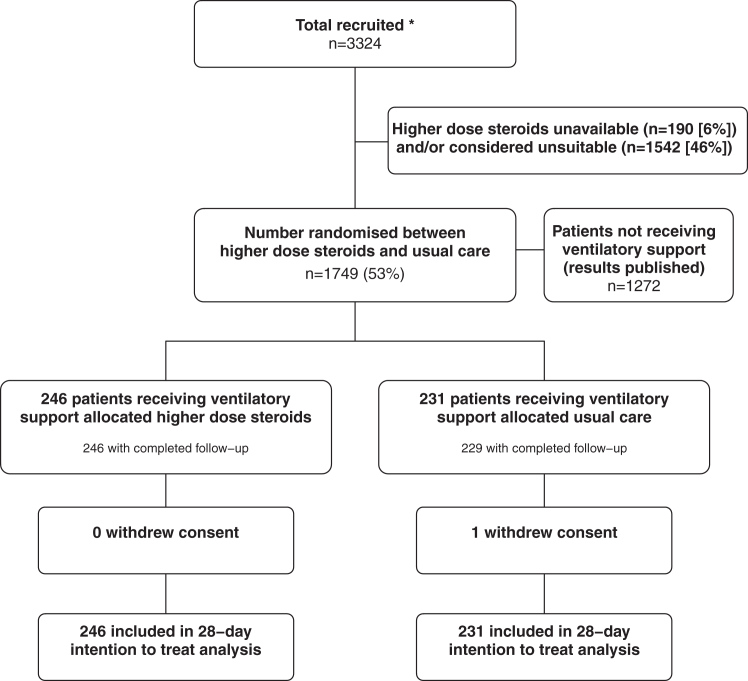
Trial profile. ITT = intention to treat. Higher dose corticosteroid unavailable and higher dose corticosteroid considered unsuitable are not mutually exclusive. Ventilatory support defined as non-invasive ventilation or invasive mechanical ventilation at randomisation. ∗ Number recruited overall during period that adult participants could be recruited into the higher dose corticosteroid comparison.

Recruitment to the evaluation of higher dose corticosteroids commenced on 25 May 2021 outside the UK and 29 December 2021 in the UK (following closure of the baricitinib comparison) and ended worldwide on 31 March 2024 (last participant randomised on 9 January 2024). Of 1749 patients enrolled in this comparison during this period, 477 patients receiving ventilatory support are included in this evaluation. Of these, 246 were randomly allocated to higher dose corticosteroids and 231 patients were randomly allocated to usual care (Fig. 1). The mean age of these participants was 61.4 years (SD 15.5), 221 (46%) were recruited in Asia, 245 (51%) in the UK and 11 (2%) in Africa. 411 (86%) were receiving non-invasive ventilation and 66 (14%) were receiving invasive mechanical ventilation. 143 (30%) had a history of diabetes mellitus (Table 1).

**Table 1:** Baseline characteristics by treatment allocation.

	Higher dose steroids (n = 246)	Usual care (n = 231)
Age, years	61.7 (14.9)	61.1 (16.1)
<70	164 (67%)	157 (68%)
≥70 to <80	61 (25%)	46 (20%)
≥80	21 (9%)	28 (12%)
Sex		
Male	147 (60%)	151 (65%)
Female	99 (40%)	80 (35%)
Country		
Indonesia	28 (11%)	37 (16%)
Nepal	44 (18%)	42 (18%)
South Africa	8 (3%)	3 (1%)
Vietnam	40 (16%)	30 (13%)
United Kingdom	126 (51%)	119 (52%)
Ethnicity		
White	107 (43%)	96 (42%)
Black	1 (<0.5%)	5 (2%)
Asian	77 (31%)	87 (38%)
Other	4 (2%)	3 (1%)
Unknown	57 (23%)	40 (17%)
Number of days since symptom onset	8 (5–12)	7 (5–12)
Number of days since admission to hospital	2 (1–4)	2 (1–4)
Respiratory support received		
Non-invasive ventilation	206 (84%)	205 (89%)
Continuous positive airway pressure	61 (25%)	56 (24%)
Bilevel positive airway pressure	36 (15%)	35 (15%)
High-flow nasal oxygen	109 (44%)	114 (49%)
Invasive mechanical ventilation	40 (16%)	26 (11%)
Previous diseases		
Diabetes	74 (30%)	69 (30%)
Heart disease	77 (31%)	69 (30%)
Chronic lung disease	57 (23%)	46 (20%)
Tuberculosis	1 (<0.5%)	2 (1%)
HIV	4 (2%)	1 (<0.5%)
Severe liver disease^a^	7 (3%)	4 (2%)
Severe kidney impairment^b^	22 (9%)	14 (6%)
Any of the above	159 (65%)	134 (58%)
SARS-CoV-2 test result		
Positive	232 (94%)	213 (92%)
Negative	2 (1%)	3 (1%)
Unknown	12 (5%)	15 (6%)
Received a COVID-19 vaccine	133 (54%)	112 (48%)
Use of other treatments^c^		
Remdesivir	88 (36%)	78 (34%)
Tocilizumab	57 (23%)	51 (22%)
Plan to use tocilizumab within the next 24 h	23 (9%)	17 (7%)

Data are mean (SD), n (%), or median (IQR). 0 pregnant women were randomised.

a Defined as requiring ongoing specialist care.

b Defined as estimated glomerular filtration rate <30 mL/min per 1.73 m^2^.

c Information on JAK (Janus kinase) inhibitor use was only collected for 99 participants, of whom 11 were receiving a JAK inhibitor and 88 were not.

The follow-up form was completed for 246 (100%) patients in the higher dose corticosteroid group and 229 (99.1%) patients in the usual care group. Among patients with a completed follow-up form, 87% allocated to higher dose corticosteroid were reported to have received higher dose corticosteroids compared with 6% allocated to usual care (Fig. 1, webtable 2). Among those with a completed follow-up form allocated usual care, 68% received low dose and 18% received intermediate dose (>6 < 20 mg) dexamethasone. Overall, 39% received remdesivir, 15% received an interleukin-6 antagonist and 5% received baricitinib during the follow-up period (webtable 2).

**Fig. 2:**
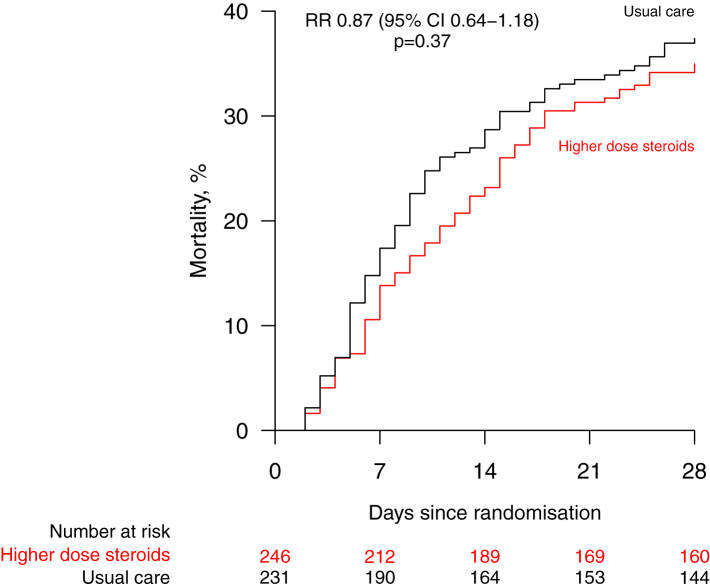
Effect of allocation to higher dose corticosteroids or usual care (lower dose corticosteroids) on 28-day mortality in patients receiving ventilatory support. RR = rate ratio. Estimates are taken as 100 minus the Kaplan–Meier estimated survival percentage.

**Fig. 3:**
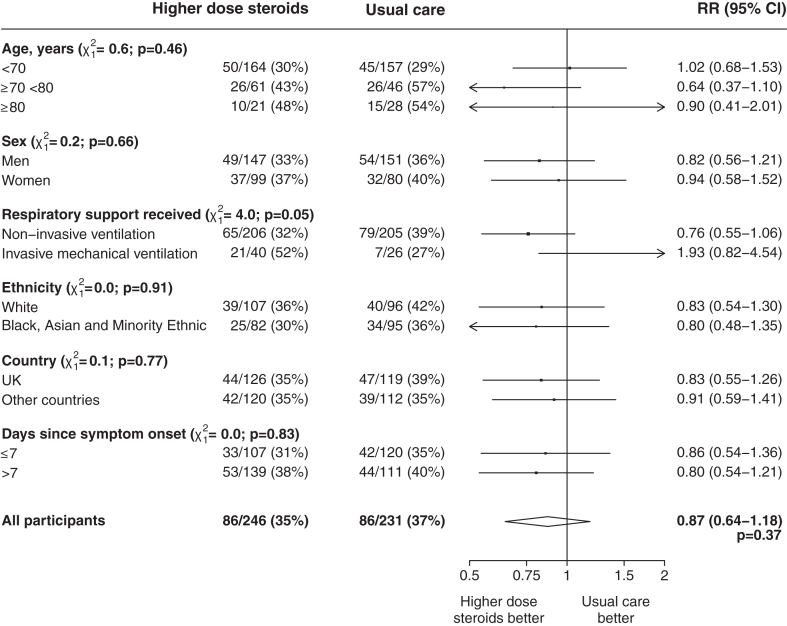
Effect of allocation to higher dose corticosteroids or usual care (lower dose corticosteroids) on 28-day mortality in patients receiving ventilatory support by other baseline characteristics. Subgroup−specific rate ratio estimates are represented by squares (with areas of the squares proportional to the amount of statistical information) and the lines through them correspond to the 95% CIs. RR = rate ratio.

Primary and secondary outcome data are known for >99% of randomly assigned patients. There was no clear evidence that allocation to higher dose corticosteroids reduced the risk of the primary outcome of 28-day mortality compared with usual care alone: 86 (35%) of 246 patients in the higher dose corticosteroid group died vs. 86 (37%) of 231 patients in the usual care group (rate ratio 0.87; 95% CI 0.64–1.18; p = 0.37; Table 2, Fig. 2). An exploratory sensitivity analysis which excluded participants receiving HFNO did not materially change this finding (RR 0.97; 95% CI 0.66–1.41). In other exploratory analyses, there was no good evidence that the proportional effect of higher dose corticosteroids on mortality differed across all 5 pre-specified subgroups, nor by country (Fig. 3). Stratifying the analysis by country in a post hoc sensitivity analysis did not materially affect the results (RR 0.84; 95% CI 0.62–1.14). In another exploratory analysis, there was also no good evidence of heterogeneity according to baseline use (or planned use) of tocilizumab. Among the 148 participants who received tocilizumab (or there was a plan to do so within 24 h) the rate ratio was 0.66 (95% CI 0.38–1.13); for the other 329 participants the rate ratio was 0.99 (95% CI 0.59–1.43; p-value for heterogeneity = 0.22). The *post hoc* ‘per protocol’ analysis limited to the 370 patients who received treatment exactly as allocated resulted in an adjusted RR of 0.90 (95% CI 0.63–1.28).

**Table 2:** Effect of allocation to higher dose corticosteroid on key study outcomes.

	Treatment allocation		RR (95% CI)
	Higher dose steroids (n = 246)	Usual care (n = 231)	
**Primary outcome**			
28-day mortality	86 (35%)	86 (37%)	0.87 (0.64–1.18)
**Secondary outcomes**			
Time to being discharged alive, days (median [IQR])	24 (12 to >28)	26 (11 to >28)	
Discharged from hospital within 28 days	128 (52%)	120 (52%)	1.04 (0.81–1.33)
Receipt of invasive mechanical ventilation or death^a^	76/206 (37%)	93/205 (45%)	0.79 (0.63–1.00)
Invasive mechanical ventilation	29/206 (14%)	40/205 (20%)	0.72 (0.46–1.11)
Death	65/206 (32%)	79/205 (39%)	0.80 (0.62–1.04)
**Subsidiary clinical outcomes**			
Successful cessation of invasive mechanical ventilation^b^	14/40 (35%)	11/26 (42%)	0.80 (0.36–1.80)
Use of haemodialysis or haemofiltration^c^	21/243 (9%)	18/229 (8%)	0.97 (0.54–1.74)

Data are n (%) or n/N (%), unless otherwise indicated. RR = rate ratio for the outcomes of 28-day mortality and hospital discharge, and risk ratio for other outcomes. CI = confidence interval. Estimates of the RR and its 95% CI are adjusted for age in three categories (<70 years, 70–79 years, and 80 years or older) and ventilation status at randomisation in two categories (non-invasive ventilation and invasive mechanical ventilation).

a Analyses exclude those on invasive mechanical ventilation at randomisation.

b Analyses restricted to those on invasive mechanical ventilation at randomisation.

c Analyses exclude those on haemodialysis or haemofiltration at randomisation.

Discharge alive within 28 days was similar among those allocated to higher dose corticosteroids compared with usual care (52% vs. 52%; rate ratio 1.04, 95% CI 0.81 to 1.33; median 24 days vs. 26 days) (Table 2). Among participants not on invasive mechanical ventilation (i.e., on non-invasive ventilation) at randomisation, there was no clear evidence that allocation to higher dose corticosteroids reduced the risk of progressing to the composite secondary outcome of invasive mechanical ventilation or death (37% vs. 45%, risk ratio 0.79, 95% CI 0.63 to 1.00) (Table 2). There were no significant differences in successful cessation of invasive mechanical ventilation among patients on invasive mechanical ventilation at randomisation, or receipt of haemodialysis or haemofiltration (Table 2).

137 (80%) of the deaths within 28 days were attributed to COVID-19 (webtable 3). There was no clear evidence that allocation to higher dose corticosteroids increased the risk of reported secondary infections (29% vs. 21%, absolute risk increase 7.6%, 95% CI -0.1 to 15.4%; webtable 4) including pneumonia reported as not due to COVID-19 (21% vs. 14%, absolute risk increase 6.4%, 95% CI -0.3 to 13.2%). There were no significant differences in the rates of new onset cardiac arrhythmia, thrombotic events, clinically significant bleeding or metabolic complications (including hyperglycaemia receiving new use of insulin) (webtable 4). There were 32 reports (among 19 participants) of a serious adverse reaction believed to be related to treatment with higher dose corticosteroids (webtable 5), including 15 reports of a serious non-COVID infection, 5 with hyperglycaemia and 4 with gastro-intestinal bleeding.

Our meta-analysis identified 142 deaths among 657 participants in 5 other trials assessing higher dose corticosteroids among patients requiring ventilation (webfigure 1). Our results appeared to be consistent with the other limited data and overall there was no evidence that higher dose corticosteroids were beneficial in these patients (overall ratio of death rates 0.87, 95% CI 0.69–1.11).

## Discussion

In this randomised trial among patients with COVID-19 receiving ventilatory support, we found no evidence that allocation to higher dose corticosteroids reduced 28-day mortality, duration of hospitalisation or, among patients not on invasive mechanical ventilation at baseline, the composite outcome of use of invasive mechanical ventilation or death.

The non-significant effects observed in this population of patients contrast with the significant hazards observed among patients not receiving ventilatory support reported previously.^5^ There is statistical heterogeneity (p=0.007) between the estimates of the effect of allocation to higher dose corticosteroids on 28 day mortality in these two populations: 0.87 (95% CI 0.64–1.18) among patients receiving ventilatory support vs. 1.59 (95% CI 1.20–2.10) among those not. It is possible that the reported result among patients not receiving ventilatory support is an over-estimate of the true effect because an interim analysis triggered the decision to stop enrolment in that group, and therefore the true heterogeneity between these two populations may not be so marked.^25^ Although there is clear evidence of effect modification by baseline disease severity with low dose corticosteroids,^1^ the subgroup analysis within the comparison reported here does not suggest that there is increasing benefit with increasing severity of disease with if anything the reverse being seen, albeit based on a very small number of outcomes.

Results from RECOVERY and other trials have shown that combining immunomodulatory therapies can provide additional benefit with both tocilizumab and baricitinib providing additional benefits over low dose corticosteroids.^2^^,^^3^^,^^26^ These drugs are not widely available nor affordable in low- and middle-income countries whereas higher doses of corticosteroids are. However, we found no evidence that higher doses of corticosteroids were beneficial, even in the absence of tocilizumab, so our results do not support their use as a more affordable option where such therapies are not available.

Although the difference in secondary infections was not significant in this comparison (absolute risk increase 7.7%, 95% CI -0.1 to 15.4), the proportional increase of about one-third was similar to that observed among patients not requiring ventilatory support. Therefore when considered in combination it is clear that higher dose corticosteroids are associated with more infections than usual care that includes low dose corticosteroids. Similarly, although the excess of hyperglycaemia requiring new use of insulin reported among patients not receiving ventilatory support was not observed in the current population, this may have been due to the small number of events or due to use of insulin infusions in some intensive care settings.

The clinical presentation of patients hospitalised with COVID-19 has changed during the course of the pandemic due to evolution of SARS-CoV-2, vaccination and herd immunity and the development of treatments. Consequently, patients with florid pneumonitis and respiratory failure are less frequent than in the early years meaning that the need for ventilatory support has reduced substantially.^27^

Strengths of the RECOVERY trial are that it is randomised, has a relatively large sample size (albeit not as large as originally intended due to funding constraints), broad eligibility criteria and more than 99% of patients in this analysis have been followed up for the primary outcome. The trial was also conducted in areas with high (south and southeast Asia, and Africa) and low (UK) prevalence of tuberculosis and other infections. The study has some limitations: it was not possible to recruit sufficient participants to achieve the desired sample size meaning that a modest benefit of higher dose corticosteroids could not be detected and the statistical power to detect effect modification is very limited. Some participants in the lower-dose group received more than 6 mg dexamethasone daily, although very few received as much as 20 mg daily. This randomised trial is open label (i.e., participants and local hospital staff were aware of the assigned treatment). It is possible that the decision to offer invasive mechanical ventilation to those on non-invasive mechanical ventilation at baseline was influenced by knowledge of treatment allocation, although similar proportions in each group did receive such treatment. The primary outcome of death is unambiguous which minimized reporting bias for this, although other outcomes may have been susceptible to this. Information on radiological, virological or physiological outcomes were not collected.

In summary, the results provide no evidence that in hypoxic COVID-19 patients receiving ventilatory support, a higher dose of corticosteroids (dexamethasone 20 mg once daily for 5 days followed by 10 mg once daily for 5 days or until discharge if sooner) provides additional benefit over low dose corticosteroids.

## Contributors

This manuscript was initially drafted by RH, further developed by the Writing Committee, and approved by all members of the Trial Steering Committee. PWH and MJL vouch for the data and analyses, and for the fidelity of this report to the study protocol and data analysis plan. PWH, BB, RLH, JA, JKB, MB, LCC, JD, SNF, TJ, EJ, KJ, WSL, MM, AMo, AMu, KR, GT, RH, and MJL designed the trial and study protocol. MM, LP, MC, G P-A, EK, NTP, AS, NN, RS, PNT, DP, US, RS, EN, MR and the Data Linkage team at the RECOVERY Coordinating Centre, and the Health Records and Local Clinical Centre staff listed in the Supplementary Appendix collected the data. NS and JRE had access to the study data and did the statistical analysis. All authors contributed to data interpretation and critical review and revision of the manuscript. PWH and MJL had access to the study data and had final responsibility for the decision to submit for publication.

## Data sharing statement

The protocol, consent form, statistical analysis plan, definition & derivation of clinical characteristics & outcomes, training materials, regulatory documents, and other relevant study materials are available online at www.recoverytrial.net. As described in the protocol, the Trial Steering Committee will facilitate the use of the study data and approval will not be unreasonably withheld. Deidentified participant data will be made available to bona fide researchers registered with an appropriate institution within 3 months of publication. However, the Steering Committee will need to be satisfied that any proposed publication is of high quality, honours the commitments made to the study participants in the consent documentation and ethical approvals, and is compliant with relevant legal and regulatory requirements (e.g., relating to data protection and privacy). The Steering Committee will have the right to review and comment on any draft manuscripts prior to publication. Data will be made available in line with the policy and procedures described at: https://www.ndph.ox.ac.uk/data-access. Those wishing to request access should complete the form at https://www.ndph.ox.ac.uk/files/about/data_access_enquiry_form_13_6_2019.docx and e-mailed to: data.access@ndph.ox.ac.uk.

## Declaration of interests

The authors have no conflict of interest or individual financial relationships relevant to the submitted work to disclose. SF reports institutional support from Pfizer, Sanofi, GSK, J&J, Merck, AstraZeneca, Valneva, Moderna, Novavax, BioNTech and participation on a DMC or advisory board for AstraZeneca, Medimmune, Sanofi, Pfizer, Seqirus, Merck, J&J and MSD. MK reports institutional support from the National Institute for Health and Care Research. WSL reports institutional support from Pfizer and is chair of the UK Joint Committee on Vaccination and Immunisation. MM reports institutional support from Novaris, NovoNordisk and Health Data Research UK and participation on a DMC or steering committee for the National Institute for Health and Care Research. NS reports institutional support from Boehringer-Ingelheim, Eli Lilly and NovoNordisk. No form of payment was given to anyone to produce the manuscript. All authors have completed and submitted the ICMJE Form for Disclosure of Potential Conflicts of Interest. The Nuffield Department of Population Health at the University of Oxford has a staff policy of not accepting honoraria or consultancy fees directly or indirectly from industry (see https://www.ndph.ox.ac.uk/files/about/ndph-independence-of-research-policy-jun-20.pdf).

## Appendix A Supplementary data

Supplementary Appendix

RECOVERY DEX HDvent PubMEd Indexing list

RECOVERY Protocol
