# Supplementary material for: Higher dose corticosteroids in hospitalised COVID-19 patients requiring ventilatory support (RECOVERY): a randomised, controlled, open-label, platform trial
Source: eClinicalMedicine. 2025 Feb 12;81:103080. doi: 10.1016/j.eclinm.2025.103080 (PMC11872607; doi:10.1016/j.eclinm.2025.103080)
Supplement: RECOVERY DEX HDvent PubMEd Indexing list [file mmc2.docx]

**PubMed Indexing List - RECOVERY Collaborative Group – DEX HDvent paper 8 January 2025**

| **Initials** | **Surname** |
| --- | --- |
| O | Abani |
| A | Abbas |
| F | Abbas |
| J | Abbas |
| K | Abbas |
| M | Abbas |
| S | Abbasi |
| H | Abbass |
| A | Abbott |
| N | Abdallah |
| A | Abdelaziz |
| M | Abdelfattah |
| B | Abdelqader |
| A | Abdul |
| B | Abdul |
| S | Abdul |
| A | Abdul Rasheed |
| A | Abdulakeem |
| R | Abdul-Kadir |
| A | Abdullah |
| A | Abdulmumeen |
| R | Abdul-Raheem |
| N | Abdulshukkoor |
| K | Abdusamad |
| Y | Abed El Khaleq |
| M | Abedalla |
| A | Abeer Ul Amna |
| L | Abel |
| K | Abernethy |
| M | Abeywickrema |
| C | Abhinaya |
| A | Abidin |
| A | Aboaba |
| A | Aboagye-Odei |
| C | Aboah |
| H | Aboelela |
| H | Abo-Leyah |
| K | Abouelela |
| A | Abou-Haggar |
| M | Abouibrahim |
| A | Abousamra |
| M | Abouzaid |
| M | Abraham |
| T | Abraham |
| A | Abraheem |
| J | Abrams |
| R | Abrams |
| HJ | Abu |
| A | Abu-Arafeh |
| SM | Abubacker |
| A | Abung |
| Y | Abusamra |
| Y | Aceampong |
| A | Achara |
| D | Acharya |
| F | Acheampong |
| P | Acheampong |
| S | Acheampong |
| J | Acheson |
| S | Achieng |
| A | Acosta |
| R | Acquah |
| C | Acton |
| J | Adabie-Ankrah |
| P | Adair |
| AS | Adam |
| F | Adam |
| M | Adam |
| H | Adamali |
| M | Adamczyk |
| C | Adams |
| D | Adams |
| K | Adams |
| L | Adams |
| N | Adams |
| R | Adams |
| T | Adams |
| L | Adamu-Ikeme |
| K | Adatia |
| K | Adcock |
| L | Addai-Boampong |
| A | Addo |
| O | Adeagbo |
| A | Adebiyi |
| O | Adedeji |
| Y | Adegeye |
| K | Adegoke |
| V | Adell |
| S | Adenwalla |
| FW | Adeoye |
| OA | Adesemoye |
| EO | Adewunmi |
| A | Adeyanju |
| J | Adeyemi |
| T | Adeyemo |
| B | Adhikari |
| SA | Adhikari |
| R | Adhikary |
| A | Aditya |
| P | Adjepong |
| G | Adkins |
| A | Adnan |
| M | Adriaanse |
| J | Aeron-Thomas |
| D | Affleck |
| C | Afnan |
| M | Afridi |
| P | Afrim |
| FA | Afriyie |
| ZA | Aftab |
| A | Afum-Adjei Awuah |
| M | Agarwal |
| PN | Agasiya |
| R | Agbeko |
| C | Agbo |
| S | Aggarwal |
| A | Aghababaie |
| L | Aguilar Jimenez |
| JA | Agyekum |
| K | Agyen |
| EK | Ahadome |
| S | Ahamed Sadiq |
| MH | Ahammed Nazeer |
| M | Ahmad |
| S | Ahmad |
| A | Ahmed |
| BAR | Ahmed |
| B | Ahmed |
| F | Ahmed |
| H | Ahmed |
| I | Ahmed |
| K | Ahmed |
| L | Ahmed |
| M | Ahmed |
| MC | Ahmed |
| MS | Ahmed |
| N | Ahmed |
| N | Ahmed |
| O | Ahmed |
| RA | Ahmed |
| R | Ahmed |
| R | Ahmed |
| S | Ahmed |
| SG | Ahmed |
| SH | Ahmed |
| R | Ahmed Ali |
| B | Ahmed Mohamud |
| S | Ahmed |
| S | Ahmer |
| A | Ahonia |
| C | Aidoo |
| C | Aiken |
| D | Ail |
| M | Ainsworth |
| M | Aissa |
| L | Aitken |
| B | Ajay |
| A | Ajibode |
| A | Ajmi |
| N | Akhtar |
| S | Akili |
| B | Akinbiyi |
| O | Akindolie |
| Y | Akinfenwa |
| O | Akinkugbe |
| I | Akinpelu |
| M | Akram |
| O | Aktinade |
| U | Akudi |
| ASAR | Al Aaraj |
| A | Al Balushi |
| M | Al Dakhola |
| A | Al Swaifi |
| E | Al-Abadi |
| A | Alabi |
| N | Aladangady |
| M | Alafifi |
| A | Alam |
| S | Alam |
| A | Al-Asadi |
| K | Alatzoglou |
| P | Albert |
| A | Albertus |
| L | Albon |
| A | Alcala |
| G | Alcorn |
| S | Alcorn |
| A | Aldana |
| D | Alderdice |
| A | Aldesouki |
| R | Aldouri |
| J | Aldridge |
| N | Aldridge |
| R | Ale |
| RM | Ale |
| A | Alegria |
| A | Alexander |
| C | Alexander |
| J | Alexander |
| PDG | Alexander |
| J | Al-fori |
| L | Alghazawi |
| O | Alhabsha |
| B | Al-Hakim |
| R | Alhameed |
| M | Al-Hayali |
| S | Al-Hity |
| A | Ali |
| FR | Ali |
| J | Ali |
| M | Ali |
| MS | Ali |
| N | Ali |
| O | Ali |
| R | Ali |
| S | Ali |
| E | Aliberti |
| J | Alin |
| A | Alina |
| A | Alipustain |
| B | Alisjahbana |
| F | Aliyuda |
| K | Alizadeh |
| M | Al-Jibury |
| S | Al-Juboori |
| M | Al-Khalil |
| A | Alkhudhayri |
| M | Alkhusheh |
| F | Allan |
| N | Allan |
| A | Allanson |
| R | Allcock |
| E | Allen |
| J | Allen |
| K | Allen |
| L | Allen |
| P | Allen |
| R | Allen |
| S | Allen |
| T | Allen |
| A | Alli |
| K | Allison |
| B | Allman |
| HK | Allsop |
| L | Allsop |
| D | Allsup |
| AFT | Almahroos |
| H | Al-Moasseb |
| M | Al-Obaidi |
| L | Alomari |
| A | Al-Rabahi |
| B | Al-Ramadhani |
| Z | Al-Saadi |
| R | Al-Sammarraie |
| I | Alshaer |
| R | Al-Shahi Salman |
| W | Al-Shamkhani |
| F | Alsheikh |
| B | Al-Sheklly |
| S | Altaf |
| A | Alty |
| M | Alvarez |
| M | Alvarez Corral |
| E | Alveyn |
| M | Alzetani |
| S | Amamou |
| N | Amar |
| S | Ambalavanan |
| R | Ambrogetti |
| C | Ambrose |
| A | Ameen |
| A | Amelia Ganefianty |
| K | Ames |
| MR | Amezaga |
| A | Amin |
| A | Amin |
| K | Amin |
| S | Amin |
| T | Amin |
| B | Amit |
| A | Amjad |
| N | Amjad |
| J | Amoah-Dankwa |
| A | Amoako-Adusei |
| V | Amosun |
| M | Amsal |
| K | Amsha |
| J | Amuasi |
| N | Amutio Martin |
| P | Amy |
| A | Anada |
| A | Anand |
| S | Anandappa |
| SD | Anantapatnaikuni |
| NKN | Andari |
| E | Anderson |
| J | Anderson |
| L | Anderson |
| M | Anderson |
| N | Anderson |
| R | Anderson |
| S | Anderson |
| W | Anderson |
| P | Andreou |
| A | Andrews |
| J | Andrews |
| K | Aneke |
| A | Ang |
| WW | Ang |
| T | Angel |
| A | Angela |
| P | Angelini |
| L | Anguvaa |
| O | Anichtchik |
| M | Anim-Somuah |
| K | Aniruddhan |
| J | Annett |
| L | Anning |
| M | Ansah |
| PJ | Anstey |
| R | Anstey |
| A | Anthony |
| A | Anthony-Pillai |
| P | Antill |
| Z | Antonina |
| V | Anu |
| M | Anwar |
| S | Anwar |
| E | Apetri |
| A | Apostolopoulos |
| S | Appleby |
| D | Appleyard |
| MF | Aquino |
| B | Araba |
| S | Aransiola |
| M | Araujo |
| A | Archer |
| D | Archer |
| S | Archer |
| D | Arcoria |
| C | Ardley |
| G | Arhin-Sam |
| A-M | Arias |
| O | Aribike |
| R | Arimoto |
| NLPE | Arisanti |
| C | Arkley |
| C | Armah |
| I | Armata |
| J | Armistead |
| A | Armitage |
| C | Armstrong |
| M | Armstrong |
| S | Armstrong |
| W | Armstrong |
| P | Armtrong |
| H | Arndt |
| C | Arnison-Newgass |
| D | Arnold |
| R | Arnold |
| A | Arnott |
| D | Arora |
| K | Arora |
| P | Arora |
| R | Arora |
| A | Arter |
| A | Arthur |
| NM | Artini |
| A | Arumaithurai |
| A | Arya |
| R | Arya |
| D | Aryal |
| D | Asandei |
| GA | Asare |
| A | Asghar |
| M | Asghar |
| A | Ashab |
| C | Ashbrook-Raby |
| H | Ashby |
| J | Ashcroft |
| S | Ashcroft |
| G | Asher |
| Z | Ashfak |
| A | Ashfaq |
| HA | Asiamah |
| A | Ashish |
| D | Ashley |
| S | Ashman-Flavell |
| S | Ashok |
| A-E-A | Ashour |
| MZ | Ashraf |
| S | Ashraf |
| MB | Ashraq |
| D | Ashton |
| S | Ashton |
| A | Ashworth |
| FJ | Ashworth |
| R | Ashworth |
| A | Aslam |
| I | Aslam |
| S | Aslam |
| L | Aslett |
| H | Asogan |
| A | Asrar |
| O | Assaf |
| R | Astin-Chamberlain |
| YE | Atabudzi |
| P | Athavale |
| D | Athorne |
| B | Atkins |
| C | Atkins |
| S | Atkins |
| J | Atkinson |
| V | Atkinson |
| A | Atomode |
| B | Atraskiewicz |
| AA | Attia |
| E | Attubato |
| M | Attwood |
| P | Aubrey |
| Z | Auer |
| A | Aujayeb |
| AT | Aung |
| H | Aung |
| HWW | Aung |
| KK | Aung |
| KT | Aung |
| N | Aung |
| Y | Aung |
| ZM | Aung |
| E | Austin |
| K | Austin |
| A | Auwal |
| M | Avari |
| M | Avery |
| N | Aveyard |
| J | Avis |
| G | Aviss |
| C | Avram |
| P | Avram |
| A | Awadelkareem |
| G | Awadzi |
| M | Awaly |
| A | Awan |
| S | Awisi |
| A | Aya |
| E | Ayaz |
| JM | Ayerh |
| A | Ayers |
| J | Azam |
| A | Azeem |
| M | Azharuddin |
| A | Aziz |
| G | Aziz |
| I | Aziz |
| N | Aziz |
| A | Azkoul |
| A | Azman Shah |
| G | Azzopardi |
| H | Azzoug |
| F | Babatunde |
| M | Babi |
| B | Babiker |
| G | Babington |
| M | Babirecki |
| M | Babores |
| AO | Babs-Osibodu |
| T | Bac |
| S | Bacciarelli |
| R | Bachar |
| M-E | Bachour |
| A | Bachti |
| G | Bacon |
| J | Bacon |
| B | Badal |
| A | Badat |
| M | Bader |
| GR | Badhan |
| S | Badhrinarayanan |
| JP | Bae |
| A | Baggaley |
| A | Baggott |
| G | Bagley |
| D | Bagmane |
| L | Bagshaw |
| K | Bahadori |
| Y | Bahurupi |
| A | Bailey |
| J | Bailey |
| K | Bailey |
| L | Bailey |
| MA | Bailey |
| M | Bailey |
| P | Bailey |
| S | Bailey |
| H | Baillie |
| JK | Baillie |
| J | Bain |
| V | Bains |
| D | Baird |
| E | Baird |
| K | Baird |
| S | Baird |
| T | Baird |
| Y | Baird |
| A | Bajandouh |
| M | Bajracharya |
| DC | Baker |
| E | Baker |
| J | Baker |
| K | Baker |
| M | Baker |
| R | Baker |
| T-A | Baker |
| V | Baker |
| H | Bakere |
| N | Bakerly |
| M | Baker-Moffatt |
| A | Bakhai |
| N | Bakhtiar |
| P | Bakoulas |
| D | Bakthavatsalam |
| N | Balachandran |
| A | Balan |
| P | Balasingam |
| T | Balaskas |
| M | Balasubramaniam |
| N | Balatoni |
| A | Balcombe |
| A | Baldwin |
| A | Baldwin |
| C | Baldwin |
| D | Baldwin |
| F | Baldwin |
| R | Baldwin-Jones |
| N | Bale |
| J | Balfour |
| M | Ball |
| Ro | Ball |
| K | Ballard |
| I | Balluz |
| C | Balmforth |
| E | Balogh |
| A | Baltmr |
| A | Baluwala |
| G | Bambridge |
| A | Bamford |
| P | Bamford |
| A | Bamgboye |
| E | Bancroft |
| H | Bancroft |
| J | Banda |
| K | Bandaru |
| S | Bandi |
| N | Bandla |
| S | Bandyopadhyam |
| A | Banerjee |
| R | Banerjee |
| P | Bang |
| S | Baniya |
| O | Bani-Saad |
| H | Banks |
| L | Banks |
| P | Banks |
| C | Bann |
| H | Bannister |
| O | Bannister |
| L | Banton |
| DG | Bao |
| T | Bao |
| M | Baptist |
| T | Baqai |
| AM | Baral |
| SC | Baral |
| D | Baramova |
| R | Barber |
| E | Barbon |
| M | Barbosa |
| J | Barbour |
| A | Barclay |
| C | Barclay |
| G | Bardsley |
| S | Bareford |
| S | Bari |
| M | Barimbing |
| A | Barker |
| D | Barker |
| E | Barker |
| H | Barker |
| J | Barker |
| L | Barker |
| O | Barker |
| K | Barker-Williams |
| S | Barkha |
| J | Barla |
| G | Barlow |
| R | Barlow |
| V | Barlow |
| J | Barnacle |
| A | Barnard |
| D | Barnes |
| N | Barnes |
| R | Barnes |
| T | Barnes |
| C | Barnetson |
| A | Barnett |
| A | Barnett-Vanes |
| PG | Barning |
| W | Barnsley |
| A | Barr |
| D | Barr |
| J | Barr |
| C | Barr |
| N | Barratt |
| S | Barratt |
| M | Barrera |
| A | Barrett |
| Fi | Barrett |
| J | Barrett |
| S | Barrett |
| E | Barrow |
| J | Bartholomew |
| MS | Barthwal |
| C | Bartlett |
| G | Bartlett |
| J | Bartlett |
| L | Bartlett |
| S | Bartley |
| S | Bartolmeu-Pires |
| A | Barton |
| G | Barton |
| J | Barton |
| L | Barton |
| R | Barton |
| R | Baruah |
| S | Baryschpolec |
| H | Bashir |
| A | Bashyal |
| B | Basker |
| S | Basnet |
| B | Basnyat |
| A | Basoglu |
| A | Basran |
| J | Bassett |
| G | Bassett |
| C | Bassford |
| B | Bassoy |
| V | Bastion |
| A | Bastola |
| A | Basumatary |
| P | Basvi |
| JA | Batac |
| VR | Bataduwaarachchi |
| T | Bate |
| HJ | Bateman |
| K | Bateman |
| V | Bateman |
| E | Bates |
| H | Bates |
| M | Bates |
| S | Bates |
| S | Batham |
| A | Batista |
| A | Batla |
| D | Batra |
| H | Batty |
| T | Batty |
| A | Batty |
| M | Baum |
| R | Baumber |
| C | Bautista |
| F | Bawa |
| T | Bawa |
| FS | Bawani |
| S | Bax |
| M | Baxter |
| N | Baxter |
| Z | Baxter |
| H | Bayes |
| L-A | Bayo |
| F | Bazari |
| R | Bazaz |
| A | Bazli |
| L | Beacham |
| W | Beadles |
| K | Beadon |
| P | Beak |
| A | Beale |
| K | Beard |
| J | Bearpark |
| A | Beasley |
| S | Beattie |
| K | Beaumont |
| D | Beaumont-Jewell |
| T | Beaver |
| S | Beavis |
| C | Beazley |
| S | Beck |
| V | Beckett |
| R | Beckitt |
| S | Beckley |
| H | Beddall |
| S | Beddows |
| D | Beeby |
| S | Beeby |
| G | Beech |
| M | Beecroft |
| N | Beer |
| Sa | Beer |
| J | Beety |
| G | Bega |
| A | Begg |
| S | Begg |
| S | Beghini |
| A | Begum |
| S | Begum |
| S | Begum |
| T | Behan |
| R | Behrouzi |
| J | Beishon |
| C | Beith |
| J | Belcher |
| H | Belfield |
| K | Belfield |
| A | Belgaumkar |
| D | Bell |
| G | Bell |
| J | Bell |
| L | Bell |
| N | Bell |
| P | Bell |
| S | Bell |
| J | Bellamu |
| M | Bellamy |
| T | Bellamy |
| A | Bellini |
| A | Bellis |
| F | Bellis |
| L | Bendall |
| N | Benesh |
| N | Benetti |
| SA | Bengu |
| L | Benham |
| G | Benison-Horner |
| S | Benkenstein |
| T | Benn |
| A | Bennett |
| C | Bennett |
| D | Bennett |
| G | Bennett |
| K | Bennett |
| K | Bennett |
| L | Bennett |
| MR | Bennett |
| S | Bennett |
| K | Bennion |
| G | Benoy |
| V | Benson |
| A | Bentley |
| J | Bentley |
| I | Benton |
| E | Beranova |
| M | Beresford |
| C | Bergin |
| M | Bergstrom |
| J | Bernatoniene |
| T | Berriman |
| Z | Berry |
| F | Best |
| K | Best |
| A-M | Bester |
| Y | Beuvink |
| E | Bevan |
| S | Bevins |
| T | Bewick |
| A | Bexley |
| S | Beyatli |
| F | Beynon |
| A | Bhadi |
| S | Bhagani |
| S | Bhakta |
| R | Bhalla |
| K | Bhandal |
| K | Bhandal |
| A | Bhandari |
| L | Bhandari |
| LN | Bhandari |
| S | Bhandari |
| J | Bhanich Supapol |
| A | Bhanot |
| R | Bhanot |
| S | Bhasin |
| A | Bhat |
| P | Bhat |
| R | Bhatnagar |
| K | Bhatt |
| J | Bhayani |
| D | Bhojwani |
| P | Bhuie |
| MS | Bhuiyan |
| S | Bhuiyan |
| A | Bibby |
| F | Bibi |
| N | Bibi |
| S | Bibi |
| T | Bicanic |
| S | Bidgood |
| J | Bigg |
| S | Biggs |
| A | Biju |
| A | Bikov |
| S | Billingham |
| J | Billings |
| P | Binh |
| A | Binns |
| M | BinRofaie |
| O | Bintcliffe |
| C | Birch |
| J | Birch |
| K | Birchall |
| S | Bird |
| M | Birt |
| C | Bishop |
| K | Bishop |
| L | Bishop |
| K | Bisnauthsing |
| N | Biswas |
| M | Bittaye |
| S | Biuk |
| K | Blachford |
| E | Black |
| H | Black |
| K | Black |
| M | Black |
| P | Black |
| V | Black |
| H | Blackgrove |
| B | Blackledge |
| J | Blackler |
| S | Blackley |
| H | Blackman |
| C | Blackstock |
| C | Blair |
| F | Blakemore |
| H | Blamey |
| A | Bland |
| S | Blane |
| S | Blankley |
| P | Blaxill |
| K | Blaylock |
| J | Blazeby |
| N | Blencowe |
| B | Bloom |
| J | Bloomfield |
| A | Bloss |
| A | Blowers |
| S | Blows |
| H | Bloxham |
| S | Blrd |
| L | Blundell |
| A | Blunsum |
| M | Blunt |
| T | Blunt |
| I | Blyth |
| K | Blyth |
| A | Blythe |
| K | Blythe |
| KA | Boahen |
| M | Boampoaa |
| S | Board |
| E | Boatemah |
| B | Bobie |
| K | Bobruk |
| PN | Bodalia |
| N | Bodasing |
| M | Boden |
| T | Bodenham |
| G | Boehmer |
| M | Boffito |
| K | Bohanan |
| K | Bohmova |
| N | Bohnacker |
| S | Bokhandi |
| M | Bokhar |
| S | Bokhari |
| SO | Bokhari |
| I | Bokobza |
| A | Boles |
| C | Bolger |
| C | Bonaconsa |
| C | Bond |
| H | Bond |
| S | Bond |
| T | Bond |
| A | Bone |
| G | Boniface |
| J | Bonney |
| L | Bonney |
| L | Booker |
| S | Boot |
| M | Boothroyd |
| J | Borbone |
| N | Borman |
| S | Bosence |
| K | Bostock |
| N | Botting |
| F | Bottrill |
| H | Bouattia |
| L | Bough |
| H | Boughton |
| Z | Boult |
| T | Boumrah |
| M | Bourke |
| S | Bourke |
| M | Bourne |
| R | Bousfield |
| L | Boustred |
| A | Bowes |
| P | Bowker |
| T | Bowker |
| H | Bowler |
| L | Bowman |
| S | Bowman |
| R | Bowmer |
| A | Bowring |
| H | Bowyer |
| A | Boyd |
| J | Boyd |
| L | Boyd |
| N | Boyer |
| N | Boyle |
| P | Boyle |
| R | Boyle |
| L | Boyles |
| L | Brace |
| A | Bracken |
| J | Bradder |
| CJ | Bradley |
| P | Bradley |
| J | Bradley-Potts |
| L | Bradshaw |
| Z | Bradshaw |
| C | Brady |
| R | Brady |
| S | Brady |
| P | Braga Sardo |
| D | Braganza |
| M | Braithwaite |
| S | Brammer |
| M | Branch |
| T | Brankin-Frisby |
| J | Brannigan |
| S | Brattan |
| F | Bray |
| N | Bray |
| M | Brazil |
| L | Brear |
| Tr | Brear |
| S | Brearey |
| L | Bremner |
| M | Brend |
| C | Bresges |
| C | Bressington |
| G | Bretland |
| C | Brewer |
| M | Bridgett |
| G | Bridgwood |
| S | Brigham |
| J | Bright |
| C | Brightling |
| T | Brigstock |
| L | Brimfield |
| P | Brinksman |
| E | Brinkworth |
| R | Brittain-Long |
| V | Britten |
| H | Britton |
| L | Broad |
| S | Broadhead |
| R | Broadhurst |
| A | Broadley |
| M | Broadway |
| C | Brockelsby |
| M | Brocken |
| T | Brockley |
| M | Brodsky |
| F | Brogan |
| L | Brohan |
| F | Brokke |
| J | Brolly |
| D | Bromley |
| H | Brooke-Ball |
| V | Brooker |
| M | Brookes |
| D | Brooking |
| A | Brooks |
| D | Brooks |
| J | Brooks |
| K | Brooks |
| N | Brooks |
| P | Brooks |
| R | Brooks |
| S | Brooks |
| M | Broom |
| N | Broomhead |
| C | Broughton |
| N | Broughton |
| M | Brouns |
| A | Brown |
| C | Brown |
| E | Brown |
| H | Brown |
| J | Brown |
| L | Brown |
| N | Brown |
| P | Brown |
| R | Brown |
| S | Brown |
| T | Brown |
| B | Browne |
| C | Browne |
| D | Browne |
| M | Browne |
| S | Brownlee |
| A | Brraka |
| J | Bruce |
| M | Bruce |
| W | Brudlo |
| A | Brunchi |
| N | Brunskill |
| A | Brunton |
| M | Brunton |
| M | Bryant |
| E | Bryden |
| H | Brzezicki |
| A | Buazon |
| MH | Buch |
| R | Buchan |
| R | Buchanan |
| D | Buche |
| A | Buck |
| L | Buck |
| M | Buckland |
| C | Buckley |
| L | Buckley |
| P | Buckley |
| S | Buckley |
| C | Buckman |
| A | Budds |
| G | Bugg |
| R | Bujazia |
| M | Bukhari |
| S | Bukhari |
| R | Bulbulia |
| A | Bull |
| D | Bull |
| K | Bull |
| R | Bull |
| Th | Bull |
| E | Bullock |
| S | Bullock |
| N | Bulteel |
| K | Bumunarachchi |
| R | Bungue-Tuble |
| O | Burbidge |
| C | Burchett |
| D | Burda |
| C | Burden |
| TG | Burden |
| Mi | Burgess |
| R | Burgess |
| S | Burgess |
| E | Burhan |
| H | Burhan |
| H | Burke |
| K | Burke |
| A | Burman |
| S | Burnard |
| C | Burnett |
| S | Burnett |
| A | Burns |
| C | Burns |
| J | Burns |
| K | Burns |
| D | Burrage |
| K | Burrows |
| C | Burston |
| A | Burton |
| B | Burton |
| F | Burton |
| H | Burton |
| M | Burton |
| M | Butar butar |
| D | Butcher |
| A | Butler |
| E | Butler |
| J | Butler |
| P | Butler |
| S | Butler |
| J | Butler |
| A-T | Butt |
| M | Butt |
| MM | Butt |
| C | Butterworth |
| N | Butterworth-Cowin |
| R | Buttery |
| T | Buttle |
| H | Button |
| D | Buttress |
| H | Bye |
| J | Byrne |
| W | Byrne |
| V | Byrne-Watts |
| NK | C |
| A | Cabandugama |
| L | Cabrero |
| S | Caddy |
| R | Cade |
| A | Cadwgan |
| Z | Cahilog |
| A | Cahyareny |
| D | Cairney |
| J | Calderwood |
| D | Caldow |
| E | Cale |
| G | Calisti |
| D | Callaghan |
| J | Callaghan |
| C | Callens |
| D | Callum |
| C | Calver |
| M | Cambell-Kelly |
| T | Camburn |
| DR | Cameron |
| E | Cameron |
| F | Cameron |
| S | Cameron |
| C | Camm |
| FD | Cammack |
| A | Campbell |
| B | Campbell |
| D | Campbell |
| H | Campbell |
| J | Campbell |
| K | Campbell |
| M | Campbell |
| R | Campbell |
| W | Campbell |
| Q | Campbell Hewson |
| J | Camsooksai |
| L | Canclini |
| SM | Candido |
| J | Candlish |
| C | Caneja |
| A | Cann |
| J | Cann |
| R | Cannan |
| A | Cannon |
| E | Cannon |
| M | Cannon |
| P | Cannon |
| V | Cannons |
| E | Canonizado |
| J | Cantliff |
| N | Cap |
| NT | Cap |
| B | Caplin |
| S | Capocci |
| N | Caponi |
| A | Capp |
| R | Capstick |
| T | Capstick |
| C | Caraenache |
| A | Card |
| M | Cardwell |
| C | Carey |
| R | Carey |
| S | Carley |
| F | Carlin |
| T | Carlin |
| S | Carmichael |
| M | Carmody |
| M | Carnahan |
| C | Caroline |
| J | Carpenter |
| S | Carr |
| A | Carrasco |
| Z | Carrington |
| A | Carroll |
| P | Carroll |
| R | Carson |
| C | Cart |
| E | Carter |
| J | Carter |
| M | Carter |
| N | Carter |
| P | Carter |
| D | Cartwright |
| J-A | Cartwright |
| C | Carty |
| L | Carty |
| J | Carungcong |
| C | Carver |
| E | Carver |
| R | Carver |
| S | Casey |
| A | Cassells |
| T | Castiello |
| G | Castle |
| B | Castles |
| M | Caswell |
| AM | Catana |
| H | Cate |
| A | Catelan Zborowski |
| S | Cathcart |
| K | Cathie |
| D | Catibog |
| C | Catley |
| L | Catlow |
| M | Caudwell |
| A | Cavazza |
| A | Cave |
| L | Cave |
| S | Cavinato |
| F | Cawa |
| K | Cawley |
| C | Caws |
| K | Cawthorne |
| H | Cendl |
| H | Century |
| J | Cernova |
| M | Cesay |
| E | Cetti |
| S | Chabane |
| M | Chablani |
| C | Chabo |
| J | Chacko |
| D | Chadwick |
| J | Chadwick |
| R | Chadwick |
| E | Chakkarapani |
| A | Chakraborty |
| M | Chakraborty |
| M | Chakravorty |
| P | Chalakova |
| B | Chalise |
| BS | Chalise |
| J | Chalmers |
| R | Chalmers |
| G | Chamberlain |
| S | Chamberlain |
| E | Chambers |
| J | Chambers |
| L | Chambers |
| N | Chambers |
| A | Chan |
| C | Chan |
| E | Chan |
| M | Chan |
| K | Chan |
| P | Chan |
| R (P-C) | Chan |
| XHS | Chan |
| C | Chandler |
| H | Chandler |
| KJ | Chandler |
| S | Chandler |
| Z | Chandler |
| S | Chandra |
| N | Chandran |
| B | Chandrasekaran |
| Y | Chang |
| H | Chanh |
| HQ | Chanh |
| G | Chaplin |
| J | Chaplin |
| G | Chapman |
| J | Chapman |
| K | Chapman |
| L | Chapman |
| M | Chapman |
| P | Chapman |
| T | Chapman |
| L | Chappell |
| A | Charalambou |
| B | Charles |
| D | Charlton |
| S | Charlton |
| K | Chatar |
| C | Chatha |
| D | Chatterton |
| N | Chau |
| R | Chaube |
| A | Chaudhary |
| MYN | Chaudhary |
| B | Chaudhary |
| I | Chaudhry |
| Z | Chaudhry |
| K | Chaudhuri |
| N | Chaudhuri |
| M | Chaudhury |
| A | Chauhan |
| RS | Chauhan |
| L | Chaulagain |
| A | Chavasse |
| N | Chavasse |
| V | Chawla |
| L | Cheater |
| J | Cheaveau |
| C | Cheeld |
| M | Cheeseman |
| F | Chen |
| HM | Chen |
| T | Chen |
| F | Cheng |
| LY | Cheng |
| Z | Cheng |
| H | Chenoweth |
| CH | Cheong |
| JJ | Cherian |
| S | Cherian |
| M | Cherrie |
| H | Cheshire |
| CK | Cheung |
| E | Cheung |
| K | Cheung |
| M | Cheung |
| C | Cheyne |
| S | Chhabra |
| WL | Chia |
| E | Chiang |
| A | Chiapparino |
| R | Chicano |
| G | Chikara |
| M | Chikungwa |
| ZA | Chikwanha |
| G | Chilcott |
| S | Chilcott |
| A | Chilvers |
| P | Chimbo |
| KW | Chin |
| WJ | Chin |
| R | Chineka |
| A | Chingale |
| E | Chinonso |
| C | Chin-Saad |
| M | Chirgwin |
| H | Chisem |
| C | Chisenga |
| C | Chisholm |
| B | Chisnall |
| C | Chiswick |
| S | Chita |
| N | Chitalia |
| M | Chiu |
| L | Chiverton |
| B | Chivima |
| C | Chmiel |
| S | Choi |
| W | Choon Kon Yune |
| M | ​Chopra |
| V | Choudhary |
| O | Choudhury |
| S | Choudhury |
| B-L | Chow |
| M | Chowdhury |
| S | Chowdhury |
| A | Chrisopoulou |
| V | Christenssen |
| P | Christian |
| A | Christides |
| F | Christie |
| D | Christmas |
| G | Christoforou |
| T | Christopherson |
| A | Christou |
| M | Christy |
| P | Chrysostomou |
| Y | Chua |
| D | Chudgar |
| R | Chudleigh |
| S | Chukkambotla |
| ME | Chukwu |
| I | Chukwulobelu |
| CY | Chung |
| E | Church |
| SR | Church |
| D | Churchill |
| N | Cianci |
| P | Cicconi |
| P | Cinardo |
| Z | Cipinova |
| B | Cipriano |
| S | Clamp |
| B | Clancy |
| M | Clapham |
| E | Clare |
| S | Clare |
| A | Clark |
| C | Clark |
| D | Clark |
| E | Clark |
| F | Clark |
| G | Clark |
| J | Clark |
| K | Clark |
| L | Clark |
| M | Clark |
| N | Clark |
| P | Clark |
| R | Clark |
| T | Clark |
| Z | Clark |
| A | Clarke |
| J | Clarke |
| P | Clarke |
| R | Clarke |
| S | Clarke |
| A | Claxton |
| L | Claxton |
| K | Clay |
| C | Clayton |
| E | Clayton |
| O | Clayton |
| J | Clayton-Smith |
| B | Clearyb |
| C | Cleaver |
| R | Cleeton |
| I | Clement |
| C | Clemente de la Torre |
| J | Clements |
| S | Clements |
| S | Clenton |
| S | Cliff |
| R | Clifford |
| S | Clifford |
| A | Clive |
| J | Clouston |
| V | Clubb |
| S | Clueit |
| L | Clutterbuck |
| A | Clyne |
| M | Coakley |
| PGL | Coakley |
| K | Cobain |
| A | Cochrane |
| P | Cochrane |
| L | Cockayne |
| M | Cockerell |
| H | Cockerill |
| S | Cocks |
| R | Codling |
| A | Coe |
| S | Coetzee |
| D | Coey |
| D | Cohen |
| J | Cohen |
| O | Cohen |
| M | Cohn |
| L | Coke |
| O | Coker |
| N | Colbeck |
| R | Colbert |
| E | Cole |
| J | Cole |
| G | Coleman |
| M | Coleman |
| N | Coleman |
| H | Coles |
| M | Colin |
| A | Colino-Acevedo |
| J | Colley |
| K | Collie |
| A | Collier |
| D | Collier |
| H | Collier |
| T | Collingwood |
| P | Collini |
| E | Collins |
| J | Collins |
| K | Collins |
| M | Collins |
| N | Collins |
| S | Collins |
| V | Collins |
| A | Collinson |
| B | Collinson |
| J | Collinson |
| M | Collis |
| M | Colmar |
| HE | Colton |
| J | Colton |
| K | Colville |
| C | Colvin |
| E | Combes |
| D | Comer |
| A | Comerford |
| D | Concannon |
| A | Condliffe |
| R | Condliffe |
| E | Connell |
| L | Connell |
| N | Connell |
| K | Connelly |
| G | Connolly |
| E | Connor |
| A | Conroy |
| K | Conroy |
| V | Conteh |
| R | Convery |
| F | Conway |
| G | Conway |
| R | Conway |
| J-A | Conyngham |
| A | Cook |
| C | Cook |
| E | Cook |
| G | Cook |
| H | Cook |
| J | Cook |
| M | Cook |
| S | Cook |
| D | Cooke |
| G | Cooke |
| H | Cooke |
| J | Cooke |
| K | Cooke |
| T | Cooke |
| V | Cooke |
| A | Cooper |
| C | Cooper |
| D | Cooper |
| H | Cooper |
| J | Cooper |
| L | Cooper |
| N | Cooper |
| R | Cooper |
| S | Cooray |
| T | Cope |
| S | Corbet |
| C | Corbett |
| A | Corbishley |
| J | Corcoran |
| C | Cordell |
| J | Cordle |
| A | Corfield |
| J | Corless |
| A | Corlett |
| J | Cornwell |
| M | Cornwell |
| D | Corogeanu |
| A | Corr |
| M | Corredera |
| R | Corrigan |
| P | Corry |
| R | Corser |
| J | Cort |
| D | Cosgrove |
| T | Cosier |
| P | Costa |
| T | Costa |
| C | Coston |
| S | Cotgrove |
| Z | Coton |
| L-J | Cottam |
| R | Cotter |
| D | Cotterill |
| C | Cotton |
| G | Couch |
| M | Coulding |
| A | Coull |
| D | Counsell |
| D | Counter |
| C | Coupland |
| E | Courtney |
| J | Courtney |
| R | Cousins |
| AJ | Coutts |
| A | Cowan |
| E | Cowan |
| R | Cowan |
| R | Cowell |
| L | Cowen |
| S | Cowman |
| A | Cowton |
| E | Cox |
| G | Cox |
| H | Cox |
| K | Cox |
| M | Cox |
| K | Coy |
| A | Cradduck-Bamford |
| H | Craig |
| J | Craig |
| V | Craig |
| F | Craighead |
| M | Cramp |
| H | Cranston |
| SS | Crasta |
| J | Crause |
| A | Crawford |
| E | Crawford |
| I | Crawford |
| S | Crawshaw |
| B | Creagh-Brown |
| A | Creamer |
| A | Creaser-Myers |
| J | Cremona |
| S | Cremona |
| A | Crepet |
| J | Cresswell |
| M | Cribb |
| C | Crichton |
| D | Crilly |
| L | Crisp |
| N | Crisp |
| D | Crocombe |
| M | Croft |
| J | Crooks |
| H | Crosby |
| E | Cross |
| T | Cross |
| A | Crothers |
| S | Crotty |
| S | Crouch |
| M | Crow |
| A | Crowder |
| K | Crowley |
| T | Crowley |
| R | Croysdill |
| C | Cruickshank |
| I | Cruickshank |
| J | Cruise |
| C | Cruz |
| T | Cruz Cervera |
| D | Cryans |
| G | Cui |
| H | Cui |
| L | Cullen |
| G | Cummings-Fosong |
| V | Cunliffe |
| N | Cunningham |
| J | Cupitt |
| H | Curgenven |
| G | Curnow |
| D | Curran |
| S | Curran |
| C | Currie |
| J | Currie |
| S | Currie |
| J | Curtis |
| K | Curtis |
| M | Curtis |
| O | Curtis |
| T | Curtis |
| R | Cuthbertson |
| J | Cuthill |
| S | Cutler |
| S | Cutts |
| M | Czekaj |
| P | Czylok |
| S | D’Souza |
| J | da Rocha |
| GS | Dadzie |
| M | Dafalla |
| A | Dagens |
| H | Daggett |
| J | Daglish |
| S | Dahiya |
| A | Dale |
| K | Dale |
| M | Dale |
| S | Dale |
| J | Dales |
| U | D'Alessandro |
| H | Dalgleish |
| H | Dallow |
| C | D'aloia |
| D | Dalton |
| M | Dalton |
| Z | Daly |
| M | Damani |
| E | Damm |
| L | Dan |
| A | Danga |
| J | Dangerfield |
| A | Daniel |
| P | Daniel |
| A | Daniels |
| A | Dann |
| KG | Danso |
| S | Danso-Bamfo |
| QT | Dao |
| S | Darby |
| A | Darbyshire |
| J | Darbyshire |
| P | Dargan |
| P | Dark |
| K | Darlington |
| S | Darnell |
| T | Darton |
| G | Darylile |
| A | Das |
| M | Das |
| S | Das |
| M | Daschel |
| J | Dasgin |
| D | Datta |
| A | Daunt |
| V | Dave |
| E | Davenport |
| M | Davey |
| M | David |
| A | Davidson |
| L | Davidson |
| N | Davidson |
| R | Davidson |
| A | Davies |
| B | Davies |
| C | Davies |
| D | Davies |
| E | Davies |
| F | Davies |
| G | Davies |
| H | Davies |
| J | Davies |
| K | Davies |
| L | Davies |
| M | Davies |
| N | Davies |
| O | Davies |
| P | Davies |
| R | Davies |
| S | Davies |
| A | Davis |
| J-A | Davis |
| K | Davis |
| P | Davis |
| A | Davis-Cook |
| A | Davison |
| C | Dawe |
| H | Dawe |
| M | Dawkins |
| A | Dawson |
| D | Dawson |
| E | Dawson |
| J | Dawson |
| L | Dawson |
| M | Dawson |
| S | Dawson |
| T | Dawson |
| I | Dawson |
| A | Daxter |
| A | Day |
| J | Day |
| J | D'Costa |
| P | De |
| D | de Fonseka |
| T | de Freitas |
| P | De Los Santos Dominguez |
| R | De Pretto |
| F | De Santana Miranda |
| E | de Sausmarez |
| S | de Silva |
| T | de Silva |
| J | De Sousa |
| P | De Sousa |
| J | de Souza |
| P | De Souza |
| A | De Soyza |
| N | de Vere |
| J | de Vos |
| B | Deacon |
| S | Dealing |
| A | Dean |
| J | Dean |
| K | Dean |
| S | Dean |
| T | Dean |
| J | Deane |
| J | Dear |
| E | Dearden |
| C | Deas |
| S | Debbie |
| G | Debreceni |
| V | Deelchand |
| M | Deeley |
| J | Deery |
| E | Defever |
| M | Del Forno |
| A | Dela Rosa |
| G | De-La-Cedra |
| A | Dell |
| C | Demetriou |
| D | DeMets |
| J | Democratis |
| J | Denham |
| E | Denis |
| L | Denley |
| C | Denmade |
| A | Dent |
| K | Dent |
| M | Dent |
| E | Denton |
| T | Denwood |
| N | Deole |
| D | Depala |
| M | Depante |
| S | Dermody |
| A | Desai |
| P | Desai |
| S | Deshpande |
| V | Deshpande |
| S | Devkota |
| U | Devkota |
| D | Devonport |
| M | Devonport |
| P | Dey |
| V | Dey |
| R | Deylami |
| K | Dhaliwal |
| P | Dhangar |
| S | Dhani |
| A | Dhanoa |
| M | Dhar |
| A | Dhariwal |
| D | Dharmasena |
| D | Dhasmana |
| E | Dhillon |
| R | Dhillon |
| S | Dhillon |
| M | Dhimal |
| D | Dhiru |
| T | Dhorajiwala |
| P | Dias |
| S | Diaz |
| K | Diaz-Pratt |
| M | Dibas |
| D | Dickerson |
| P | Dicks |
| M | Dickson |
| S | Dickson |
| J | Digby |
| R | Digpal |
| S | Dillane |
| S | Diment |
| P | Dimitri |
| G | Dimitriadis |
| S | Din |
| TH | Dinh |
| TTT | Dinh |
| C | Dipheko |
| A | Dipper |
| S | Dipro |
| L | Dirmantaite |
| L | Dismore |
| L | Ditchfield |
| S | Diver |
| L | Diwakar |
| P | Diwan |
| C | Dixon |
| G | Dixon |
| K | Dixon |
| B | Djeugam |
| S | Dlamini |
| P | Dlouhy |
| A | D'Mello |
| P | Dmitri |
| T | Do |
| TT | Do |
| L | Dobbie |
| M | Dobranszky Oroian |
| C | Dobson |
| L | Dobson |
| M | Docherty |
| D | Dockrell |
| J | Dodd |
| J | Dodds |
| R | Dodds |
| S | Dodds |
| R | Dogra |
| C | Doherty |
| E | Doherty |
| W | Doherty |
| Y | Doi |
| I | Doig |
| E | Doke |
| D | Dolan |
| M | Dolman |
| R | Dolman |
| L | Donald |
| K | Donald |
| C | Donaldson |
| D | Donaldson |
| G | Donaldson |
| K | Donaldson |
| P | Dong |
| PK | Dong |
| M | Donkor |
| S | Donlon |
| J | Donnachie |
| E | Donnelly |
| R | Donnelly |
| P | Donnison |
| A | Donohoe |
| G | Donohoe |
| B | Donohue |
| E | Dooks |
| R | Doonan |
| R | Doorn |
| G | Doran |
| R | Dore |
| K | Dorey |
| S | Dorgan |
| K | Dos Santos |
| M | Dosani |
| D | Dosanjh |
| P | Dospinescu |
| I | Doss |
| T | Doudouliaki |
| A | Dougherty |
| ​J | ​Doughty |
| K | Douglas |
| J | Douse |
| A | Dow |
| L | Dowden |
| M | Dower |
| S | Dowling |
| N | Downer |
| C | Downes |
| R | Downes |
| T | Downes |
| D | Downey |
| R | Downey |
| C | Downing |
| L | Downs |
| S | Dowson |
| C | Dragan |
| C | Dragos |
| M | Drain |
| C | Drake |
| V | Drew |
| O | Drewett |
| A | Drexel |
| C | Driscoll |
| H | Drogan |
| O | Drosos |
| G | Drummond |
| K | Drury |
| K | Druryk |
| R | Druyeh |
| J | Dryburgh-Jones |
| S | Drysdale |
| P | Dsouza |
| A | Du Thinh |
| IK | Duah |
| H | Dube |
| J | Dube |
| S | Duberley |
| P | Duckenfield |
| H | Duckles-Leech |
| N | Duff |
| E | Duffield |
| H | Duffy |
| K | Duffy |
| L | Dufour |
| A | Duggan |
| P | Dugh |
| R | Duhoky |
| J | Duignan |
| J | Dulay |
| S | Dummer |
| A | Duncan |
| C | Duncan |
| F | Duncan |
| G | Duncan |
| H | Duncan |
| R | Duncan |
| S | Dundas |
| DV | Dung |
| NTP | Dung |
| A | Dunleavy |
| J | Dunleavy |
| A | Dunn |
| C | Dunn |
| D | Dunn |
| L | Dunn |
| P | Dunn |
| C | Dunne |
| K | Dunne |
| F | Dunning |
| A | Dunphy |
| T | Duong |
| TTH | Duong |
| V | Duraiswamy |
| B | Duran |
| I | DuRand |
| L | Durdle |
| N | Duric |
| A | Durie |
| E | Durie |
| S | Durogbola |
| C | Durojaiye |
| L | Durrans |
| K | Durrant |
| H | Durrington |
| I | Duru |
| H | Duvnjak |
| A | Dwarakanath |
| L | Dwarakanath |
| D | Dwomoh Nkrumah |
| E | Dwyer |
| Z | Dyar |
| C | Dyball |
| K | Dyer |
| H | Dymond |
| T | Dymond |
| ED | Dzidzomu |
| C | Eades |
| L | Eadie |
| R | Eadie |
| L | Eagles |
| B | Eapen |
| N | Earl |
| J | Early |
| M | Earwaker |
| N | Easom |
| C | East |
| A | Easthope |
| F | Easton |
| J | Easton |
| P | Easton |
| R | Eatough |
| O | Ebigbola |
| M | Ebon |
| A | Eccles |
| S | Eccles |
| C | Eddings |
| M | Eddleston |
| M | Edgar |
| K | Edgerley |
| N | Edmond |
| M | Edmondson |
| T | Edmunds |
| A | Edwards |
| C | Edwards |
| J | Edwards |
| K | Edwards |
| M | Edwards |
| S | Edwards |
| J | Eedle |
| A | Eggink |
| S | Eggleston |
| L | Ehiorobo |
| S | Eisen |
| M | Ekoi |
| A | Ekunola |
| N | Elashbar |
| L | Elawamy |
| D | Eleanor |
| S | El Behery |
| M | Elbeshy |
| K | El-Bouzidi |
| M | El-Din |
| E | Eldridge |
| U | Elenwa |
| I | Eletu |
| E | Elfar |
| M | Elgamal |
| A | Elgohary |
| N | Elkaram |
| R | Elmahdi |
| S | Eliammmknjhhhh |
| J | Elias |
| T | Elias |
| A | Elkins |
| J | Ellam |
| L | Ellerton |
| L | Elliot |
| A | Elliott |
| F | Elliott |
| K | Elliott |
| S | Elliott |
| A | Ellis |
| C | Ellis |
| K | Ellis |
| L | Ellis |
| R | Ellis |
| T | Ellis |
| T-Y | Ellis |
| Y | Ellis |
| A | Ellwood |
| R | Elmahdi |
| E | Elmahi |
| H-M | Elmasry |
| A | Emery |
| M | El-Naggar |
| N | Elndari |
| O | Elneima |
| M | Elokl |
| A | Elradi |
| M | Elsaadany |
| MASA | Elsayed |
| S | El-Sayeh |
| H | El-Sbahi |
| M | Elsebaei |
| T | Elsefi |
| K | El-Shakankery |
| A | Elsheikh |
| H | El-Taweel |
| S | Elyoussfi |
| J | Emberey |
| JR | Emberson |
| J | Emberton |
| A | Emery |
| J | Emmanuel |
| I | Emmerson |
| M | Emms |
| F | Emond |
| M | Emonts |
| N | Enachi |
| D | Enenche |
| A | Engden |
| K | English |
| C | Enimpah |
| E | Entwistle |
| H | Enyi |
| M | Erotocritou |
| P | Eskander |
| H | Esmail |
| F | Essa |
| B | Evans |
| C | Evans |
| D | Evans |
| E | Evans |
| G | Evans |
| I | Evans |
| J | Evans |
| L | Evans |
| M | Evans |
| R | Evans |
| S | Evans |
| T | Evans |
| C | Everden |
| S | Everden |
| L | Every |
| H | Evison |
| L | Evison |
| C | Ezenduka |
| J | Faccenda |
| L | Fahel |
| Y | Fahmay |
| I | Fairbairn |
| S | Fairbairn |
| T | Fairbairn |
| A | Fairclough |
| L | Fairlie |
| M | Fairweather |
| A | Fajardo |
| N | Falcone |
| E | Falconer |
| J | Fallon |
| A | Fallow |
| D | Faluyi |
| V | Fancois |
| A | Farah |
| M | Farah |
| Q | Farah |
| NZ | Fard |
| L | Fares |
| A | Farg |
| A | Farmer |
| K | Farmer |
| T | Farmery |
| S | Farnworth |
| F | Farook |
| H | Farooq |
| S | Farooq |
| F | Farquhar |
| H | Farr |
| A | Farrell |
| B | Farrell |
| F | Farrukh |
| J | Farthing |
| S | Farzana |
| R | Fasina |
| A | Fatemi |
| M | Fatemi |
| S | Fathima |
| N | Fatimah |
| M | Faulkner |
| S | Faust |
| C | Favager |
| A | Fawad |
| J | Fawke |
| S | Fawohunre |
| A | Fazal |
| A | Fazleen |
| S | Fearby |
| C | Fearnley |
| A | Feben |
| F | Fedel |
| D | Fedorova |
| C | Fegan |
| M | Felongo |
| L | Felton |
| T | Felton |
| K | Fenlon |
| A | Fenn |
| R | Fennelly |
| I | Fenner |
| C | Fenton |
| M | Fenton |
| G | Ferenando |
| C | Ferguson |
| J | Ferguson |
| K | Ferguson |
| S | Ferguson |
| V | Ferguson |
| D | Fernandes |
| C | Fernandez |
| E | Fernandez |
| M | Fernandez |
| S | Fernandez Lopez |
| J | Fernandez Roman |
| CJ | Fernando |
| J | Fernando |
| A | Feroz |
| P | Ferranti |
| T | Ferrari |
| E | Ferrelly |
| A | Ferrera |
| E | Ferriman |
| S | Ferron |
| N | Fethers |
| B | Field |
| J | Field |
| R | Field |
| K | Fielder |
| L | Fieldhouse |
| A | Fielding |
| J | Fielding |
| S | Fielding |
| A | Fikree |
| S | Filipa |
| S | Filson |
| S | Finan |
| S | Finbow |
| DJ | Finch |
| J | Finch |
| L | Finch |
| S | Finch |
| N | Fineman |
| J | Finlayson |
| L | Finlayson |
| A | Finn |
| J | Finn |
| D | Finnerty |
| C | Finney |
| D | Finucane |
| S | Fiouni |
| J | Fiquet |
| P | Firi |
| J | Fisher |
| N | Fisher |
| D | Fishman |
| K | Fishwick |
| C | Fitton |
| F | Fitzgerald |
| K | Fitzjohn |
| J | Flaherty |
| M | Flanagan |
| C | Flanders |
| N | Flaris |
| G | Fleming |
| J | Fleming |
| L | Fleming |
| P | Fleming |
| W | Flesher |
| A | Fletcher |
| J | Fletcher |
| L | Fletcher |
| S | Fletcher |
| F | Flett |
| K | Flewitt |
| S | Flockhart |
| C | Flood |
| I | Floodgate |
| J | Flor |
| V | Florence |
| M | Flowerdew |
| S | Floyd |
| MJ | Flynn |
| R | Flynn |
| C | Foden |
| A | Fofana |
| G | Fogarty |
| P | Foley |
| L | Folkes |
| T | Fong |
| DM | Font |
| A | Foo |
| J | Foo |
| A | Foot |
| HR | Foot |
| J | Foot |
| J | Forbes |
| A | Ford |
| J | Ford |
| I | Fordham |
| J | Foreman |
| M | Forester |
| M | Forkan |
| C | Fornolles |
| A | Forrest |
| E | Forsey |
| M | Forsey |
| T | Forshall |
| E | Forster |
| A | Forsyth |
| J | Forton |
| C | Foster |
| E | Foster |
| J | Foster |
| RA | Foster |
| T | Foster |
| A | Foulds |
| I | Foulds |
| F | Fowe |
| N | Fowkes |
| E | Fowler |
| R | Fowler |
| S | Fowler |
| A | Fox |
| C | Fox |
| H | Fox |
| J | Fox |
| L | Fox |
| L | Fox |
| N | Fox |
| O | Fox |
| S | Fox |
| S-J | Foxton |
| E | Fraile |
| R | Frake |
| A | Francioni |
| O | Francis |
| R | Francis |
| S | Francis |
| T | Francis-Bacon |
| H | Frankland |
| J | Franklin |
| S | Franklin |
| C | Fraser |
| A | Fratila |
| S | Frayling |
| M | Fredlund |
| A | Freeman |
| C | Freeman |
| E | Freeman |
| H | Freeman |
| N | Freeman |
| C | Freer |
| E | French |
| T | French |
| K | Freshwater |
| M | Frise |
| R | Fromson |
| A | Frosh |
| J | Frost |
| V | Frost |
| O | Froud |
| R | Frowd |
| A | Fryatt |
| A | Frygier |
| B | Fuller |
| L | Fuller |
| T | Fuller |
| D | Fullerton |
| C | Fung |
| G | Fung |
| S | Funnell |
| J | Furness |
| A | Fyfe |
| N | G |
| E | Gabbitas |
| C | Gabriel |
| Z | Gabriel |
| H | Gachi |
| S | Gaffarena |
| S | Gage |
| J | Gahir |
| S | Gajebasia |
| K | Gajewska-Knapik |
| B | Gajmer |
| Z | Galani |
| C | Gale |
| H | Gale |
| L | Gale |
| R | Gale |
| S | Gali |
| B | Gallagher |
| J | Gallagher |
| R | Gallagher |
| W | Gallagher |
| F | Gallam |
| J | Galliford |
| C | Galloway |
| E | Galloway |
| J | Galloway |
| A | Galvin |
| V | Galvis |
| G | Gamble |
| L | Gamble |
| B | Gammon |
| CN | Gan |
| MB | Ganaie |
| J | Ganapathi |
| R | Ganapathy |
| K | Gandhi |
| S | Gandhi |
| U | Ganesh |
| T | Ganeshanathan |
| S | Ganguly |
| A | Gani |
| P | Ganley |
| U | Garcia |
| E-J | Garden |
| AD | Gardener |
| E | Gardiner |
| M | Gardiner |
| P | Gardiner |
| S | Gardiner |
| C | Gardiner-Hill |
| J | Gardner |
| L | Gardner |
| M | Garfield |
| A | Garg |
| I | Garg |
| N | Garlick |
| D | Garner |
| J | Garner |
| L | Garner |
| Z | Garner |
| R | Garr |
| KA | Garrero |
| M | Gartaula |
| F | Garty |
| R | Gascoyne |
| H | Gashau |
| A | Gatenby |
| E | Gaughan |
| A | Gaurav |
| M | Gavrila |
| J | Gaylard |
| S | Gayle |
| C | Geddie |
| I | Gedge |
| S | Gee |
| F | Geele |
| K | Geerthan |
| M | Gellamucho |
| K | Gelly |
| L | Gelmon |
| S | Gelves-Zapata |
| G | Genato |
| N | Gent |
| S | Gent |
| N | Geoghegan |
| A | George |
| B | George |
| S | George |
| T | George |
| VP | George |
| S | Georges |
| D | Georgiou |
| P | Gerard |
| L | Gerdes |
| L | Germain |
| H | Gerrish |
| A | Getachew |
| L | Gethin |
| S | Gettings |
| H | Ghanayem |
| B | Ghavami Kia |
| S | Ghazal |
| A | Gherman |
| A | Ghosh |
| D | Ghosh |
| J | Ghosh |
| S | Ghosh |
| T | Giang |
| TV | Giang |
| S | Giannopoulou |
| M | Gibani |
| C | Gibb |
| B | Gibbison |
| K | Gibbons |
| A | Gibson |
| B | Gibson |
| J | Gibson |
| K | Gibson |
| S | Gibson |
| M | Gigi |
| C | Gilbert |
| J | Gilbert |
| K | Gilbert |
| B | Giles |
| J | Gilham |
| M | Gill |
| L | Gill |
| P | Gillen |
| A | Gillesen |
| K | Gillespie |
| E | Gillham |
| A | Gillian |
| D | Gilliland |
| R | Gillott |
| D | Gilmour |
| K | Gilmour |
| L | Gilmour |
| L | Ginn |
| F | Ginting |
| T | Giokanini-Royal |
| A | Gipson |
| B | Giri |
| J | Girling |
| R | Gisby |
| A | Gkioni |
| A | Gkoritsa |
| E | Gkrania-Klotsas |
| A | Gladwell |
| J | Glanville |
| J | Glasgow |
| S | Glasgow |
| J | Glass |
| L | Glass |
| S | Glaysher |
| L | Gledhill |
| E | Glenday |
| A | Glennon |
| J | Glossop |
| J | Glover |
| K | Glover |
| M | Glover |
| J | Glover Bengtsson |
| D | Glowski |
| S | Glynn |
| C | Gnanalingam |
| J | Goddard |
| W | Goddard |
| E | Godden |
| J | Godden |
| S | Godlee |
| E | Godson |
| G | Godwin |
| S | Gogoi |
| A | Goh |
| M | Gohel |
| R | Goiriz |
| S | Gokaraju |
| R | Goldacre |
| A | Goldsmith |
| P | Goldsmith |
| D | Gomersall |
| L | Gomez |
| R | Gomez-Marcos |
| A | Gondal |
| C | Gonzalez |
| J | Goodall |
| V | Goodall |
| B | Goodenough |
| A | Goodfellow |
| L | Goodfellow |
| J | Goodlife |
| C | Goodwin |
| E | Goodwin |
| J | Goodwin |
| P | Goodyear |
| R | Gooentilleke |
| M | Goonasekara |
| S | Gooseman |
| S | Gopal |
| C | Gordon |
| S | Gordon |
| R | Gore |
| H | Gorick |
| C | Gorman |
| S | Gormely |
| M | Gorniok |
| D | Gorog |
| M | Gorst |
| T | Gorsuch |
| J | Gosai |
| R | Gosling |
| S | Gosling |
| G | Gosney |
| V | Goss |
| D | Gotham |
| N | Gott |
| E | Goudie |
| N | Gould |
| S | Gould |
| C | Goumalatsou |
| L | Gourbault |
| A | Govind |
| R | Govindan |
| S | Gowans |
| G | Gowda |
| R | Gowda |
| H | Gower |
| P | Goyal |
| S | Goyal |
| C | Graham |
| J | Graham |
| L | Graham |
| R | Graham |
| S | Graham |
| M | Graham-Brown |
| J | Grahamslaw |
| G | Grana |
| T | Grandison |
| L | Grandjean |
| A | Grant |
| D | Grant |
| K | Grant |
| M | Grant |
| P | Grant |
| R | Gravell |
| J | Graves |
| A | Gray |
| C | Gray |
| G | Gray |
| J | Gray |
| K | Gray |
| N | Gray |
| R | Gray |
| S | Gray |
| A | Grayson |
| F | Greaves |
| P | Greaves |
| A | Green |
| AS | Green |
| C | Green |
| CA | Green |
| D | Green |
| F | Green |
| J | Green |
| M | Green |
| N | Green |
| S | Green |
| D | Greene |
| P | Greenfield |
| A | Greenhalgh |
| D | Greenwood |
| S | Greer |
| J | Gregory |
| K | Gregory |
| T | Gregory |
| J | Greig |
| R | Grenfell |
| T | Grenier |
| J | Grenville |
| J | Gresty |
| S | Grevatt |
| G | Grey |
| S | Gribben |
| A | Gribbin |
| A | Gribble |
| N | Grieg |
| D | Grieve |
| B | Griffin |
| D | Griffin |
| M | Griffin |
| S | Griffith |
| A | Griffiths |
| D | Griffiths |
| I | Griffiths |
| M | Griffiths |
| N | Griffiths |
| O | Griffiths |
| S | Griffiths |
| Y | Griffiths |
| S | Grigoriadou |
| S | Grigsby |
| P | Grist |
| E | Grobovaite |
| D | Grogono |
| C | Grondin |
| R | Groome |
| P | Grose |
| L | Grosu |
| J | Grounds |
| M | Grout |
| H | Grover |
| J | Groves |
| N | Grubb |
| J | Grundy |
| F | Guarino |
| S | Gudur |
| J | Guerin |
| S | Guettari |
| S | Gulati |
| V | Gulia |
| H | Gunasekara |
| P | Gunasekera |
| M | Gunawardena |
| K | Gunganah |
| J | Gunn |
| E | Gunter |
| A | Gupta |
| AK | Gupta |
| A | Gupta |
| R | Gupta |
| T | Gupta |
| V | Gupta |
| A | Gupta-Wright |
| V | Guratsky |
| A | Gureviciute |
| S | Gurram |
| A | Gurung |
| B | Gurung |
| L | Gurung |
| S | Gurung |
| S | Gurung Rai |
| H | Guth |
| N | Guthrine |
| S | Gyambrah |
| P | Gyanwali |
| S | Gyawali |
| N | Ha |
| NT | Ha |
| NX | Ha |
| R | Habibi |
| B | Hack |
| J | Hackett |
| P | Hackney |
| C | Hacon |
| A | Haddad |
| D | Hadfield |
| N | Hadfield |
| S | Hadfield |
| M | Hadjiandreou |
| N | Hadjisavvas |
| A | Haestier |
| N | Hafiz |
| R | Hafiz-Ur-Rehman |
| J | Hafsa |
| S | Hagan |
| JW | Hague |
| R | Hague |
| N | Haider |
| K | Haigh |
| V | Haile |
| J | Hailstone |
| C | Haines |
| S | Hainey |
| M | Hair |
| B | Hairsine |
| J | Hajnik |
| D | Hake |
| L | Hakeem |
| A | Haldeos |
| W | Halder |
| E | Hale |
| J | Hale |
| C | Halevy |
| P | Halford |
| W | Halford |
| A | Halim |
| A | Hall |
| C | Hall |
| E | Hall |
| F | Hall |
| H | Hall |
| J | Hall |
| K | Hall |
| L | Hall |
| J | Hallas |
| K | Hallas |
| C | Hallett |
| J | Halliday |
| A | Hallman |
| H | Halls |
| M | Hamdollah-Zadeh |
| IA | Hamed-Adekale |
| B | Hameed |
| M | Hameed |
| R | Hamers |
| I | Hamid |
| M | Hamie |
| R | Hamill |
| B | Hamilton |
| F | Hamilton |
| G | Hamilton |
| L | Hamilton |
| M | Hamilton |
| N | Hamilton |
| S | Hamilton |
| R | Hamlin |
| E | Hamlyn |
| B | Hammans |
| S | Hammersley |
| K | Hammerton |
| B | Hammond |
| E | Hammond |
| L | Hammond |
| S | Hammond |
| F | Hammonds |
| I | Hamoodi |
| K | Hampshire |
| JA | Hampson |
| J | Hampson |
| L | Hampson |
| L | Hamzah |
| J | Han |
| O | Hanci |
| S | Hand |
| L | Handayani |
| J | Handford |
| S | Handrean |
| NK | Handzewniak |
| S | Haney |
| DTT | Hang |
| V | Hang |
| VTK | Hang |
| D | Hanh |
| S | Hanif |
| E | Hanison |
| J | Hannah |
| A | Hannington |
| M | Hannun |
| A | Hanrath |
| H | Hanratty |
| D | Hansen |
| A | Hanson |
| H | Hanson |
| J | Hanson |
| K | Hanson |
| S | Hanson |
| N | Hao |
| A | Haqiqi |
| M | Haque |
| H | Harcourt |
| L | Harden |
| Z | Harding |
| S | Hardman |
| M | Hardwick |
| G | Hardy |
| J | Hardy |
| Y | Hardy |
| K | Haresh |
| R | Harford |
| B | Hargadon |
| J | Hargraves |
| C | Hargreaves |
| A | Harin |
| M | Haris |
| E | Harlock |
| P | Harman |
| T | Harman |
| M | Harmer |
| MA | Haroon |
| C | Harper |
| H | Harper |
| J | Harper |
| P | Harper |
| R | Harper |
| S | Harrhy |
| K | Harrington |
| S | Harrington |
| Y | Harrington-Davies |
| J | Harris |
| L | Harris |
| M-C | Harris |
| N | Harris |
| S | Harris |
| D | Harrison |
| L | Harrison |
| M | Harrison |
| OA | Harrison |
| R | Harrison |
| S | Harrison |
| T | Harrison |
| W | Harrison |
| E | Harrod |
| C | Hart |
| D | Hart |
| J | Hartley |
| L | Hartley |
| R | Hartley |
| T | Hartley |
| W | Hartrey |
| P | Hartridge |
| S | Hartshorn |
| A | Harvey |
| M | Harvey |
| C | Harwood |
| H | Harwood |
| Z | Harzeli |
| B | Haselden |
| H | Hasford |
| K | Hashem |
| M | Hashimm |
| T | Hashimoto |
| I | Hashmi |
| J | Haslam |
| Z | Haslam |
| G | Hasnip |
| A | Hassan |
| Z | Hassan |
| S | Hassasing |
| J | Hassell |
| P | Hassell |
| A | Hastings |
| B | Hastings |
| J | Hastings |
| S | Hathaway-Lees |
| J | Hatton |
| J | Hau |
| M | Havinden-Williams |
| S | Havlik |
| DB | Hawcutt |
| K | Hawes |
| L | Hawes |
| N | Hawes |
| L | Hawker |
| A | Hawkins |
| J | Hawkins |
| N | Hawkins |
| W | Hawkins |
| D | Hawley |
| E | Hawley-Jones |
| E | Haworth |
| AW | Hay |
| C | Hay |
| A | Hayat |
| J | Hayat |
| M-R | Hayathu |
| A | Hayes |
| J | Hayes |
| K | Hayes |
| M | Hayes |
| F | Hayes |
| P | Hayle |
| C | Haylett |
| A | Hayman |
| M | Hayman |
| M | Haynes |
| R | Haynes |
| R | Hayre |
| C | Hays |
| S | Haysom |
| J | Hayward |
| P | Haywood |
| H | Haywood Hasford |
| T | Hazelton |
| P | Hazenberg |
| Z | He |
| E | Headon |
| C | Heal |
| B | Healy |
| JL | Healy |
| A | Hearn |
| D | Heasman |
| A | Heath |
| D | Heath |
| R | Heath |
| D | Heaton |
| A | Heavens |
| K | Hebbron |
| C | Heckman |
| G | Hector |
| S | Heddon |
| A | Hedges |
| K | Hedges |
| C | Heeley |
| E | Heeney |
| R | Heinink |
| R | Heire |
| I | Helgesen |
| J | Hemingway |
| U | Hemmila |
| B | Hemmings |
| S | Hemphill |
| D | Hemsley |
| A | Henderson |
| E | Henderson |
| J | Henderson |
| S | Henderson |
| J | Henry |
| K | Henry |
| L | Henry |
| M | Henry |
| N | Henry |
| D | Henshall |
| G | Herdman |
| R | Herdman-Grant |
| M | Herkes |
| LE | Hermans |
| F | Hernandez |
| E | Heron |
| L | Heron |
| W | Herrington |
| E | Heselden |
| P | Heslop |
| T | Heslop |
| S | Hester |
| E | Hetherington |
| J | Hetherington |
| C | Hettiarachchi |
| P | Hettiarachchi |
| H | Hewer |
| J | Hewertson |
| A | Hewetson |
| S | Hewins |
| N | Hewitson |
| C | Hewitt |
| D | Hewitt |
| R | Hewitt |
| S | Hey |
| RS | Heyderman |
| M | Heydtmann |
| J | Heys |
| J | Heywood |
| M | Hibbert |
| J | Hickey |
| N | Hickey |
| P | Hickey |
| N | Hickman |
| A | Hicks |
| J | Hicks |
| S | Hicks |
| P | Hien |
| T | Hien |
| TT | Hien |
| D | Higbee |
| L | Higgins |
| A | Higham |
| M | Highcock |
| J | Highgate |
| M | Hikmat |
| A | Hill |
| H | Hill |
| J | Hill |
| L | Hill |
| P | Hill |
| U | Hill |
| A | Hilldrith |
| C | Hillman-Cooper |
| J | Hilton |
| Z | Hilton |
| S | Hinch |
| A | Hindle |
| E | Hindley |
| A | Hindmarsh |
| P | Hine |
| K | Hinshaw |
| C | Hird |
| C | Hirst |
| L | Hirst |
| J | Hives |
| HM | Hlaing |
| B | Ho |
| DKK | Ho |
| R | Ho |
| L | Hoa |
| LNM | Hoa |
| M | Hoare |
| D | Hobden |
| G | Hobden |
| M | Hobrok |
| S | Hobson |
| C | Hodge |
| S | Hodge |
| L | Hodgen |
| G | Hodgetts |
| H | Hodgkins |
| S | Hodgkinson |
| D | Hodgson |
| H | Hodgson |
| L | Hodgson |
| S | Hodgson |
| G | Hodkinson |
| K | Hodson |
| M | Hodson |
| A | Hogan |
| M | Hogben |
| L | Hogg |
| L | Hoggett |
| A | Holborow |
| C | Holbrook |
| R | Holbrook |
| C | Holden |
| J | ​Holden |
| M | Holden |
| S | Holden |
| T | Holder |
| N | Holdhof |
| H | Holdsworth |
| L | Holland |
| M | Holland |
| N | Holland |
| P | Holland |
| S | Holland |
| ML | Hollands |
| E | Holliday |
| K | Holliday |
| M | Holliday |
| N | Holling |
| L | Hollos |
| N | Hollos |
| L | Holloway |
| S | Holloway |
| M | Hollowday |
| M | Hollyer |
| A | Holman |
| A | Holmes |
| M | Holmes |
| R | Holmes |
| K | Holroyd |
| B | Holroyd-Hind |
| L | Holt |
| S | Holt |
| A | Holyome |
| M | Home |
| R | Homewood |
| K | Hong |
| L | Hoole |
| C | Hooper |
| S | Hope |
| B | Hopkins |
| PW | Horby |
| S | Horler |
| A | Hormis |
| D | Hornan |
| N | Hornby |
| T | Horne |
| Z | Horne |
| R | Horner |
| C | Horrobin |
| L | Horsford |
| M | Horsford |
| V | Horsham |
| A | Horsley |
| E | Horsley |
| S | Horton |
| J | Hosea |
| T | Hoskins |
| MS | Hossain |
| R | Hossain |
| M | Hough |
| S | Hough |
| C | Houghton |
| K | Houghton |
| R | Houlihan |
| K | Housely |
| H | Houston |
| R | Hovvels |
| L | How |
| L | Howaniec |
| J | Howard |
| K | Howard |
| L | Howard |
| M | Howard |
| S | Howard |
| R | Howard-Griffin |
| L | Howard-Sandy |
| S | Howe |
| A | Howell |
| M | Howells |
| L | Howie |
| K | Howlett |
| S | Howlett |
| R | Howman |
| J | Hrycaiczuk |
| H | Htet |
| NZ | Htoon |
| S | Htwe |
| Y | Hu |
| COH | Huah |
| S | Huang |
| K | Hubbard |
| A | Huckle |
| S | Huda |
| A | Hudak |
| L | Hudig |
| H | Hudson |
| S | Hudson |
| O | Hudson |
| A | Hufton |
| C | Huggins |
| A | Hughes |
| C | Hughes |
| E | Hughes |
| G | Hughes |
| H | Hughes |
| L | Hughes |
| M | Hughes |
| R | Hughes |
| S | Hughes |
| V | Hughes |
| W | Hughes |
| L | Huhn |
| C | Hui |
| R | Hulbert |
| D | Hull |
| G | Hull |
| R | Hull |
| A | Hulme |
| P | Hulme |
| W | Hulse |
| G | Hulston |
| R | Hum |
| M | Hume |
| C | Humphrey |
| A | Humphries |
| J | Humphries |
| T | Hung |
| C | Hunt |
| F | Hunt |
| J | Hunt |
| K | Hunt |
| L | Hunt |
| M | Hunt |
| S | Hunt |
| A | Hunter |
| C | Hunter |
| E | Hunter |
| K | Hunter |
| N | Hunter |
| R | Hunter |
| S | Hunter |
| G | Huntington |
| F | Huq |
| E | Hurditch |
| J | Hurdman |
| C | Hurley |
| K | Hurley |
| MA | Husain |
| S | Husaini |
| C | Huson |
| A | Hussain |
| C | Hussain |
| I | Hussain |
| M | Hussain |
| R | Hussain |
| S | Hussain |
| Y | Hussain |
| M | Hussam El-Din |
| SFEM | Hussein |
| Z | Hussein |
| R | Hussey |
| AH | Hussien |
| A | Hutchinson |
| C | Hutchinson |
| D | Hutchinson |
| E | Hutchinson |
| J | Hutchinson |
| C | Hutsby |
| P | Hutton |
| N | Huy |
| NQ | Huy |
| N | Huyen |
| NTT | Huyen |
| T | Huyen |
| TB | Huyen |
| NT | Huyen Thuong |
| H | Huynh |
| D | Hydes |
| J | Hyde-Wyatt |
| N | Hynes |
| M | Hyslop |
| A | Iakovou |
| K | Ibison |
| M | Ibraheim |
| A | Ibrahim |
| J | Ibrahim |
| M | Ibrahim |
| W | Ibrahim |
| B | Icke |
| AI | Idowu |
| M | Idrees |
| N | Idrees |
| H | Iftikhar |
| M | Iftikhar |
| C | Igwe |
| O | Igwe |
| M | Ijaz |
| A | Ikomi |
| C | Iles |
| S | Iliodromiti |
| M | Ilsley |
| L | Ilves |
| FM | Ilyas |
| L | Imam-Gutierrez |
| M | Iman |
| C | Imray |
| H | Imtiaz |
| J | Ingham |
| R | Ingham |
| T | Ingle |
| J | Inglis |
| S | Inglis-Hawkes |
| A | Ingram |
| L | Ingram |
| T | Ingram |
| N | Innes |
| P | Inns |
| V | Inpadhas |
| K | Inweregbu |
| AA | Ionescu |
| A | Ionita |
| I | Iordanov |
| A | Ipe |
| J | Iqbal |
| M | Iqbal |
| F | Iqbal Sait |
| I | Irabor |
| J | Irisari |
| R | Irons |
| M | Irshad |
| MS | Irshad |
| J | Irvine |
| V | Irvine |
| R | Irving |
| M | Ishak |
| E | Isherwood |
| G | Isitt |
| A | Islam |
| MDT | Islam |
| S | Islam |
| A | Ismail |
| O | Ismail |
| C | Ison |
| M | Israa |
| S | Isralls |
| H | Istiqomah |
| M | Ivan |
| C | Ivenso |
| N | Ivin |
| A | Ivy |
| S | Iwanikiw |
| K | Ixer |
| M | Iyer |
| A | Iakovou |
| K | Ibison |
| M | Ibraheim |
| A | Ibrahim |
| J | Ibrahim |
| M | Ibrahim |
| W | Ibrahim |
| B | Icke |
| AI | Idowu |
| M | Idrees |
| N | Idrees |
| H | Iftikhar |
| M | Iftikhar |
| C | Igwe |
| O | Igwe |
| M | Ijaz |
| A | Ikomi |
| C | Iles |
| S | Iliodromiti |
| M | Ilsley |
| L | Ilves |
| FM | Ilyas |
| L | Imam-Gutierrez |
| M | Iman |
| C | Imray |
| H | Imtiaz |
| J | Ingham |
| R | Ingham |
| T | Ingle |
| J | Inglis |
| S | Inglis-Hawkes |
| A | Ingram |
| L | Ingram |
| T | Ingram |
| N | Innes |
| P | Inns |
| V | Inpadhas |
| K | Inweregbu |
| AA | Ionescu |
| A | Ionita |
| I | Iordanov |
| A | Ipe |
| J | Iqbal |
| M | Iqbal |
| F | Iqbal Sait |
| I | Irabor |
| J | Irisari |
| R | Irons |
| M | Irshad |
| MS | Irshad |
| J | Irvine |
| V | Irvine |
| R | Irving |
| M | Ishak |
| E | Isherwood |
| G | Isitt |
| A | Islam |
| MDT | Islam |
| S | Islam |
| A | Ismail |
| O | Ismail |
| C | Ison |
| M | Israa |
| S | Isralls |
| H | Istiqomah |
| M | Ivan |
| C | Ivenso |
| N | Ivin |
| A | Ivy |
| S | Iwanikiw |
| K | Ixer |
| M | Iyer |
| K | Jabbar |
| C | Jack |
| J | Jackman |
| S | Jackman |
| A | Jackson |
| B | Jackson |
| E | Jackson |
| H | Jackson |
| L | Jackson |
| M | Jackson |
| N | Jackson |
| S | Jackson |
| Y | Jackson |
| J | Jacob |
| P | Jacob |
| R | Jacob |
| N | Jacques |
| H | Jadhav |
| A | Jafar |
| D | Jafferji |
| A | Jaffery |
| C | Jagadish |
| V | Jagannathan |
| A | Jagne |
| M | Jagpal |
| N | Jain |
| S | Jain |
| S | Jaiswal |
| D | Jajbhay |
| T | Jaki |
| P | Jali |
| B | Jallow |
| Y | Jaly |
| R | Jama |
| A | Jamal |
| S | Jamal |
| Z | Jamal |
| Y | Jameel |
| A | James |
| C | James |
| K | James |
| L | James |
| M | James |
| N | James |
| O | James |
| P | James |
| R | James |
| S | James |
| T | James |
| J | Jameson |
| L | Jamieson |
| A | Jamison |
| P | Jane |
| K | Janes |
| A | Janmohamed |
| D | Japp |
| P | Jaques |
| V | Jardim |
| C | Jardine |
| C | Jarman |
| E | Jarnell |
| E | Jarvie |
| C | Jarvis |
| R | Jarvis |
| P | Jastrzebska |
| H | Javed |
| A | Javier |
| M | Jawad |
| L | Jawaheer |
| A | Jayachandran |
| D | Jayachandran |
| A | Jayadev |
| A | Jayakumar |
| D | Jayaram |
| R | Jayaram |
| G | Jayasekera |
| T | Jayatilleke |
| A | Jayebalan |
| J | Jeater |
| S | Jeddi |
| V | Jeebun |
| MS | Jeelani |
| K | Jeffery |
| H | Jeffrey |
| R | Jeffrey |
| N | Jeffreys |
| B | Jeffs |
| C | Jeffs |
| JP | Jeganathan Ponraj |
| D | Jegede |
| T | Jemima |
| I | Jenkin |
| A | Jenkins |
| C | Jenkins |
| D | Jenkins |
| E | Jenkins |
| I | Jenkins |
| P | Jenkins |
| S | Jenkins |
| F | Jennings |
| J | Jennings |
| L | Jennings |
| V | Jennings |
| E | Jerome |
| D | Jerry |
| G | Jervis |
| E | Jessup-Dunton |
| J | Jesus Silva |
| C | Jetha |
| K | Jethwa |
| R | Jha |
| S | Jhanji |
| K | Jian |
| Z | Jiao |
| L | Jimenez |
| A | Jimenez Gil |
| J | Jith |
| T | Joefield |
| N | Johal |
| S | Johal |
| K | Johannessen |
| A | Johari |
| A | John |
| M | John |
| N | John |
| E | Johns |
| M | Johns |
| A | Johnson |
| E | Johnson |
| G | Johnson |
| K | Johnson |
| L | Johnson |
| M | Johnson |
| N | Johnson |
| O | Johnson |
| R | Johnson |
| B | Johnston |
| C | Johnston |
| J | Johnston |
| S | Johnston |
| V | Johnston |
| D | Johnstone |
| E | Johnstone |
| J | Johnstone |
| M | Joishy |
| A | Jones |
| B | Jones |
| C | Jones |
| CE | Jones |
| D | Jones |
| E | Jones |
| G | Jones |
| J | Jones |
| K | Jones |
| KE | Jones |
| L | Jones |
| LM | Jones |
| M | Jones |
| N | Jones |
| O | Jones |
| P | Jones |
| PH | Jones |
| R | Jones |
| RE | Jones |
| S | Jones |
| T | Jones |
| R | Jonnalagadda |
| R | Jordache |
| M | Jordan |
| S | Jordan |
| A | Jose |
| L | Jose |
| S | Jose |
| A | Joseph |
| G | Joseph |
| PA | Joseph |
| R | Joseph |
| S | Joseph |
| D | Joshi |
| M | Joshi |
| P | Joshi |
| T | Joshi |
| B | Josiah |
| DK | Joy |
| L | Joy |
| T | Joyce |
| H | Ju |
| A | Ju Wen Kwek |
| A | Judd |
| E | Jude |
| P | Judge |
| J | Juhl |
| S | Jujjavarapu |
| M | Juniper |
| E | Juszczak |
| D | Jyothish |
| K | Jabbar |
| C | Jack |
| J | Jackman |
| S | Jackman |
| A | Jackson |
| B | Jackson |
| E | Jackson |
| H | Jackson |
| L | Jackson |
| M | Jackson |
| N | Jackson |
| S | Jackson |
| Y | Jackson |
| J | Jacob |
| P | Jacob |
| R | Jacob |
| N | Jacques |
| A | Jafar |
| D | Jafferji |
| A | Jaffery |
| C | Jagadish |
| V | Jagannathan |
| A | Jagne |
| M | Jagpal |
| N | Jain |
| S | Jain |
| R | Jaiswal |
| S | Jaiswal |
| D | Jajbhay |
| T | Jaki |
| B | Jallow |
| Y | Jaly |
| R | Jama |
| A | Jamal |
| S | Jamal |
| Z | Jamal |
| Y | Jameel |
| A | James |
| C | James |
| K | James |
| L | James |
| M | James |
| N | James |
| O | James |
| P | James |
| R | James |
| S | James |
| T | James |
| J | Jameson |
| L | Jamieson |
| A | Jamison |
| P | Jane |
| K | Janes |
| A | Janmohamed |
| D | Japp |
| P | Jaques |
| V | Jardim |
| C | Jardine |
| C | Jarman |
| E | Jarnell |
| E | Jarvie |
| C | Jarvis |
| R | Jarvis |
| P | Jastrzebska |
| H | Javed |
| A | Javier |
| M | Jawad |
| L | Jawaheer |
| A | Jayachandran |
| D | Jayachandran |
| A | Jayadev |
| A | Jayakumar |
| D | Jayaram |
| R | Jayaram |
| G | Jayasekera |
| T | Jayatilleke |
| A | Jayebalan |
| J | Jeater |
| S | Jeddi |
| V | Jeebun |
| MS | Jeelani |
| K | Jeffery |
| H | Jeffrey |
| R | Jeffrey |
| N | Jeffreys |
| B | Jeffs |
| C | Jeffs |
| JP | Jeganathan Ponraj |
| D | Jegede |
| T | Jemima |
| I | Jenkin |
| A | Jenkins |
| C | Jenkins |
| D | Jenkins |
| E | Jenkins |
| I | Jenkins |
| P | Jenkins |
| S | Jenkins |
| F | Jennings |
| J | Jennings |
| L | Jennings |
| V | Jennings |
| E | Jerome |
| D | Jerry |
| G | Jervis |
| E | Jessup-Dunton |
| J | Jesus Silva |
| C | Jetha |
| K | Jethwa |
| R | Jha |
| RK | Jha |
| S | Jhanji |
| K | Jian |
| Z | Jiao |
| L | Jimenez |
| A | Jimenez Gil |
| J | Jith |
| T | Joefield |
| N | Johal |
| S | Johal |
| K | Johannessen |
| A | Johari |
| A | John |
| M | John |
| N | John |
| E | Johns |
| M | Johns |
| A | Johnson |
| E | Johnson |
| G | Johnson |
| K | Johnson |
| L | Johnson |
| M | Johnson |
| N | Johnson |
| O | Johnson |
| R | Johnson |
| B | Johnston |
| C | Johnston |
| J | Johnston |
| S | Johnston |
| V | Johnston |
| D | Johnstone |
| E | Johnstone |
| J | Johnstone |
| M | Joishy |
| A | Jones |
| B | Jones |
| C | Jones |
| CE | Jones |
| D | Jones |
| E | Jones |
| G | Jones |
| J | Jones |
| K | Jones |
| KE | Jones |
| L | Jones |
| LM | Jones |
| M | Jones |
| N | Jones |
| O | Jones |
| P | Jones |
| PH | Jones |
| R | Jones |
| RE | Jones |
| S | Jones |
| T | Jones |
| R | Jonnalagadda |
| R | Jordache |
| M | Jordan |
| S | Jordan |
| A | Jose |
| L | Jose |
| S | Jose |
| A | Joseph |
| G | Joseph |
| PA | Joseph |
| R | Joseph |
| S | Joseph |
| D | Joshi |
| M | Joshi |
| P | Joshi |
| T | Joshi |
| B | Josiah |
| L | Joy |
| T | Joyce |
| H | Ju |
| A | Ju Wen Kwek |
| A | Judd |
| E | Jude |
| P | Judge |
| J | Juhl |
| S | Jujjavarapu |
| M | Juniper |
| E | Juszczak |
| D | Jyothish |
| K | Kabiru Dawa |
| M | Kacar |
| D | Kadad |
| N | Kadam |
| N | Kader |
| A | Kailey |
| M | Kain |
| G | Kakoullis |
| A | Kakrani |
| A | Kala Bhushan |
| RJK | Kalayi |
| R | Kaliannan Periyasami |
| D | Kalita |
| I | Kalla |
| E | Kallistrou |
| T | Kalmus Eliasz |
| S | Kalsoom |
| E | Kam |
| J | Kamara |
| A | Kamath |
| P | Kamath |
| R | Kamath |
| SA | Kamerkar |
| N | Kametas |
| M | Kamfose |
| C | Kamundi |
| D | Kanabar |
| L | Kane |
| S | Kanitkar |
| O | Kankam |
| T | Kannan |
| A | Kant |
| V | Kapil |
| R | Kapoor |
| S | Kapoor |
| S | Kaprapina |
| S | Kar |
| J | Kara |
| E | Karbasi |
| S | Karelia |
| R | Kark |
| A | Karkey |
| A | Karki |
| S | Karki |
| S | Karmali |
| V | Karunanithi |
| N | Karunaratne |
| N | Kasianczuk |
| A | Kasiappan Balasubramanian |
| V | Kasipandian |
| R | Kassam |
| J | Kathirgamachelvam |
| M | Kati |
| V | Katsande |
| K | Kaul |
| D | Kaur |
| D | Kaur |
| J | Kaur |
| S | Kaur |
| Z | Kausar |
| L | Kavanagh |
| s | Kavanagh |
| MAA | Kawser |
| A | Kay |
| J | Kay |
| R | Kay |
| S | Kay |
| JN | Kayappurathu |
| S | Kayastha |
| C | Kaye |
| A | Kazeem |
| P | KC |
| M | Ke |
| T | Keady |
| R | Kearns |
| N | Kearsley |
| J | Keating |
| L | Keating |
| E | Keddie-Gray |
| B | Keegan |
| R | Keen |
| N | Keenan |
| J | Kefas |
| S | Kegg |
| L | Keith |
| U | Keke |
| J | Kellett |
| J | Kelliher |
| A | Kelly |
| D | Kelly |
| E | Kelly |
| H | Kelly |
| L | Kelly |
| M | Kelly |
| R | Kelly |
| S | Kelly |
| M | Kelly-Baxter |
| O | Kelsall |
| M | Keltos |
| T | Kemp |
| E | Kendall |
| A | Kendall-Smith |
| S | Kennard |
| A | Kennedy |
| J | Kennedy |
| M | Kennedy |
| S | Kennedy-Hay |
| J | Kenny |
| M | Kent |
| L | Keogan |
| A | Keough |
| D | Kernaghan |
| A | Kerr |
| C | Kerrison |
| A | Kerry |
| H | Kerslake |
| I | Kerslake |
| H | Kerss |
| J | Keshet-Price |
| E | Kestelyn |
| G | Keyte |
| A | Khadar |
| D | Khadka |
| NV | Khai |
| P | Khairunnisa |
| A | Khalid |
| H | Khalid |
| M | Khalid |
| MU | Khalid |
| S | Khalid |
| T | Khalifa |
| A | Khalil |
| S | Khalil |
| A | Khan |
| B | Khan |
| F | Khan |
| M | Khan |
| K | Khan |
| M | Khan |
| MA | Khan |
| N | Khan |
| O | Khan |
| R | Khan |
| S | Khan |
| T | Khan |
| W | Khan |
| Z | Khan |
| MS | Khan Tharin |
| NH | Khanh |
| U | Khatana |
| J | Khatri |
| H | Khatun |
| T | Khatun |
| M | Kheia |
| J | Khera |
| D | Khiem |
| DP | Khiem |
| HHE | Khin |
| TD | Khoa |
| N | Khoja |
| K | Khokhar |
| MQ | Khong |
| J | Khoo |
| VT | Khuong |
| C | Khurana |
| J | Kibaru |
| F | Kibutu |
| A | Kidd |
| M | Kidd |
| J | Kidney |
| S | Kidney |
| W | Kieffer |
| T | Kien |
| TV | Kien |
| J | Kilbane |
| C | Kilby |
| E | Killen |
| B | Kilner |
| S | Kilroy |
| B | Kim |
| JW | Kim |
| M | Kim |
| A | Kimber |
| S | Kimber |
| A | King |
| B | King |
| H | King |
| J | King |
| K | King |
| M | King |
| R | King |
| S | King |
| V | King |
| E | King-Oakley |
| L | Kingsmore |
| DJ | Kinnear |
| F | Kinney |
| S | Kiran |
| A | Kirby |
| A | Kirk |
| J | Kirk |
| A | Kirkby |
| E | Kirkham |
| G | Kirkman |
| L | Kirkpatrick |
| U | Kirwan |
| T | Kitching |
| L | Kitto |
| L | Kittridge |
| T | Kjoa |
| S | Klaczek |
| F | Kleemann |
| S | Kmachia |
| V | KN |
| CP | Knapp |
| L | Knibbs |
| A | Knight |
| F | Knight |
| M | Knight |
| S | Knight |
| T | Knight |
| E | Knights |
| J | Knights |
| M | Knolle |
| P | Knopp |
| C | Knowles |
| K | Knowles |
| L | Knowles |
| E | Knox |
| L | Knox |
| O | Koch |
| M | Kocsor |
| R | Kodituwakku |
| G | Koduri |
| YJ | Koe |
| J | Koirala |
| A | Koirata |
| E | Kolakaluri |
| M | Kolodziej |
| E | Kolokouri |
| S | Kon |
| N | Konar |
| M | Kononen |
| A | Konstantinidis |
| R | Kontogonis |
| H | Koo |
| I | Koopmans |
| E | Kopyj |
| L | Korcierz |
| J | Korolewicz |
| G | Koshy |
| C | Kosmidis |
| C | Kosztolanyi |
| J | Kotecha |
| E | Kothandaraman |
| R | Kothavale |
| K | Koukou |
| A | Kountourgioti |
| K | Kouranloo |
| R | Kousar´┐¢ |
| M | Kousteni |
| A | Koutalopoula |
| M | Kovac |
| A | Kozak Eskenazia |
| K | Krasauskas |
| R | Krishnamurthy |
| V | Krishnamurthy |
| M | Krishnan |
| H | Krishnan |
| N | Krishnapalli |
| S | Krizak |
| S | Krueper |
| S | Krupej |
| J | Krzowski |
| R | Kubaisi |
| S | Kubheka |
| A | Kubisz-Pudelko |
| S | Kuckreja |
| S | Kudsk-Iversen |
| A | Kudzinskas |
| C | Kukadiya |
| N | Kulkarni |
| S | Kumala Dewi |
| M | Kuma-Mintah |
| A | Kumar |
| G | Kumar |
| M | Kumar |
| R | Kumar |
| S | Kumar |
| V | Kumar |
| P | Kumar Panda |
| A | Kundu |
| H | Kunst |
| SS | Kunwar |
| K | Kupiec |
| A | Kurani |
| M | Kurdy |
| K | Kuriakose |
| R | Kurian |
| V | Kurmars |
| C | Kuronen-Stewart |
| RS | Kusangaya |
| V | Kushakovsky |
| A | Kutera |
| A | Kuverji |
| A | Kyei-Mensah |
| H | Kyepa |
| T | Kyere-Diabour |
| M | Kyi |
| NM | Kyi |
| L | Kyle |
| K-T | Kyriaki |
| J | Labao |
| L | Labuschagne |
| L | Lacey |
| N | Lack |
| M | Lacson |
| Z | Ladan |
| E | Ladlow |
| H | Lafferty |
| A | Lagnado |
| S | Laha |
| S | Lahane |
| C | Lai |
| J | Lai |
| P | Laidler |
| R | Laing |
| I | Laing-Faiers |
| E | Laity |
| K | Lake |
| N | Lakeman |
| D | Lalloo |
| F | Lalloo |
| A | Lam |
| C | Lam |
| F | Lamb |
| L | Lamb |
| T | Lamb |
| O | Lambert |
| P | Lambert |
| C | Lameirinhas |
| MKG | Lami |
| H | Lamont |
| M | Lamparski |
| D | Lamrani |
| C | Lanaghan |
| I | Lancona-Malcolm |
| G | Landers |
| MJ | Landray |
| M | Lane |
| N | Lane |
| A | Lang |
| S | Lang |
| D | Langer |
| M | Langley |
| C | Langoya |
| E | Langridge |
| E | Langthorne |
| H | Langton |
| B | Lara |
| T | Large |
| LN | Lartey |
| S | Lassa |
| A | Last |
| S | Latham |
| V | Latham |
| A | Latheef |
| L | Latif |
| N | Latt |
| C | Lau |
| D | Lau |
| E | Lau |
| GG | Laura |
| M | Laurenson |
| E | Lavington |
| H | Law |
| J | Law |
| KY | Law |
| P | Law |
| R | Law |
| L | Lawless |
| C | Lawrence |
| E | Lawrence |
| G | Lawrence |
| HM | Lawrence |
| N | Lawrence |
| R | Lawrie |
| L | Lawson |
| N | Lawson |
| R | Lawson |
| M | Lay |
| ​S | ​Laybourne |
| C | Laycock |
| R | Layug |
| M | Lazo |
| DH | Le |
| TT | Le |
| V | Le |
| A | Lea |
| W | Lea |
| LE | Leach |
| I | Leadbitter |
| T | Leahy |
| R | Lean |
| L | Leandro |
| D | Leaning |
| R | Leary |
| S | Leason |
| MA | Ledingham |
| C | Lee |
| E | Lee |
| G | Lee |
| H | Lee |
| I | Lee |
| J | Lee |
| S | Lee |
| SH | Lee |
| T | Lee |
| X | Lee |
| R | Lee |
| D | Lees |
| J | Lees |
| H | Legge |
| J | Leggett |
| K | Leigh-Ellis |
| D | Leitch |
| N | Leitch |
| E | Lekoudis |
| P | Lemessy |
| N | Lemoine |
| R | Lenagh |
| K | Leng |
| K | Lennon |
| L | Lennon |
| B | Leonard |
| K | Leonard |
| W | Leong |
| N | Leopold |
| O | Lepiarczyk |
| I | Leslie |
| EN | Lestari |
| E | Lester |
| E | Levell |
| C | Levett |
| A | Levynska |
| A | Lewin |
| A | Lewis |
| C | Lewis |
| D | Lewis |
| H | Lewis |
| J | Lewis |
| K | Lewis |
| L | Lewis |
| M | Lewis |
| N | Lewis |
| R | Lewis |
| C | Lewis-Clarke |
| A | Lewszuk |
| P | Lewthwaite |
| S | Ley |
| A | Liao |
| V | Licence |
| D | Lieberman |
| S | Liebeschuetz |
| T | Light |
| N | Lightfoot |
| P | Lillie |
| A | Lillis |
| B | Lim |
| C | Lim |
| ET | Lim |
| I | Lim |
| T | Lim |
| W | Lim |
| WS | Lim |
| J | Limb |
| D | Limbu |
| U | Limbu |
| C | Linares |
| D | Linden |
| G | Lindergard |
| K | Lindley |
| C | Lindsay |
| E | Lindsay |
| M | Lindsay |
| H | Lindsay- Clarke |
| M | Ling |
| C | Lingam |
| NVH | Linh |
| VD | Linh |
| L | Linkson |
| T | Linn |
| M | Linney |
| C | Lippold |
| G | Lipscomb |
| K | Lipscomb |
| L | Lipskis |
| A | Lisboa |
| E | Lister |
| J | Little |
| S | Little |
| L | Littlejohn |
| S | Liu |
| X | Liu |
| DK | Llanera |
| R | Llewellyn |
| M | Llewelyn |
| A | Lloyd |
| O | Lloyd |
| R | Lloyd |
| S | Lo |
| D | Loader |
| C | Loan |
| L | Lobosco |
| L | Lock |
| S | Lock |
| A | Locke |
| J | Locke |
| T | Locke |
| T | Lockett |
| J | Lodge |
| K | Lodhia |
| M | Lofthouse |
| H | Loftus |
| M | Logan |
| C | Logue |
| SY | Loh |
| S | Lokanathan |
| K | Lomme |
| E | London |
| G | Long |
| N | Long |
| K | Longbottom |
| B | Longhurst |
| M | Longshaw |
| S | Longstaffe |
| J | Lonnen |
| C | Lonsdale |
| L | Looby |
| R | Loosley |
| L | Lopes |
| P | Lopez |
| P | Lopez |
| RW | Lord |
| S | Lord |
| C | Lorimer |
| F | Loro |
| R | Lorusso |
| C | Loughlin |
| W | Lovegrove |
| R | Loveless |
| M | Lovell |
| A | Loverdou |
| A | Low |
| J | Low |
| S | Low |
| A | Lowe |
| C | Lowe |
| E | Lowe |
| F | Lowe |
| L | Lowe |
| M | Lowe |
| R | Lowsby |
| V | Lowthorpe |
| G | Lubimbi |
| A | Lubina Solomon |
| G | Lucas |
| J | Lucas |
| A | Lucey |
| O | Lucey |
| S | Luck |
| LH | Lui |
| A | Luintel |
| H | Luke |
| J | Luke |
| N | Lungu |
| A | Lunia |
| M | Lunn |
| J | Luo |
| M | Luscombe |
| J | Luveta |
| CN | Luximon |
| K | Lwin |
| M | Lwin |
| A | Lye |
| B | Lyell |
| E | Lyka |
| A | Lynas |
| C | Lynch |
| D | Lynch |
| S | Lynch |
| R-G | Maamari |
| H | Mabb |
| L | Mabelin |
| G | Mabeza |
| J | Macaro |
| K | Macconaill |
| C | Macdonald |
| A | Macduff |
| C | Macfadyen |
| JG | Macfarlane |
| J | Macfarlane |
| L | Macfarlane |
| I | Macharia |
| L | MacInnes |
| I | MacIntyre |
| J | MacIntyre |
| K | Mack |
| C | Mackay |
| E | Mackay |
| L | Mackay |
| A | Mackenzie |
| M | Mackenzie |
| R | MacKenzie Ross |
| A | Mackey |
| F | Mackie |
| J | Mackie |
| R | Mackie |
| C | Mackinlay |
| C | Mackintosh |
| K | Mackintosh |
| MJ | MacLeod |
| S | Macleod |
| M | Macmahon |
| A | MacNair |
| C | Macphee |
| I | Macpherson |
| C | Macrae |
| A | MacRaild |
| Y | Madani |
| A | Madden |
| M | Madden |
| C | Madden-McKee |
| S | Maddison |
| N | Madeja |
| P | Madhivathanan |
| M | Madhusudhana |
| A | Madu |
| L | Madziva |
| M | Mafham |
| S | Magar |
| N | Magee |
| F | Magezi |
| N | Maghsoodi |
| C | Magier |
| LM | Magnaye |
| M | Magriplis |
| M | Magtalas |
| NP | Magula |
| N | Mahabir |
| S | Mahadevan-Bava |
| S | Mahajan |
| A | Maharajh |
| K | Maharjan |
| M | Maharjan |
| A | Mahaveer |
| B | Mahay |
| K | Mahay |
| A | Mahdi |
| H | Mahdi |
| N | Mahdi |
| T | Mahendiran |
| S | Mahendran |
| S | Maher |
| A | Maheswaran |
| S | Maheswaran |
| T | Maheswaran |
| P | Mahjoob-Afag |
| A | Mahmood |
| F | Mahmood |
| H | Mahmood |
| W | Mahmood |
| Z | Mahmood |
| H | Mahmoud |
| M | Mahmud |
| E | Mahony |
| T | Mahungu |
| O | Maiga |
| L | Mair |
| T | Majekdunmi |
| K | Majid |
| A | Major |
| R | Major |
| J | Majumdar |
| MKH | Majumder |
| TLA | Mak |
| A | Makan |
| E | Makanju |
| S | Makin |
| W-O | Makinde |
| Y | Makkeyah |
| ON | Makoetlane |
| M | Malanca |
| H | Malcolm |
| F | Malein |
| N | Malhan |
| A | Malicka |
| A | Malik |
| G | Malik |
| M | Maljk |
| P | Mallett |
| P | Mallinder |
| G | Mallison |
| L | Mallon |
| E | Malone |
| G | Maloney |
| M | Mamman |
| I | Man |
| K | Man |
| R | Mancinelli |
| M | Mancuso-Marcello |
| S | Mandal |
| SK | Mandal |
| T | Manders |
| L | Manderson |
| J | Mandeville |
| T | Mane |
| R | Manhas |
| C | Maniero |
| R | Manikonda |
| I | Manjra |
| R | Mankiewitz |
| B | Mann |
| J | Manning |
| S | Manning |
| P | Mannion |
| K | Mansi |
| K | Manso |
| D | Mansour |
| M | Mansour |
| R | Mansour |
| IT | Mapfunde |
| P | Mappa |
| A | Maqsood |
| H | Maraj |
| C | Marchand |
| N | Marcus |
| A | Marcyniuk |
| M | Marecka |
| D | Maren |
| G | Margabanthu |
| J | Margalef |
| L | Margarit |
| G | Margaritopoulos |
| M | Margarson |
| F | Maria del Rocio |
| T | Maria Pfyl |
| V | Mariano |
| A | Maric |
| G | Markham |
| B | Marks |
| M | Marks |
| P | Marks |
| E | Marler |
| E | Marouzet |
| A | Marriott |
| C | Marriott |
| N | Marriott |
| C | Marsden |
| K | Marsden |
| P | Marsden |
| S | Marsden |
| T | Marsden |
| C | Marsh |
| G | Marsh |
| R | Marsh |
| A | Marshall |
| A | Marshall |
| G | Marshall |
| H | Marshall |
| J | Marshall |
| J | Marshall |
| N | Marshall |
| R | Marshall |
| S | Marshall |
| J | Marshall |
| E | Martin |
| G | Martin |
| H | Martin |
| J | Martin |
| K | Martin |
| L | Martin |
| M | Martin |
| N | Martin |
| T | Martin |
| W | Martin |
| S | Martin |
| T | Martindale |
| M | Martineau |
| L | Martinez |
| JC | Martinez Garrido |
| J | Martin-Lazaro |
| VK | Maruthamuthu |
| B | Marwan |
| G | Maryan |
| R | Mary-Genetu |
| S | Maryosh |
| V | Masani |
| A | Mascagni |
| D | Maseda |
| Z | Maseko |
| S | Mashate |
| Y | Mashhoudi |
| A | Mashta |
| I | Masih |
| S | Masih |
| N | Maskell |
| P | Maskell |
| P | Maskey |
| M | Masoli |
| J | Mason |
| R | Mason |
| C | Mason |
| M | Masood |
| MT | Masood |
| SSME | Masood |
| T | Massa |
| I | Massey |
| J | Masters |
| A | Masud |
| L | Matapure |
| C | Matei |
| R | Matewe |
| E | Matey |
| M | Matharu |
| S | Mathen |
| A | Mather |
| N | Mather |
| J | Mathers |
| J | Matheson |
| A | Mathew |
| A | Mathew |
| M | Mathew |
| V | Mathew |
| J | Mathews |
| K | Mathias |
| A | Mathioudakis |
| S | Matibela |
| D | Matila |
| W | Matimba-Mupaya |
| N | Matin |
| E | Matisa |
| E | Matkins |
| M | Matonhodze |
| E | Matovu |
| J | Mattappillil |
| AJ | Matthews |
| C | Matthews |
| H | Matthews |
| L | Mattocks |
| C | Maughan |
| TT | Maulidya |
| E | Mawson |
| F | Maxton |
| A | Maxwell |
| V | Maxwell |
| E | May |
| J | May |
| P | May |
| I | Mayanagao |
| M | Maycock |
| J | Mayer |
| G | Mayers |
| VA | Maynard |
| K | Mayne |
| T | Mayo |
| L | Mayola |
| S | Mayor |
| I | Mazen |
| T | Mazhani |
| A | Mazzella |
| N | Mburu |
| A | Mbuyisha |
| C | Mc Cague |
| E | McAleese |
| P | McAlinden |
| L | McAllister |
| A | McAlpine |
| G | McAlpine |
| J | McAndrew |
| H | McAuley |
| S | McAuliffe |
| C | McBrearty |
| E | McBride |
| M | McBuigan |
| J | McBurney |
| L | McCabe |
| GL | McCafferty |
| L | McCafferty |
| A | McCairn |
| J | McCammon |
| N | McCammon |
| C | McCann |
| E | McCann |
| A | McCarrick |
| B | McCarron |
| E | McCarthy |
| M | McCarthy |
| N | McCarthy |
| S | McCaughey |
| T | McClay |
| B | McClelland |
| D | McClintock |
| M | McCloskey |
| K | McCollum |
| A | McCorkindale |
| P | McCormack |
| J | McCormick |
| W | McCormick |
| P | McCourt |
| J | McCrae |
| S | McCready |
| G | McCreath |
| H | McCreedy |
| C | McCue |
| IJ | McCullagh |
| L | McCullagh |
| M | McCullagh |
| C | McCullough |
| K | McCullough |
| N | McCullough |
| S | McCullough |
| F | McCurrach |
| J | McDermott |
| P | McDermott |
| R | McDermott |
| K | McDevitt |
| H | McDill |
| B | McDonald |
| C | McDonald |
| D | McDonald |
| R | McDonald |
| S | McDonald |
| D | McDonald |
| N | McDonnell |
| C | McDougall |
| L | McDougall |
| R | McDougall |
| I | McEleavy |
| F | McElwaine |
| J | McEntee |
| E | McEvoy |
| C | McEwan |
| R | McEwen |
| M | McFadden |
| D | McFarland |
| M | McFarland |
| R | McFarland |
| J | McFlynn |
| E | McGarry |
| L | McGarvey |
| A | McGeachan |
| F | McGee |
| L | McGenily |
| C | McGettigan |
| M | McGettrick |
| C | McGhee |
| F | McGill |
| S | McGinnity |
| N | McGlinchey |
| P | McGlone |
| D | McGlynn |
| C | McGoldrick |
| E | McGough |
| C | McGovern |
| R | McGovern |
| A | McGowan |
| A | McGown |
| B | McGrath |
| A | McGregor |
| MP | McGuigan |
| H | McGuinness |
| S | McGuire |
| T | McHugh |
| C | McInnes |
| N | McInnes |
| J | McIntosh |
| K | McIntyre |
| M | McIntyre |
| L | McKay |
| CP | McKeag |
| J | McKeane |
| M | McKee |
| J | McKeever |
| J | McKenna |
| S | McKenna |
| M | McKenzie |
| D | McKeogh |
| C | McKerr |
| AM | McKie |
| H | Mckie |
| L | Mckie |
| G | McKnight |
| H | McLachlan |
| A | McLaren |
| B | McLaren |
| N | McLarty |
| D | Mclaughlan |
| M | McLaughlin |
| J | McLay |
| M | McLeish |
| T | McLennan |
| S | McLure |
| AM | McMahon |
| G | McMahon |
| M | McMahon |
| S | McMahon |
| T | McManus |
| M | McMaster |
| P | McMaster |
| P | McMaster |
| F | Mcmeeken |
| S | McMeekin |
| N | McMillan |
| K | McMillen |
| J | McMinn |
| L | McMorrow |
| H | McMullen |
| C | McMurran |
| H | McNally |
| F | McNeela |
| L | McNeil |
| C | McNeill |
| J | McNeill |
| S | McNeill |
| U | McNelis |
| M | McNulty |
| R | McNulty |
| C | McParland |
| M | McPhail |
| A | McQueen |
| A | McSkeane |
| D | McSorland |
| T | McSorley |
| G | McTaggart |
| J | McTaggart |
| J | Mead |
| P | Mead |
| E | Meadows |
| O | Meakin |
| B | Mearns |
| C | Mearns |
| K | Mears |
| W | Mears |
| M | Meda |
| A | Mediana |
| R | Medine |
| T | Medveczky |
| S | Meehan |
| E | Meeks |
| A | Megan |
| N | Meghani |
| S | Meghjee |
| S | Megson |
| A | Mehar |
| MN | Mehmood |
| R | Mehra |
| R | Mehta |
| G | Meintjes |
| J | Meirill |
| J | Meiring |
| R | Mejri |
| E | Mekonnen |
| S | Melander |
| A-S | Melinte |
| J | Mellersh |
| L | Melling |
| C | Mellish |
| F | Mellor |
| J | Mellor |
| S | Mellor |
| Z | Mellor |
| K | Mellows |
| V | Melnic |
| A | Melville |
| D | Melville |
| J | Melville |
| H | Membrey |
| M | Mencias |
| A | Mendelski |
| M | Mendelson |
| C | Mendonca |
| C | Meney |
| C | Menezes |
| W | Mensah |
| JE | Mensshan |
| A | Mentzer |
| D | Menzies |
| S | Menzies |
| S | Mepham |
| O | Mercer |
| P | Mercer |
| A | Merchant |
| F | Merchant |
| M | Mercioniu |
| M | Meredith |
| M | Merida Morillas |
| B | Merrick |
| J | Merritt |
| S | Merritt |
| P | Merron |
| E | Merwaha |
| S | Message |
| J | Messenger |
| G | Metcalf-Cuenca |
| A | Metcalfe |
| B | Metcalfe |
| K | Metcalfe |
| S | Metherell |
| A | Metryka |
| L | Mew |
| S | Meyrick |
| N | Mguni |
| J | Mhlongo |
| A | Miah |
| J | Miah |
| N | Miah |
| A | Mian |
| G | Mic |
| L | Micah-Amuah |
| D | Micallef |
| A | Michael |
| S | Michael |
| N | Michalak |
| L | Michalca-Mason |
| O | Michalec |
| J | Middle |
| H | Middleton |
| J | Middleton |
| M | Middleton |
| S | Middleton |
| S | Mieres |
| L | Mihalca-Mason |
| T | Mikolasch |
| S | Milgate |
| C | Millar |
| J | Millar |
| J | Millard |
| D | Miller |
| J | Miller |
| L | Miller |
| R | Miller |
| N | Miller-Biot |
| A | Miller-Fik |
| L | Millett |
| B | Milligan |
| H | Milligan |
| I | Milligan |
| C | Milliken |
| K | Millington |
| R | Millington |
| S | Millington |
| H | Mills |
| J | Mills |
| R | Mills |
| H | Millward |
| R | Miln |
| A | Milne |
| C | Milne |
| L | Milne |
| J | Milner |
| L | Milner |
| Z | Min |
| S | Mindel |
| N | Minh |
| PA | Minkah |
| C | Minnis |
| P | Minnis |
| K | Minou |
| N | Minskip |
| J | Minton |
| F | Miranda |
| M | Mirela |
| T | Mirza |
| A | Misbahuddin |
| A | Mishra |
| B | Mishra |
| E | Mishra |
| R | Mishra |
| S | Misra |
| D | Mistry |
| H | Mistry |
| D | Mital |
| S | Mitchard |
| B | Mitchell |
| C | Mitchell |
| LJ | Mitchell |
| P | Mitchell |
| P | Mitchelmore |
| A | Mitra |
| A | Mitra |
| S | Mitra |
| N | Mlambo |
| E | Moakes |
| K | Moar |
| E | Moatt |
| D | Mock Font |
| G | Modgil |
| A | Mohamed |
| A | Mohamed |
| O | Mohamed |
| A | Mohammad |
| W | Mohammad |
| A | Mohammed |
| O | Mohammed |
| YNS | Mohammed |
| B | Mohamud |
| A | Moharram |
| H-P | Mok |
| J | Mok |
| L | Mokogwu |
| M | Molina |
| C | Moller-Christensen |
| M | Mollet |
| M | Molloholli |
| A | Molloy |
| L | Molloy |
| A | Molyneux |
| R | Molyneux |
| T | Momoniat |
| H | Monaghan |
| K | Monaghan |
| S | Mongolu |
| T | Monika |
| K | Monsell |
| M | Montasser |
| A | Montgomery |
| H | Montgomery |
| P | Moodley |
| M | Moody |
| N | Moody |
| A | Moon |
| J | Moon |
| J-H | Moon |
| M | Moon |
| M | Moonan |
| P | Moondi |
| S | Moorby |
| J | Moorcroft |
| A | Moore |
| C | Moore |
| DAJ | Moore |
| F | Moore |
| J | Moore |
| L | Moore |
| N | Moore |
| S | Moore |
| V | Moore |
| R | Moores |
| E | Morab |
| J | Morales |
| N | Moramorell |
| L | Moran |
| G | Moray |
| J | Moreno-Cuesta |
| A | Morgan |
| C | Morgan |
| H | Morgan |
| K | Morgan |
| L | Morgan |
| M | Morgan |
| P | Morgan |
| K | Morgan-Jones |
| E | Morgan-Smith |
| J | Morilla |
| A | Morley |
| T | Morley |
| W | Morley |
| A | Morris |
| D | Morris |
| F | Morris |
| H | Morris |
| J | Morris |
| K | Morris |
| L | Morris |
| M-A | Morris |
| N | Morris |
| P | Morris |
| S | Morris |
| D | Morrison |
| M | Morrison |
| S | Morrison |
| M | Morrissey |
| AC | Morrow |
| A | Morrow |
| F | Morselli |
| G | Mortem |
| V | Mortland |
| C | Morton |
| G | Morton |
| P | Morzaria |
| D | Mosby |
| L | Moseley |
| K | Moshal |
| B | Moshy |
| A | Moss |
| C | Moss |
| J | Moss |
| S | Moss |
| O | Mostafa |
| G | Moth |
| N | Motherwell |
| S | Mottershaw |
| H | Moudgil |
| J | Mouland |
| C | Moulds |
| H | Moulton |
| G | Mounce |
| E | Mousley |
| C | Mowatt |
| K | Moxham |
| B | Moya |
| Q | Moyo |
| E | Mshengu |
| S | Mtuwa |
| A | Muazzam |
| IA | Muazzam |
| N | Muchenje |
| D | Mudawi |
| G | Muddegowda |
| R | Mufti |
| I | Mugal |
| A | Mughal |
| J | Muglu |
| F | Muhammad |
| J | Muhammad |
| C | Muir |
| A | Mukherjee |
| D | Mukherjee |
| J | Mukhtar |
| SAA | Mukhtar |
| D | Mukimbiri |
| J | Mulcahy |
| M | Mulcahy |
| P | Mulgrew |
| B | Mulhearn |
| A | Mulla |
| D | Mullan |
| D | Mullasseril Kutten |
| N | Mullen |
| R | Mullett |
| C | Mulligan |
| S | Mulligan |
| L | Mumelj |
| A | Mumford |
| M | Munavvar |
| H | Munby |
| H | Munday |
| A | Munro |
| S | Munt |
| M | Mupudzi |
| A | Murad |
| OH | Muraina |
| K | Muralidhara |
| M | Murdoch |
| J | Murira |
| A | Murphy |
| B | Murphy |
| C | Murphy |
| E | Murphy |
| G | Murphy |
| H | Murphy |
| P | Murphy |
| R | Murphy |
| S | Murphy |
| C | Murray |
| D | Murray |
| E | Murray |
| K | Murray |
| L | Murray |
| T | Murray |
| E | Murtagh |
| M | Murthy |
| C | Murton |
| R | Murton |
| N | Muru |
| R | Musanhu |
| M | Mushabe |
| O | Mushtaq |
| S | Musini |
| AMM | Mustafa |
| E | Mustafa |
| M | Mustafa |
| I | Mustapha |
| N | Mustfa |
| Z | Mustufvi |
| C | Mutch |
| R | Mutch |
| E | Mutema |
| B | Muthukrishnan |
| S | Mutton |
| N | Muzengi |
| M | Mwadeyi |
| B | Mwale |
| E | Mwaura |
| R | Myagerimath |
| A | Myers |
| S | Myers |
| JS | Myerson |
| K | Myint |
| Y | Myint |
| G | Mynott |
| L | Myslivecek |
| P | Nabayego |
| E | Nadar |
| I | Nadeem |
| M | Nadheem |
| B | Nadjm |
| A | Naeem |
| H | Naeem |
| S | Naeem |
| S | Nafees |
| M | Nafei |
| W | Naftalia |
| T | Nagarajan |
| L | Naglik |
| I | Nagra |
| D | Nagra |
| M | Naguib |
| K | Naguleswaran |
| KS | Nagumantry |
| K | Naicker |
| S | Naidoo |
| V | Naidoo |
| G | Naik |
| R | Naik |
| S | Naik |
| DS | Nair |
| R | Nair |
| T | Nair |
| J | Naisbitt |
| K | Naismith |
| D | Nakiboneka-Ssenabulya |
| S | Nallapareddy |
| S | Nallapeta |
| A | Nallasivan |
| H | Nam |
| U | Nanda |
| A | Nandani |
| T | Nandwani |
| AR | Naqvi |
| A | Naqvi |
| S | Naqvi |
| S | Nasa |
| D | Nash |
| N | Nasheed |
| A | Nasimudeen |
| U | Nasir |
| N | Nasronudin |
| T | Nasser |
| A | Natarajan |
| G | Natarajan |
| N | Natarajan |
| R | Natarajan |
| P | Nath |
| N | Nathaniel |
| M | Nathvani |
| P | Nathwani |
| G | Nava |
| N | Navaneetham |
| J | Navaratnam |
| H | Navarra |
| S | Naveed |
| J | Navin |
| K | Nawaz |
| S | Nawaz |
| B | Nayar |
| S | Naylor |
| M | Nayyar |
| F | Naz |
| M | Naz |
| B | Nazari |
| A | Nazir |
| S | Nazir |
| D | Ncomanzi |
| O | Ndefo |
| NB | Ndoumbe |
| A | Neal |
| E | Neary |
| M | Negmeldin |
| J | Neil |
| P | Neill |
| HE | Neils |
| A | Nejad |
| J | Nel |
| L | Nel |
| A | Nelson |
| B | Nelson |
| L | Nelson |
| M | Nelson |
| R | Nelson |
| S | Nelson |
| E | Nelwan |
| EJ | Nelwan |
| R | Nemane |
| S | Nepal |
| D | Nethercott |
| K | Netherton |
| K | Nettleton |
| J | Neupane |
| K | Neupane |
| A | Newby |
| D | Newby |
| T | Newcombe |
| H | Newell |
| C | Newman |
| D | Newman |
| H | Newman |
| J | Newman |
| O | Newman |
| T | Newman |
| R | Newport |
| M | Newton |
| AYKC | Ng |
| HEJ | Ng |
| KW | Ng |
| M | Ng |
| S | Ng |
| WJ | Ng |
| YWM | Ng |
| T | Ngan |
| TH | Ngo |
| GCE | Ngui |
| A | Ngumo |
| HK | Nguyen |
| MT | Nguyen |
| N | Nguyen |
| NT | Nguyen |
| NTT | Nguyen |
| Q | Nguyen |
| TH | Nguyen |
| THT | Nguyen |
| TT | Nguyen |
| TTN | Nguyen |
| TT | Nguyen |
| TTP | Nguyen |
| K | Ngwenya |
| NY | Nhi |
| C | Nic Fhogartaigh |
| N | Nicholas |
| P | Nicholas |
| R | Nicholas |
| D | Nicholls |
| L | Nicholls |
| S | Nicholls |
| A | Nicholson |
| I | Nickson |
| E | Nicol |
| R | Nicol |
| P | Nicola |
| A | Nicoll |
| T | Nightingale |
| F | Nikita |
| P | Nikolaos |
| G | Nikonovich |
| A | Nilsson |
| K | Nimako |
| L | Nimako |
| C | Nimmo |
| P | Ninan |
| T | Ninh |
| M | Nirmalan |
| R | Niroula |
| A | Nisar |
| M | Nisar |
| T | Nisar |
| T | Nisbett |
| A | Nisha James |
| S | Nishat |
| T | Nishiyama |
| S | Nix |
| J | Nixon |
| M | Nixon |
| K | Nizam Ud Din |
| M | Nizami |
| S | Nizamis |
| R | Njafuh |
| I | Noakes |
| L | Noba |
| J | Noble |
| H | Noble |
| HM | Noe |
| J | Nolan |
| J | Nolasco |
| Z | Noor |
| Z | Noori |
| J | Norcliffe |
| L | Norman |
| R | Norman |
| E | Norris |
| K | Norris |
| L | Norris |
| SA | Nortcliffe |
| F | North |
| J | North |
| T | North |
| J | Northfield |
| S | Northover |
| J | Nortje |
| D | Norton |
| R | Norton |
| H | Notman |
| K | Nourein |
| T | Novak |
| N | Novas Duarte |
| C | Novis |
| JA | Nowak |
| KP | Nu |
| M | Nugdallah |
| A | Nugent |
| J | Nugent |
| C | Nugroho |
| N | Numbere |
| K | Nundlall |
| A | Nune |
| K | Nunn |
| M | Nunn |
| J | Nunnick |
| Y | Nupa |
| F | Nur |
| Z | Nurgat |
| R | Nurpeni |
| A | Nuttall |
| L | Nwafor |
| P | Nwajiugo |
| G | Nyamugunduru |
| L | Nyanor |
| M | Nyirenda |
| K | Nyland |
| D | O Rinn |
| D | O Shea |
| M | O Toole |
| M | O’Hara |
| C | O’Hara |
| L | O’Keefe |
| K | O’Reilly |
| W | O’Rourke |
| C | Oakley |
| N | Oakley |
| S | Oakley |
| HTK | Oanh |
| B | Obale |
| C | Oboh |
| C | O'Brien |
| J | O'Brien |
| K | O'Brien |
| L | O'Brien |
| N | O'Brien |
| R | O'Brien |
| T | O'Brien |
| E | O'Bryan |
| R | Obukofe |
| C | O'Callaghan |
| L | O'Connell |
| T | OConnor |
| C | O'Connor |
| G | O'Connor |
| M | Odam |
| S | Oddie |
| S | Oddy |
| Y | Odedina |
| K | Odedra |
| S | Odelberg |
| N | Odell |
| O | Oderinde |
| J | Odone |
| L | O'Donohoe |
| C | O'Donovan |
| I | Odysseos-Beaumont |
| S | O'Farrell |
| P | Offord |
| M | O'Flaherty |
| E | Ofori |
| T | Ogbara |
| C | Ogilvie |
| C | O'Gorman |
| I | Ogunjembola |
| O | Ogunkeye |
| U | Ohia |
| S | Ojha |
| S | Ojha |
| O | Ojo |
| F | O'kane |
| M | O'Kane |
| T | Okeke |
| E | OKell |
| A | Okines |
| I | Okpala |
| E | Okpo |
| F | Okpoko |
| M | Okubanjo |
| C | Oladipo |
| L | Olaivar |
| R | Olaiya |
| J | Olatujoye |
| T | Old |
| G | Oleszkiewicz |
| A | Oliver |
| C | Oliver |
| J | Oliver |
| L | Oliver |
| M | Oliver |
| Z | Oliver |
| J | Oliver-Commey |
| NO | Olokoto |
| F | Olonipile |
| O | Olufuwa |
| O | Olukoya |
| A | Oluwole-Ojo |
| L | O'Malley |
| IV | Omale |
| PK | Omane-Donkor |
| M | Omar |
| Z | Omar |
| N | Omer |
| E | Omoregie |
| C | O'Neill |
| L | O'Neill |
| C | Ong |
| O | Onuoha |
| C | Onyeagor |
| CN | Oo |
| Z | Oo |
| HC | Ooi |
| SH | Ooi |
| A | Oomatia |
| A | Opata |
| M | Opena |
| R | Oram |
| C | Ord |
| J | Ord |
| C | Oreilly |
| L | Orekoya |
| D | O'Riordan |
| S | O'Riordan |
| I | Orlikowska |
| A | Orme |
| H | Orme |
| L | O'Rourke |
| C | Orr |
| S | Orr |
| C | Orton |
| A | Osadcow |
| R | Osagie |
| R | Osanlou |
| L | Osborne |
| N | Osborne |
| R | Osborne |
| W | Osborne |
| W | Osborne |
| C | Osbourne |
| J | Osei-Bobie |
| J | Osman |
| W | Osman |
| B | Osman |
| G | Osoata |
| M | Ostermann |
| E | O'Sullivan |
| S | O'Sullivan |
| MA | Oteng |
| N | Otey |
| OK | Otite |
| M | O'Toole |
| J | Ouyang |
| R | Owen |
| S | Owen |
| E | Owens |
| C | Owoo |
| Y | Owoseni |
| M | Owston |
| R | Oxlade |
| F | Ozdes |
| J | Pack |
| A | Packham |
| S | Packham |
| P | Paczko |
| G | Padden |
| A | Padmakumar |
| C | Page |
| I | Page |
| J | Page |
| S | Page |
| V | Page |
| J | Paget |
| K | Pagett |
| V | Pai |
| L | Paisley |
| S | Pajak |
| G | Pakou |
| A | Pakozdi |
| S | Pal |
| S | Pal |
| A | Palacios |
| VB | Palagiri Sai |
| V | Palaniappan |
| P | Palanivelu |
| A | Palfreeman |
| H | Palfrey |
| V | Palissery |
| D | Palit |
| S | Pallipparambil Antony |
| J | Palman |
| A | Palmer |
| H | Palmer |
| J | Palmer |
| L | Palmer |
| R | Palmer |
| A | Pambouka |
| I | Pamphlett |
| D | Pan |
| A | Pandey |
| N | Pandian |
| K | Pandya |
| T | Pandya |
| HR | Paneru |
| A | Panes |
| J | Pang |
| YW | Pang |
| R | Pangeni |
| L | Pannell |
| K | Pannu |
| S | Pant |
| S | Panthakalam |
| CT | Pantin |
| N | Pao |
| H | Papaconstantinou |
| NS | Papavarnavas |
| P | Papineni |
| K | Paques |
| AW | Paracha |
| K | Paradowski |
| V | Parambil |
| S | Paranamana |
| SR | Parashar |
| I | Parberry |
| A | Parekh |
| D | Parekh |
| L | Parfitt |
| H | Parfrey |
| O | Parikh |
| G | Parish |
| J | Park |
| V | Parkash |
| A | Parker |
| B | Parker |
| E | Parker |
| H | Parker |
| J | Parker |
| L | Parker |
| N | Parker |
| S | Parker |
| K | Parkin |
| A | Parkinson |
| M | Parkinson |
| V | Parkinson |
| C | Parmar |
| V | Parmar |
| V | Parris |
| C | Parrish |
| B | Parry |
| HC | Parry |
| S | Parslow-Williams |
| M | Parsonage |
| G | Parsons |
| J | Parsons |
| P | Parsons |
| R | Partridge |
| Z | Parvez |
| K | Parvin |
| L | Passby |
| S | Passey |
| H | Passmore |
| J | Pastrana |
| J | Patachako |
| M | Patal |
| S | Patch |
| A | Patel |
| A | Patel |
| B | Patel |
| D | Patel |
| H | Patel |
| J | Patel |
| K | Patel |
| M | Patel |
| N | Patel |
| P | Patel |
| S | Patel |
| T | Patel |
| Z | Patel |
| V | Patel |
| K | Paterson |
| S | Pathak |
| N | Pathan |
| A | Patience |
| D | Patience |
| B | Patil |
| R | Patmore |
| S | Patole |
| L | Paton |
| A | Patrick |
| G | Patrick |
| J | Patrick |
| S | Patten |
| B | Pattenden |
| C | Patterson |
| J | Patterson |
| L | Patterson |
| M | Patterson |
| R | Patterson |
| M | Pattrick |
| D | Paudel |
| K | Paudel |
| M | Paudel |
| S | Paudel |
| M | Paul |
| S | Paul |
| L | Pauls |
| S | Paulus |
| A | Pavely |
| MJ | Pavitt |
| S | Pavord |
| B | Payne |
| E | Payne |
| M | Payne |
| R | Payne |
| L | Peacock |
| S | Peacock |
| H | Peake |
| J | Pearce |
| R | Pearse |
| A | Pearson |
| D | Pearson |
| H | Pearson |
| K | Pearson |
| S | Pearson |
| SA | Pearson |
| A | Peasley |
| H | Peddie |
| S | Peebles |
| R | Peek |
| A | Peer |
| S | Peerbhoy |
| C | Pegg |
| E | Peggie |
| H | Peggie |
| S | Peglar |
| BH | Peirce |
| M | Peirse |
| C | Pelham |
| A | Pemberton |
| M | Penacerrada |
| A | Pender |
| C | Pendlebury |
| J | Pendlebury |
| R | Penfold |
| C | Penman |
| J | Penman |
| R | Penman |
| J | Penner |
| K | Penney |
| A | Pennington |
| J | Penny |
| J | Pepperell |
| R | Percival |
| A | Pereira |
| R | Pereira |
| C | Pereira Dias Alves |
| I | Perera |
| M | Perera |
| E | Perez |
| J | Perez |
| T | Perinpanathan |
| L | Periyasamy |
| E | Perkins |
| I | Pernicova |
| E | Perritt |
| A | Perry |
| E | Perry |
| M | Perry |
| TM | Perumpral |
| G | Pessoa-Amorim |
| R | Petch |
| L | Peter |
| C | Peters |
| L | Peters |
| M | Peters |
| S | Peters |
| T | Peters |
| A | Peterson |
| R | Petersen |
| L | Peto |
| I | Petras |
| B | Petrova |
| M | Petrova |
| E | Petrovics |
| T | Pettigrew |
| M | Pezard-Snell |
| P | Pfeffer |
| G | Phalod |
| NT | Pham |
| VP | Pham |
| TTH | Phan |
| M | Phanish |
| P | Phelan |
| C | Philbey |
| J | Philbin |
| A | Phillips |
| B | Phillips |
| D | Phillips |
| N | Phillips |
| P | Phillips |
| R | Phillips |
| T | Phillips |
| M | Phipps |
| M | Phipps |
| N | Phong |
| NT | Phong |
| V | Phongsathorn |
| P | Phuc |
| PV | Phuc |
| M | Phull |
| H | Phung |
| HTK | Phung |
| HM | Phuong |
| N | Phuong |
| A | Phuyal |
| AK | Phyo |
| MTT | PI |
| S | Pick |
| J | Pickard |
| C | Pickering |
| F | Pickering |
| G | Pickering |
| T | Pickett |
| J | Pickles |
| S | Pickstock |
| B | Pickwell-Smith |
| N | Pieniazek |
| C | Piercy |
| A | Pieris |
| S | Pilgrim |
| PA | Pillai |
| S | Pillay |
| L | Pilling |
| Z | Pilsworth |
| H | Pinches |
| S | Pinches |
| K | Pine |
| MT | Pinjala |
| S | Pintus |
| G | Piper |
| T | Pirani |
| M | Pitchford |
| M | Pittman |
| S | Pitts |
| N | Plaatjies |
| N | Platt |
| R | Pleass |
| M | Plowright |
| L | Plummer |
| C | Plumptre |
| J | Pobjoy |
| T | Pogreban |
| C | Poku |
| S | Poku |
| P | Polgarova |
| R | Pollard |
| L | Pollock |
| O | Poluyi |
| GJ | Polwarth |
| F | Pomery |
| IMF | Ponce |
| P | Ponnusamy |
| S | Ponnusamy |
| A | Ponnuswamy |
| I | Ponte Bettencourt dos Reis |
| S | Pooboni |
| A | Poole |
| L | Poole |
| M | Poole |
| S | Poon |
| T | Poonian |
| J | Porteous |
| M | Porteous |
| D | Porter |
| J | Porter |
| L | Porter |
| R | Porter |
| A | Posada |
| K | Postlethwaite |
| M | Potdar |
| C | Pothecary |
| N | Pothina |
| P | Potla |
| D | Potoczna |
| J | Pott |
| A | Potter |
| J | Potter |
| S | Potter |
| T | Potter |
| E | Potton |
| JB | Potts |
| J | Potts |
| K | Potts |
| K | Poudel |
| B | Poudyal |
| U | Poultney |
| K | Poulton |
| V | Poustie |
| J | Powell |
| N | Powell |
| S | Powell |
| D | Power |
| N | Power |
| S | Power |
| J | Poxon |
| E | Poyner |
| R | Poyner |
| A | Prabhu |
| S | Prabowo |
| V | Pradhan |
| G | Pradip |
| H | Prady |
| R | Prananingtias |
| A | Prasad |
| K | Prasad |
| U | Prasad |
| F | Prasanth Raj |
| S | Prasath |
|  | Prathima |
| N | Pratiwi |
| A | Pratley |
| S | Pratt |
| CB | Prayuda |
| D | Preiss |
| C | Prendergast |
| L | Prentice |
| P | Prentice |
| V | Prescott |
| L | Presland |
| C | Prest |
| S | Preston |
| M | Pretorius |
| N | Prevatt |
| S | Prew |
| A | Price |
| C | Price |
| D | Price |
| E | Price |
| K | Price |
| LJ | Price |
| N | Price |
| V | Price |
| R | Price-Eland |
| A | Priest |
| J | Prieto |
| L | Primrose |
| C | Prince |
| J | Prince |
| L | Prince |
| S | Pringle |
| M | Prior-Ong |
| V | Pristopan |
| K | Pritchard |
| L | Pritchard |
| S | Pritchard |
| V | Priyash |
| A | Procter |
| C | Proctor |
| M | Protopapas |
| R | Proudfoot |
| B | Prudon |
| D | Pryor |
| S | Pudi |
| A | Puffett |
| J | Pugh |
| L | Pugh |
| MT | Pugh |
| N | Pugh |
| R | Pugh |
| V | Puisa |
| E | Puji Lestari |
| S | Puliyakkadi |
| J | Pullen |
| K | Punia |
| S | Punnilath Abdulsamad |
| L | Purandare |
| D | Purchase |
| C | Purdue |
| R | Purdy |
| B | Purewal |
| R | Purnell |
| M | Pursell |
| G | Purssord |
| R | Purves |
| S | Purvis |
| K | Puspatriani |
| D | Putensen |
| SI | Putu |
| B | Puvaneswaran |
| A | Puxty |
| K | Puxty |
| Z | Puyrigaud |
| E | Pyart |
| E | Pye |
| M | Pynn |
| T | Qadeer |
| M | Qayum |
| C | Quah |
| S | Quaid |
| N | Quail |
| C | Quamina |
| K | Quang |
| NN | Quang |
| L | Quarm |
| G | Quartermaine |
| R | Quartey |
| T | Quasim |
| S | Quaye |
| A | Quayle |
| E | Quek |
| S | Quenby |
| P | Qui |
| X | Qui |
| V | Quick |
| J | Quigley |
| J-C | Quijano-Campos |
| J | Quindoyos |
| A | Quinn |
| J | Quinn |
| T | Quinn |
| LJ | Quist |
| Q | Quratulain |
| D | Qureshi |
| E | Qureshi |
| H | Qureshi |
| I | Qureshi |
| K | Qureshi |
| N | Qureshi |
| Q | Qurratulain |
| S | Qutab |
| DTH | Quyen |
| DTN | Quyen |
| NTH | Quyen |
| MS | Rabbani |
| S | Rabinowicz |
| M | Raceala |
| A | Rachid |
| B | Rachman |
| R | Rachman |
| L | Rad |
| J | Radford |
| L | Radford |
| J | Radhakrishnan |
| H | Rafferty |
| MY | Rafiq |
| S | Rafiq |
| C | Rafique |
| J | Rafique |
| M | Rafique |
| R | Ragatha |
| A | Raghunathan |
| A | Raguro |
| SD | Raha |
| S | Rahama |
| M | Rahardjani |
| K | Rahilly |
| F | Rahim |
| AH | Rahimi |
| HR | Rahimi |
| M | Rahman |
| SU | Rahman |
| S | Rahmany |
| P | Rai |
| S | Rai |
| L | Raisova |
| A | Raithatha |
| A | Raj |
| A | Rajagopal |
| P | Rajagopalan |
| N | Rajaiah |
| K | Rajalingam |
| A | Rajasekaran |
| A | Rajasri |
| B | Rajbhandari |
| S | Rajbhandari |
| T | Rajeswaran |
| J | Rajeswary |
| J | Rajkanna |
| I | Rajkumar |
| G | Rajmohan |
| R | Rallan |
| K | Ralston |
| M | Ralston |
| M | Ram |
| B | Ramabhadran |
| F | Ramali |
| M | Ramali |
| A | Ramanan |
| S | Ramanna |
| M | Ramasamy |
| I | Rambe |
| A | Ramchandani |
| D | Ramdin |
| J | Ramirez |
| M | Ramirez |
| G | Ramnarain |
| A | Ramnarine |
| L | Ramos |
| T | Rampling |
| S | Ramraj |
| J | Ramsay |
| A | Ramshaw |
| A | Rana |
| GF | Rana |
| N | Rana |
| R | Rana |
| A | Rand |
| J | Rand |
| H | Randheva |
| P | Ranga |
| M | Rangar |
| H | Rangarajan |
| S | Ranjan |
| H | Rank |
| P | Ranka |
| R | Rankhelawon |
| A | Rankin |
| A | Rao |
| S | Rao |
| D | Rao |
| AA | Rasheed |
| K | Rashid |
| M | Rason |
| V | Raspa |
| S | Rastogi |
| F | Rasul |
| S | Ratcliff |
| S | Ratcliffe |
| P | Rath |
| S | Rath |
| MI | Rather |
| K | Rathod |
| S | Rathore |
| A | Ratnakumar |
| J | Ratoff |
| D | Rattehalli |
| D | Ravaccia |
| M | Raval |
| P | Ravencroft |
| J | Raw |
| R | Raw |
| M | Rawal |
| SA | Rawashdeh |
| H | Rawlins |
| G | Ray |
| A | Raymond-White |
| D | Raynard |
| H | Rayner |
| N | Rayner |
| A | Raynsford |
| S | Razvi |
| Z | Razvi |
| K | Read |
| S | Read |
| M | Reay |
| A | Reddington |
| A | Reddy |
| H | Reddy |
| H | Redfearn |
| A | Redfern-Walsh |
| I | Redknap |
| N | Redman |
| A | Redome |
| J | Redome |
| A | Reed |
| J | Reed |
| A | Rees |
| C | Rees |
| H | Rees |
| J | Rees |
| M | Rees |
| S | Rees |
| T | Rees |
| E | Rees-Jones |
| F | Regan |
| K | Regan |
| M | Regan |
| S | Regan |
| K | Rege |
| A | Regmi |
| A | Rehan |
| A | Rehman |
| H | Rehman |
| S | Rehman |
| Z | Rehman |
| A | Reid |
| J | Reid |
| S | Reid |
| M | Reilly |
| S | Reilly |
| C | Reith |
| A | Reka |
| A | Remegoso |
| D | Rengan |
| L | Renouf |
| S | Renshaw |
| R | Renu Vattekkat |
| H | Reschreiter |
| M | Revels |
| A | Revill |
| G | Rewitzky |
| S | Rey |
| C | Reynard |
| D | Reynish |
| H | Reynolds |
| P | Reynolds |
| J | Rhodes |
| N | Riaz |
| P | Ribeiro |
| E | Rice |
| M | Rice |
| N | Rice |
| M | Rich |
| A | Richards |
| L | Richards |
| S | Richards |
| C | Richardson |
| E | Richardson |
| F | Richardson |
| J | Richardson |
| M | Richardson |
| N | Richardson |
| J | Riches |
| K | Riches |
| L | Richmond |
| R | Richmond |
| W | Ricketts |
| H | Rickman |
| A | Riddell |
| S | Ridgway |
| M | Ridha |
| C | Ridley |
| P | Ridley |
| G | Rieck |
| L | Rigby |
| M | Rigby |
| D | Rigler |
| S | Rijal |
| N | Rika |
| H | Riley |
| M | Riley |
| P | Riley |
| A | Rimainar |
| ZVP | Rimba |
| D | Rimmer |
| W | Rina |
| R | Rintoul |
| A | Riordan |
| D | Ripley |
| N | Rippon |
| C | Rishton |
| M | Riste |
| D | Ritchie |
| J | Ritchie |
| A | Ritchings |
| P | Rivera Ortega |
| V | Rivers |
| B | Rizvi |
| SAS | Rizvi |
| SHM | Rizvi |
| J | Robb |
| E | Robbins |
| C | Roberts |
| G | Roberts |
| I | Roberts |
| J | Roberts |
| K | Roberts |
| M | Roberts |
| N | Roberts |
| P | Roberts |
| R | Roberts |
| V | Roberts |
| C | Robertson |
| J | Robertson |
| J | Robertson |
| K | Robertson |
| N | Robertson |
| S | Robertson |
| M | Robertson |
| N | Robin |
| C | Robinson |
| E | Robinson |
| G | Robinson |
| H | Robinson |
| J | Robinson |
| K | Robinson |
| L | Robinson |
| M | Robinson |
| N | Robinson |
| R | Robinson |
| S | Robinson |
| A | Robinson |
| S | Robson |
| A | Rocca |
| L | Roche |
| S | Roche |
| N | Rodden |
| A | Roddick |
| E | Roddy |
| J | Roddy |
| M | Roderick |
| A | Rodger |
| F | Rodger |
| M | Rodger |
| M | Rodger |
| A | Rodgers |
| D | Rodgers |
| N | Rodgers |
| P | Rodgers |
| R | Rodriguez-Belmonte |
| N | Roe |
| C | Roehr |
| G | Rogers |
| J | Rogers |
| L | Rogers |
| M | Rogers |
| P | Rogers |
| S | Rogers |
| T | Rogers |
| J | Rojkova |
| KK | Roka |
| S | Rokadiya |
| L | Rollins |
| J | Rollo |
| C | Rolls |
| A | Rond-Alliston |
| C | Rook |
| K | Rooney |
| L | Rooney |
| LP | Rosaroso |
| EJ | Rosby |
| A | Rose |
| S | Rose |
| Z | Rose |
| J | Rosier |
| A | Roskilly |
| GA | Ross |
| I | Ross |
| J | Ross |
| J | Rossdale |
| A | Ross-Parker |
| A | Rostron |
| A | Rosyid |
| A | Rothman |
| J | Rothwell |
| L | Roughley |
| CA | Rourke |
| K | Rowan |
| N | Rowan |
| S | Rowan |
| A | Rowe |
| N | Rowe |
| L | Rowe-Leete |
| B | Rowlands |
| E | Rowlands |
| M | Rowley |
| S | Roy |
| M | Roycroft |
| A | Roynon-Reed |
| AR | Royson |
| S | Rozewicz |
| A | Rudenko |
| S | Rudrakumar |
| B | Rudran |
| S | Ruff |
| P | Rughani |
| R | Rule |
| S | Rundell |
| E | Rushforth |
| J | Rushmer |
| D | Rusk |
| P | Russell |
| R | Russell |
| C | Russo |
| M | Rutgers |
| K | Rutkowski |
| A | Ryan |
| B | Ryan |
| K | Ryan |
| L | Ryan |
| M | Ryan |
| P | Ryan |
| D | Ryan-Wakeling |
| E | Rybka |
| M | Ryder |
| S | Ryder |
| M | Saad |
| G | Saalmink |
| J | Sabale |
| S | Sabaretnam |
| N | Sadiq |
| E | Sadler |
| A | Saffy |
| B | Sage |
| H | Sagoo |
| S | Sagrir |
| R | Saha |
| S | Saha |
| N | Sahdev |
| S | Sahedra |
| J | Sahota |
| N | Said |
| S | Saini |
| V | Saini |
| B | Saint |
| N | Sairam |
| A | Sajid |
| S | Sakthi |
| H | Sakuri |
| M | Saladi |
| A | Salam |
| A | Salberg |
| E | Salciute |
| G | Saleeb |
| M | Saleh |
| H | Salih |
| L | Salih |
| D | Salim |
| S | Salisbury |
| S | Saliu |
| R | Salman |
| J | Salmon |
| R | Salmon |
| D | Salutous |
| M | Sam |
| S | Sam |
| T | Samakomva |
| R | Saman |
| S | Samar |
| S | Saminathan |
| R | Samlal |
| E | Sammons |
| D | Sammut |
| M | Sammut |
| S | Sammut |
| T | Sammut |
| S | Sampath |
| C | Sampson |
| J | Sampson |
| A | Samson |
| J | Samuel |
| M | Samuel |
| R | Samuel |
| TDL | Samuel |
| Y | Samuel |
| K | Samuels |
| T | Samuels |
| J | Samways |
| M | Samyraju |
| I | Sana |
| V | Sanchez |
| A | Sanchez Gonzalez |
| A | Sanda-Gomez |
| P | Sandercock |
| J | Sanders |
| A | Sanderson |
| T | Sanderson |
| K | Sandhu |
| L | Sandhu |
| S | Sandow |
| V | Sandrey |
| S | Sands |
| L | Sanga |
| H | Sangha |
| J | Sanghera |
| M | Sangombe |
| M | Sanju |
| L | Sankaran |
| F | Santos |
| C | Santos Ferreira De Almeida |
| R | Santosh |
| J | Sanyal |
| AF | Sanz-Cepero |
| Y | Sapkota |
| D | Saragih |
| D | Saralaya |
| A | Saraswati |
| A | Saraswatula |
| P | Saravanamuthu |
| S | Sarawade |
| J | Sarella |
| A | Sarfatti |
| R | Sargent |
| B | Sari |
| D | Sari |
| D | Sarkar |
| K | Sarkar |
| N | Sarkar |
| R | Sarkar |
| S | Sarma |
| P | Sarmiento |
| Z | Sarwar |
| T | Sass |
| K | Satchithananthasivam |
| S | Sathe |
| S | Sathianandan |
| A | Sathyanarayanan |
| SJP | Sathyanarayanan |
| T | Sathyapalan |
| P | Satodia |
| V | Saulite |
| A | Saunders |
| R | Saunders |
| S | Saunders |
| A | Saunderson |
| H | Savill |
| K | Savlani |
| G | Saxena |
| M | Saxton |
| A | Sayan |
| I | Sayers |
| D | Scaletta |
| D | Scanlon |
| J | Scanlon |
| L | Scarratt |
| S | Scattergood |
| A | Schadenberg |
| J | Schafers |
| W | Schneblen |
| E | Schofield |
| R | Schofield |
| S | Schofield |
| D | Scholes |
| K | Scholes |
| A | Schoolmeesters |
| N | Schumacher |
| N | Schunke |
| M | Schuster Bruce |
| K | Schwarz |
| A | Scobie |
| T | Scoones |
| T | Scorrer |
| A | Scott |
| A | Scott |
| C | Scott |
| E | Scott |
| K | Scott |
| L | Scott |
| M | Scott |
| S | Scott |
| T | Scott |
| Z | Scott |
| S | Scourfield |
| W | Scrase |
| NA | Scriven |
| A | Scullion |
| T | Scullion |
| E | Seager |
| C | Seagrave |
| R | Seaman |
| E | Sear |
| I | Seaton |
| A | Seatter |
| A | Seckington |
| J | Sedano |
| G | Seddon |
| G | Sedgwick |
| Y | See |
| MA | Seelarbokus |
| C | Sefton |
| M | Segovia |
| F | Seidu |
| G | Sekadde |
| F | Selby |
| G | Selby |
| C | Sellar |
| R | Sellars |
| K | Sellers |
| J | Selley |
| V | Sellick |
| G | Selvadurai |
| B | Selvarajah |
| H | Selvaskandan |
| SS | Selvendran |
| J | Selwyn |
| A | Semmens |
| G | Semple |
| M | Sen |
| N | Sen |
| S | Sen |
| A | Sengupta |
| N | Sengupta |
| S | Senra |
| H | Senya |
| T | Serafimova |
| E | Sernicola |
| D | Sethi |
| S | Sethi |
| N | Setty |
| A | Seward |
| T | Sewdin |
| T-A | Sewell |
| J | Seymour |
| K | Seymour |
| H | Shabbir |
| F | Shackley |
| T | Shafi |
| F | Shafique |
| A | Shah |
| A | Shah |
| B | Shah |
| H-A | Shah |
| M | Shah |
| N | Shah |
| P | Shah |
| Q | Shah |
| R | Shah |
| S | Shah |
| SH | Shah |
| W | Shah |
| S | Shahad |
| S | Shahi |
| S | Shahnazari |
| N | Shahzad |
| M | Shahzeb |
| A | Shaibu |
| Z | Shaida |
| AY | Shaikh |
| M | Shaikh |
| R | Shail |
| M | Shaji |
| M | Shakeel |
| R | Shakya |
| K | Shalan |
| M | Shameem |
| N | Shamim |
| U | Shamji |
| A | Shams |
| K | Shams |
| R | Shamsah |
| T | Shanahan |
| H | Sharaf |
| A | Sharif |
| A | Sharma |
| B | Sharma |
| M | Sharma |
| O | Sharma |
| P | Sharma |
| R | Sharma |
| S | Sharma |
| SD | Sharma |
| S | Sharma |
| S | Sharma |
| A | Sharp |
| C | Sharp |
| G | Sharp |
| K | Sharp |
| LM | Sharp |
| P | Sharratt |
| K | Sharrocks |
| S | Shashaa |
| A | Shaw |
| C | Shaw |
| D | Shaw |
| J | Shaw |
| L | Shaw |
| M | Shaw |
| TG | Shaw |
| A | Shawcross |
| J | Shawcross |
| J | Shawe |
| L | Shayler |
| S | Shedwell |
| J | Sheffield |
| Z | Shehata |
| A | Sheik |
| A | Sheikh |
| N | Sheikh |
| B | Shelley |
| S | Shelton |
| A | Shenoy |
| J | Shenton |
| S | Shephardson |
| A | Shepherd |
| K | Shepherd |
| L | Shepherd |
| S | Shepherd |
| G | Sheppard |
| R | Sheppeard |
| H | Sheridan |
| R | Sheridan |
| S | Sherridan |
| L | Sherris |
| S | Sherwin |
| S | Shibly |
| FF | Shiham |
| C | Shilladay |
| B | Shillitoe |
| D | Shingadia |
| C | Shioi |
| A | Shirgaonkar |
| K | Shirley |
| H | Shirt |
| A | Shonubi |
| J | Shoote |
| R | Shorrocks |
| R | Shortman |
| R | Shotton |
| S | Shotton |
| C | Shovelton |
| E | Shpuza |
| A | Shrestha |
| G | Shrestha |
| N | Shrestha |
| R | Shrestha |
| S | Shrestha |
| K | Shuker |
| J | Shurlock |
| J | Shurmer |
| ER | Shuvo |
| SK | Siabi |
| G | Siame |
| L | Siamia |
| M | Siaw-Frimpong |
| S | Siddavaram |
| N | Siddique |
| S | Siddique |
| S | Siddique |
| E | Siddle |
| E | Sidebotham |
| J | Sidebottom |
| R | Sievers |
| K | Siggens |
| N | Sikondari |
| I | Silanas |
| SV | Silva |
| C | Silva Moniz |
| M | Sim |
| T | Simangan |
| V | Simbi |
| R | Sime |
| G | Simmons |
| O | Simmons |
| R | Simms |
| L | Simon |
| M | Simon |
| N | Simon |
| S | Simpkins |
| A | Simpson |
| A | Simpson |
| D | Simpson |
| G | Simpson |
| J | Simpson |
| K | Simpson |
| M | Simpson |
| P | Simpson |
| T | Simpson |
| K | Simpson |
| S | Sinclair |
| C | Sing |
| A | Singh |
| C | Singh |
| D | Singh |
| J | Singh |
| L | Singh |
| M | Singh |
| N | Singh |
| P | Singh |
| S | Singh |
| P | Singhal |
| B | Singizi |
| V | Singler |
| M | Sinha |
| P | Sinha |
| S | Sinha |
| U | Sinha |
| G | Sisson |
| S | Sithiravel |
| K | Sivakumar |
| S | Sivakumar |
| D | Sivakumran |
| S | Sivanadarajah |
| P-R | Sivasothy |
| A | Skaria |
| N | Skehan |
| R | Skelly |
| O | Skelton |
| I | Skene |
| D | Skinner |
| T | Skinner |
| V | Skinner |
| A | Skorko |
| I | Skorupinska |
| M | Skorupinska |
| A | Slack |
| K | Slack |
| H | Slade |
| M | Slade |
| L | Slater |
| N | Slawson |
| R | Slingsby |
| A | Sloan |
| B | Sloan |
| D | Sloan |
| G | Sloane |
| M | Slowinska |
| B | Small |
| E | Small |
| S | Small |
| A | Smallridge |
| D | Smalls |
| KD | Smallshaw |
| A | Smallwood |
| B | Smart |
| L | Smart |
| J | Smeaton |
| C | Smit |
| A | Smith |
| C | Smith |
| D | Smith |
| E | Smith |
| H | Smith |
| I | Smith |
| J | Smith |
| K | Smith |
| L | Smith |
| M | Smith |
| MA | Smith |
| N | Smith |
| O | Smith |
| P | Smith |
| R | Smith |
| S | Smith |
| T | Smith |
| V | Smith |
| S | Smolen |
| S | Smuts |
| N | Smyth |
| A | Snell |
| D | Snell |
| L | Snell |
| A | So |
| B | So |
| M | Soan |
| RF | Sobama |
| T | Sobande |
| S | Sobowiec Kouman |
| A | Sobrino Diaz |
| B | Sohail |
| H | Sohal |
| R | Soiza |
| O | Solademi |
| B | Soleimani |
| A | Solesbury |
| M | Soliman |
| B | Solis |
| R | Solly |
| L | Solomon |
| S | Somalanka |
| C | Somashekar |
| S | Sommerfield |
| G | Soni |
| R | Sonia |
| T | Sonoiki |
| S-C | Soo |
| P | Soor |
| G | Soothill |
| J | Soren |
| A | Sothinathan |
| P | Sothirajah |
| J | Sousa |
| N | Soussi |
| D | Southam |
| D | Southern |
| I | Southern |
| L | Southern |
| SM | Southin |
| J | Southwell |
| T | Southworth |
| S | Sowden |
| J | Sowter |
| C | Spalding |
| E | Spata |
| C | Speare |
| K | Spears |
| M | Spears |
| L | Speirs |
| S | Speirs |
| M | Spence |
| N | Spence |
| B | Spencer |
| G | Spencer |
| R | Spencer |
| S | Spencer |
| T | Spencer |
| H | Spickett |
| J | Spillane |
| W | Spiller |
| K | Spinks |
| M | Spinks |
| N | Spittle |
| S | Spray |
| J | Spriggs |
| O | Spring |
| G | Squires |
| J | Squires |
| R | Squires |
| R | Sreenivasan |
| S | Sreenivasan |
| M | Sri |
| K | Sri Paranthamen |
| R | Srikantaiah |
| K | Srinivasan |
| R | Srinivasan |
| A | Srirajamadhuveeti |
| V | Srirathan |
| SK | Ssiabi |
| R | Stacey |
| S | Stacpoole |
| L | Stadon |
| WJ | Stagg |
| J | Staines |
| N | Staines |
| K | Stammers |
| R | Stanciu |
| G | Stanczuk |
| T | Standley |
| B | Staniforth |
| A | Stanton |
| L | Stanton |
| R | Staples |
| S | Stapley |
| N | Staplin |
| A | Stark |
| E | Starkey |
| DS | Starnes |
| M | Starr |
| R | Stead |
| C | Stebbing |
| C | Steele |
| H | Steer |
| J | Steer |
| V | Stefania |
| P | Stefanowska |
| F | Steffensen |
| C | Stemp |
| E | Stenson |
| A | Stephens |
| D | Stephensen |
| E | Stephenson |
| M | Sterrenburg |
| J | Stevens |
| M | Stevens |
| W | Stevens |
| A | Stevenson |
| L | Stevenson |
| S | Stevenson |
| M | Steward |
| C | Stewart |
| DA | Stewart |
| K | Stewart |
| M | Stewart |
| R | Stewart |
| J | Stickley |
| G | Stiller |
| S | Stirrup |
| S | Stock |
| A | Stockdale |
| D | Stocker |
| L | Stockham |
| P | Stockton |
| E | Stoddard |
| K | Stoffberg |
| C | Stokes |
| B | Stone |
| R | Stone |
| S | Stone |
| E-J | Stoner |
| I | Storey |
| K | Storton |
| F | Stourton |
| A | Strachan |
| C | Strait |
| E | Stratton |
| J | Stratton |
| S | Straw |
| D | Streit |
| E | Stride |
| S | Stringer |
| S | Strong-Sheldrake |
| S | Struik |
| C | Stuart |
| A | Stubbs |
| H | Stubbs |
| A | Sturdy |
| S | Sturney |
| M | Stuttard |
| C | Suarez |
| K | Subba |
| CP | Subbe |
| K | Subramaniam |
| M | Subramanian |
| V | Subramanian |
| C | Subudhi |
| R | Suckling |
| S | Sudershan |
| P | Sugden |
| PA | Suherman |
| R | Sukla |
| A | Sukumaran |
| E | Suleiman |
| A | Suliman |
| F | Suliman |
| S | Sultan |
| U | Sumardi |
| S | Sundar |
| R | Sundaram |
| R | Sundhar |
| E | Sung |
| N | Sunni |
| J | Suntharalingam |
| A | Sur |
| D | Suresh |
| N | Suresh |
| S | Suresh |
| M | Surtees |
| C | Susan |
| D | Suter |
| R | Suthar |
| H | Sutherland |
| R | Sutherland |
| S | Sutherland |
| D | Sutinyte |
| D | Sutton |
| S | Sutton |
| M | Sutu |
| M-L | Svensson |
| S | Svirpliene |
| A | Swain |
| R | Swain |
| T | Swaine |
| C | Swales |
| C | Swanson-Low |
| T | Swart |
| S | Sweetman |
| E | Swift |
| P | Swift |
| R | Swift |
| R | Swingler |
| S | Swinhoe |
| K | Swist-Szulik |
| L | Swithenbank |
| O | Syed |
| C | Sykes |
| D | Sykes |
| E | Sykes |
| L | Sylvester |
| D | Symington |
| D | Symon |
| A | Syndercombe |
| Z | Syrimi |
| J | Syson |
| G | Szabo |
| D | Szabó |
| T | Szakmany |
| N | Szarazova |
| M | Szekely |
| A | Szekeres |
| M | Szeto |
| K | Szymiczek |
| M | Tabish |
| M | Tadros |
| A | Tageldin |
| L | Tague |
| H | Tahir |
| M | Tahir |
| M | Tai |
| J | Tait |
| A | Takyi |
| P | Talbot |
| A | Talbot -Smith |
| J | Talbot-Ponsonby |
| R | Tallent |
| B | Tallon |
| A | Talukdar |
| A | Tan |
| BT | Tan |
| H | Tan |
| J | Tan |
| JS | Tan |
| K | Tan |
| WT | Tan |
| A | Tana |
| A | Tanner |
| C | Tanney |
| T | Tanqueray |
| E | Tanton |
| A | Tantri |
| T | Tanzil-Al-Imran |
| H | Tarft |
| P | Taribagil |
| O | Tarin |
| S | Tariq |
| D | Tarpey |
| E | Tarr |
| L | Tarrant |
| A | Tasiou |
| A | Tate |
| M | Tate |
| ML | Tate |
| P | Tate |
| K | Tatham |
| SS | Tavares |
| V | Tavoukjian |
| SAI | Tay |
| A | Taylor |
| B | Taylor |
| C | Taylor |
| CA | Taylor |
| D | Taylor |
| E | Taylor |
| H | Taylor |
| J | Taylor |
| K | Taylor |
| L | Taylor |
| M | Taylor |
| N | Taylor |
| R | Taylor |
| S | Taylor |
| T | Taylor |
| V | Taylor |
| M | Taylor-Siddons |
| T | Taynton |
| A | Te |
| F | Teasdale |
| J | Teasdale |
| K | Teasdale |
| J | Tebbutt |
| C | Tee |
| I | Teeluck |
| B | Tejero Moya |
| R | Tejwani |
| A | Telfer |
| V | Teli |
| J | Tempany |
| J | Temple |
| N | Temple |
| H | Tench |
| YH | Teoh |
| R | Tereszkowski-Kaminski |
| L | Terrett |
| L | Terry |
| TIM | Tesha |
| D | Tetla |
| S | Tewari |
| D | Tewkesbury |
| J | Texeira |
| C | Tey |
| PN | Thach |
| M | Thake |
| C | Thakker |
| M | Thakker |
| J | Thakrar |
| BJ | Thakuri |
| B | Thamu |
| H | Thao |
| HP | Thao |
| NN | Thao |
| N | Thao |
| A | Thapa |
| H | Thatcher |
| A | Thayanandan |
| K | Thazhatheyil |
| E | Thein |
| L | Theocharidou |
| P | Thet |
| K | Thevarajah |
| M | Thevendra |
| VTK | Thi |
| D | Thien |
| N | Thiri Phoo |
| Y | Thirlwall |
| M | Thirumaran |
| A | Thomas |
| C | Thomas |
| E | Thomas |
| H | Thomas |
| J | Thomas |
| JL | Thomas |
| K | Thomas |
| L | Thomas |
| R | Thomas |
| S | Thomas |
| T | Thomas |
| V | Thomas |
| K | Thomasson |
| R | Thomas-Turner |
| C | Thompson |
| E | Thompson |
| F | Thompson |
| H | Thompson |
| J | Thompson |
| K | Thompson |
| L | Thompson |
| M | Thompson |
| O | Thompson |
| R | Thompson |
| Y | Thompson |
| BG | Thomson |
| N | Thomson |
| P | Thorburn |
| N | Thorn |
| C | Thorne |
| N | Thorne |
| A | Thornton |
| D | Thornton |
| J | Thornton |
| R | Thornton |
| S | Thornton |
| T | Thornton |
| C | Thorpe |
| N | Thorpe |
| S | Thorpe |
| P | Thozthumparambil |
| L | Thrasyvoulou |
| H | Thraves |
| N | Thu |
| NM | Thu |
| G | Thueux |
| N | Thuong |
| P | Thu-Ta |
| D | Thuy |
| DTT | Thuy |
| V | Thwaiotes |
| C | Thwaites |
| CL | Thwaites |
| G | Thwaites |
| S | Tiberi |
| S | Tieger |
| C | Tierney |
| M | Tighe |
| S | Tilbey |
| C | Till |
| A | Tiller |
| H | Tiller |
| J | Timerick |
| E | Timlick |
| A | Timmins |
| A | Timmis |
| H | Timms |
| A-M | Timoroksa |
| S | Tinashe |
| S | Tingley |
| N | Tinker |
| H | Tinkler |
| M | Tinkler |
| J | Tipper |
| A | Tirumalai Adisesh |
| H | Tivenan |
| K | Tluchowska |
| H | T-Michael |
| A | Todd |
| J | Todd |
| S | Todd |
| O | Toffoletti |
| M | Tohfa |
| S | Tohill |
| M | Tolson |
| A | Tomas |
| N | Tomasova |
| S | Tomlin |
| S | Tomlins |
| J | Tomlinson |
| K | Tomlinson |
| J | Tonkin |
| I | Tonna |
| C | Toohey |
| K | Topham |
| M | Topping |
| A | Torokwa |
| C | Torrance |
| O | Touma |
| L | Tous Sampol |
| R | Tousis |
| M | Tout |
| P | Tovey |
| G | Towersey |
| J | Townley |
| R | Tozer |
| DK | Tran |
| H | Tran |
| HB | Tran |
| M | Tran |
| N | Tran |
| VG | Tran |
| VK | Tran |
| NTH | Trang |
| H | Tranter |
| J | Travers |
| C | Travill |
| S | Traynor |
| L | Trethowan |
| E | Treus Gude |
| M | Trevelyan |
| NA | Trewick |
| A | Tridente |
| H | Trieu |
| S | Triggs |
| F | Trim |
| A | Trimmings |
| T | Trinick |
| S | Tripathy |
| K | Trivedi |
| S | Troedson |
| E | Tropman |
| A | Trotter |
| S | Trous |
| H | Trower |
| M | Trowsdale Stannard |
| N | Trudgill |
| R | Truell |
| N | Truman |
| M | Truslove |
| S | Trussell |
| T | Trussell |
| K | Tsakiridou |
| C | Tsang |
| P | Tsang |
| T | Tsawayo |
| KK | Tsilimpari |
| G | Tsinaslanidis |
| M | Tsitsi |
| S | Tso |
| HTC | Tu |
| N | Tucker |
| S | Tucker |
| S | Tucker |
| DE | Tudor |
| A | Tufail |
| J | Tuff |
| J | Tuffney |
| R | Tully |
| T | Tulus Satriasih |
| G | Tunesi |
| D | Tung |
| DQ | Tung |
| K | Turbitt |
| R | Turel |
| T | Turgut |
| C | Turley |
| A | Turnbull |
| A | Turner |
| C | Turner |
| G | Turner |
| K | Turner |
| L | Turner |
| LC | Turner |
| M | Turner |
| P | Turner |
| S | Turner |
| V | Turner |
| I | Turner-bone |
| S | Turney |
| J | Turvey |
| NTM | Tuyen |
| C | Tweed |
| D | Tweed |
| R | Twemlow |
| E | Twohey |
| B | Tyagi |
| V | Tyagi |
| A | Tyer |
| A | Tyler |
| J | Tyler |
| A | Tyzack |
| P | Tzavaras |
| I | Tzinieris |
| AW | Uddin |
| MS | Uddin |
| R | Uddin |
| J | Ugoji |
| E | Ukaegbu |
| M | Ul Haq |
| W | Ul Hassan |
| Z | Ul-Haq |
| S | Ullah |
| J | Um |
| A | Umaipalan |
| A | Umate |
| J | Umeadi |
| A | Umeh |
| W | Umeojiako |
| B | Ummat |
| E | Underhill |
| C | Underwood |
| J | Underwood |
| A | Unsworth |
| V | Uppal |
| VS | Uppal |
| G | Upson |
| M | Ur Rasool |
| A | Uriel |
| S | Urruela |
| H | Uru |
| J | Usher |
| M | Usher |
| R | Usher |
| A | Usher-Rea |
| A | Ustianowski |
| E | Usuf |
| F | Utomo |
| H | Uzu |
| LC | Vaccari |
| U | Vaghela |
| A | Vaidya |
| D | Vail |
| B | Valecka |
| J | Valentine |
| B | Valeria |
| P | Vallabhaneni |
| T | Valleri |
| N | Vallotton |
| L | Vamplew |
| E | Vamvakiti |
| J | Vamvakopoulos |
| CTC | Van |
| S | Van Blydenstein |
| L | van Bruggen |
| M | van de Venne |
| A | van der Meer |
| N | van der Stelt |
| R | Van Doorn |
| L | van Koutrik |
| A | Van Loggerenberg |
| J | Vance-Daniel |
| R | Vancheeswaran |
| SI | Vandeyoon |
| P | Vankayalapati |
| P | Vanmali |
| C | Vansomeren |
| W | Van't Hoff |
| S | Vara |
| SJ | Vardy |
| A | Varghese |
| M | Varghese |
| W | Varney |
| G | Varnier |
| A-N | Varouxaki |
| R | Varquez |
| V | Vasadi |
| O | Vass |
| K | Vassell |
| V | Vasu |
| V | Vasudevan |
| M | Vatish |
| S | Vaughan |
| H | Vayalaman |
| D | Vayapooree |
| C | Vaz |
| N | Veale |
| S | Veerasamy |
| S | Velankar |
| L | Velauthar |
| N | Veli |
| N | Vella |
| A | Velugupati |
| A | Velusamy |
| I | Venables |
| M | Venditti |
| R | Venkataramakrishnan |
| R | Venn |
| M | Venter |
| L | Ventilacion |
| J | Vere |
| M | Veres |
| S | Vergnano |
| W | Verling |
| A | Verma |
| R | Vernall |
| B | Vernon |
| M | Vertue |
| L | Verueco |
| J | Verula |
| A | Veterini |
| N | Vethanayagam |
| S | Vettikumaran |
| L | Veys |
| C | Vickers |
| S | Victor |
| S | Victoria |
| C | Vidaillic |
| CP | Vidaillac |
| J | Vidler |
| B | Vijayakumar |
| VW | Vijayaraghavan Nalini |
| B | Vilcinskaite |
| A | Vileito |
| N | Vilimiene |
| L | Vinall |
| S | Vinay |
| L | Vinayakarao |
| O | Vincent |
| R | Vincent |
| NQ | Vinh |
| P | Virdee |
| E | Virgilio |
| AM | Virk |
| E | Visentin |
| M | Vitaglione |
| K | Vithian |
| S | Vittoria |
| S | Vivekananthan |
| E | Vlad |
| B | Vlies |
| L | von Oven |
| C | Vooght |
| KT | Vu Thai |
| K | Vutipongsatorn |
| A | Vuylsteke |
| E | Vyras |
| R | Wach |
| B | Wadams |
| S | Wadd |
| N | Waddington |
| P | Wade |
| J | Wadsley |
| K | Wadsworth |
| SEI | Wafa |
| D | Wagstaff |
| L | Wagstaff |
| D | Wahab |
| Z | Wahbi |
| A | Waheed Adigun |
| S | Waidyanatha |
| A | Waite |
| R | Wake |
| A | Wakefield |
| W | Wakeford |
| F | Wakinshaw |
| E | Waldeck |
| A | Walden |
| L | Walding |
| A | Waldron |
| J | Waldron |
| E | Wales |
| B | Wali |
| D | Walker |
| G | Walker |
| H | Walker |
| I | Walker |
| K | Walker |
| L | Walker |
| O | Walker |
| R | Walker |
| S | Walker |
| G | Wallace |
| R | Wallbutton |
| J | Wallen |
| K | Wallendszus |
| A | Waller |
| R | Waller |
| G | Wallis |
| L | Wallis |
| M | Wallis |
| E | Walmsley |
| D | Walsh |
| E | Walsh |
| L | Walsh |
| D | Walstow |
| D | Walter |
| A | Walters |
| H | Walters |
| J | Walters |
| E | Walton |
| L | Walton |
| M | Walton |
| O | Walton |
| S | Walton |
| M | Wan |
| J | Wanda |
| M | Wands |
| R | Wane |
| F | Wang |
| N | Wang |
| R | Wang |
| S | Wang |
| D | Warbrick |
| S | Warburton |
| C | Ward |
| D | Ward |
| E | Ward |
| H | Ward |
| J | Ward |
| L | Ward |
| N | Ward |
| R | Ward |
| T | Ward |
| SA | Warden |
| G | Wardere |
| S | Wardle |
| H | Wardy |
| G | Waring |
| S | Waring |
| J | Warmington |
| B | Warner |
| C | Warner |
| L | Warnock |
| S | Warran |
| J | Warren |
| L | Warren |
| R | Warren |
| Y | Warren |
| D | Warrender |
| H | Warren-Miell |
| A | Warris |
| G | Warwick |
| H | Wassall |
| S | Wasserman |
| E | Wasson |
| HJ | Watchorn |
| H | Waterfall |
| A | Waters |
| D | Waters |
| M | Waterstone |
| A | Watkin |
| C | Watkins |
| E | Watkins |
| K | Watkins |
| L | Watkins |
| A | Watson |
| AJR | Watson |
| E | Watson |
| F | Watson |
| JGR | Watson |
| L | Watson |
| P | Watson |
| R | Watson |
| K | Watson |
| M | Watters |
| D | Watterson |
| K | Wattimena |
| D | Watts |
| J | Watts |
| M | Watts |
| V | Waugh |
| E | Wayman |
| M | Wayman |
| A | Wazir |
| M | Weatherhead |
| N | Weatherly |
| C | Webb |
| H | Webb |
| K | Webb |
| S | Webb |
| C | Websdale |
| D | Webster |
| I | Webster |
| J | Webster |
| T | Webster |
| J | Wedlin |
| L | Wee |
| R | Weerakoon |
| T | Weerasinghe |
| J | Weeratunga |
| M | Weetman |
| S | Wei |
| I | Weichert |
| E | Welch |
| H | Welch |
| J | Welch |
| L | Welch |
| S | Welch |
| B | Welham |
| S | Weller |
| L | Wellings |
| B | Wells |
| S | Wellstead |
| B | Welsh |
| R | Welsh |
| I | Welters |
| R | Welton |
| V | Wenn |
| L | Wentworth |
| J | Wesonga |
| K | Wesseldine |
| J | West |
| M | West |
| R | West |
| S | West |
| L | Western |
| R | Westhead |
| H | Weston |
| A | Westwood |
| K | Westwood |
| S | Westwood |
| B | Wetherill |
| S | Wheaver |
| H | Wheeler |
| B | Whelan |
| M | Whelband |
| A | Whileman |
| A | Whitcher |
| A | White |
| B | White |
| C | White |
| D | White |
| J | White |
| K | White |
| M | White |
| N | White |
| S | White |
| T | White |
| C | Whitehead |
| K | Whitehorn |
| A | Whitehouse |
| C | Whitehouse |
| T | Whitehouse |
| J | Whiteley |
| L | Whiteley |
| S | Whiteley |
| R | Whitham |
| G | Whitlingum |
| D | Whitmore |
| E | Whittaker |
| L | Whittam |
| A | Whittington |
| H | Whittle |
| R | Whittle |
| E | Wiafe |
| L | Wiblin |
| O | Wickens |
| J | Widdrington |
| J | Wieboldt |
| H | Wieringa |
| C | Wiesender |
| L | Wiffen |
| A | Wight |
| A | Wignall |
| C | Wignall |
| A | Wilce |
| D | Wilcock |
| E | Wilcock |
| L | Wilcox |
| B | Wild |
| L | Wild |
| S | Wild |
| M | Wilde |
| L | Wilding |
| P | Wilding |
| T | Wildsmith |
| J | Wileman |
| J | Wiles |
| K | Wiles |
| E | Wilhelmsen |
| T | Wiliams |
| J | Wilkie |
| D | Wilkin |
| H | Wilkins |
| J | Wilkins |
| S | Wilkins |
| I | Wilkinson |
| L | Wilkinson |
| N | Wilkinson |
| S | Wilkinson |
| T | Wilkinson |
| S | Willetts |
| A | Williams |
| C | Williams |
| CV | Williams |
| D | Williams |
| E | Williams |
| G | Williams |
| H | Williams |
| J | Williams |
| K | Williams |
| M | Williams |
| P | Williams |
| R | Williams |
| S | Williams |
| T | Williams |
| S | Williams |
| A | Williamson |
| C | Williamson |
| D | Williamson |
| J | Williamson |
| JD | Williamson |
| R | Williamson |
| C | Williamson |
| H | Williamson |
| E | Willis |
| H | Willis |
| J | Willis |
| L | Wills |
| L | Willsher |
| C | Willshire |
| F | Willson |
| J | Willson |
| A | Wilson |
| B | Wilson |
| D | Wilson |
| I | Wilson |
| J | Wilson |
| K | Wilson |
| K-A | Wilson |
| L | Wilson |
| M | Wilson |
| S | Wilson |
| T | Wilson |
| J | Wilson |
| KLY | Win |
| M | Win |
| T | Win |
| TT | Win |
| WYW | Win |
| L | Winckworth |
| L | Winder |
| P | Winder |
| S | Winearl |
| H | Winmill |
| S | Winn |
| C | Winpenny |
| H | Winslow |
| H | Winter |
| J | Winter |
| B | Winter-Goodwin |
| J | Winterton |
| H | Winwood |
| J | Wischhusen |
| S | Wisdom |
| M | Wise |
| M | Wiselka |
| R | Wiseman |
| S | Wiseman |
| S | Wishart |
| T | WIshlade |
| E | Witele |
| N | Withers |
| J | Wittes |
| D | Wixted |
| T | Wodehouse |
| W | Wolf |
| N | Wolff |
| K | Wolffsohn |
| R | Wolf-Roberts |
| E | Wolodimeroff |
| A | Wolstencroft |
| A | Wong |
| C | Wong |
| C-H | Wong |
| C-M | Wong |
| E | Wong |
| JSY | Wong |
| KY | Wong |
| MY | Wong |
| N | Wong |
| S | Wong |
| T | Wong |
| AA | Wongkyezeng |
| A | Wood |
| C | Wood |
| D | Wood |
| F | Wood |
| G | Wood |
| H | Wood |
| J | Wood |
| L | Wood |
| M | Wood |
| S | Wood |
| T | Wood |
| K | Woodall |
| R | Woodfield |
| C | Woodford |
| E | Woodford |
| J | Woodford |
| L | Woodhead |
| T | Woodhead |
| P | Woodland |
| M | Woodman |
| S | Woodmansey |
| C | Woods |
| J | Woods |
| K | Woods |
| S | Woods |
| Z | Woodward |
| M | Woolcock |
| G | Wooldridge |
| R | Woolf |
| C | Woollard |
| L | Woollen |
| E | Woolley |
| J | Woolley |
| D | Woosey |
| D | Wootton |
| J | Wootton |
| D | Worley |
| S | Worton |
| J | Wraight |
| M | Wray |
| K | Wren |
| L | Wren |
| C | Wrey Brown |
| C | Wright |
| D | Wright |
| F | Wright |
| H | Wright |
| I | Wright |
| L | Wright |
| R | Wright |
| S | Wright |
| T | Wright |
| C | Wroe |
| H | Wroe |
| H | Wu |
| P | Wu |
| J | Wubetu |
| F | Wulandari |
| R | Wulandari |
| S | Wurie |
| C | Wyatt |
| F | Wyn-Griffiths |
| I | Wynter |
| B | Xavier |
| A | Xhikola |
| BE | Xia |
| Z | Xia |
| E | Yacoba |
| S | Yadav |
| M | Yakubi |
| M | Yan |
| Y | Yanagisawa |
| F | Yang |
| Y | Yang |
| M | Yanney |
| WL | Yap |
| N | Yaqoob |
| S | Yasmin |
| B | Yates |
| D | Yates |
| E | Yates |
| H | Yates |
| T | Yates |
| M | Yates |
| J | Ye |
| C | Yearwood Martin |
| K | Yein |
| F | Yelnoorkar |
| L | Yen |
| LM | Yen |
| A | Yeoh |
| CY | Yeung |
| P | Yew |
| D | Yewatkar |
| L | Ylquimiche Melly |
| I | Ynter |
| H | Yong |
| J | Yorke |
| J | Youens |
| A | Younes Ibrahim |
| E | Young |
| G | Young |
| L | Young |
| A | Yousafzar |
| S | Youssouf |
| A | Yousuf |
| H | Yovita |
| C | Yu |
| JSJ | Yuan |
| N | Yufaniaputri |
| B | Yung |
| D | Yusef |
| S | Yusef |
| I | Yusuf |
| A-S | Zafar |
| S | Zagalo |
| S | Zaher |
| A | Zahoor |
| M | Zainab |
| T | Zak |
| K | Zaki |
| N | Zakir |
| K | Zalewska |
| A | Zamalloa |
| M | Zaman |
| S | Zaman |
| J | Zamikula |
| L | Zammit |
| M | Zammit-Mangion |
| M | Zawadzka |
| M | Zayed |
| E | Zebracki |
| D | Zehnder |
| L | Zeidan |
| D | Zeinali |
| J | Zhang |
| X | Zhao |
| D | Zheng |
| D | Zhu |
| M | Zia |
| O | Zibdeh |
| R | Zill-E-Huma |
| ET | Zin |
| E | Zincone |
| G | Zindoga |
| E | Zinkin |
| V | Zinyemba |
| C | Zipitis |
| L | Zitter |
| A | Zmierczak |
| G | Zubikarai |
| A | Zubir |
| N | Zuhra |
| R | Zulaikha |
| S | Zulfikar |
| C | Zullo |
| A | Zuriaga-Alvaro |
